# Nanoplatforms for Targeted Stimuli-Responsive Drug Delivery: A Review of Platform Materials and Stimuli-Responsive Release and Targeting Mechanisms

**DOI:** 10.3390/nano11030746

**Published:** 2021-03-16

**Authors:** Yuzhe Sun, Edward Davis

**Affiliations:** Materials Engineering Program, Mechanical Engineering Department, Auburn University, 101 Wilmore Drive, Auburn, AL 36830, USA; yzs0048@auburn.edu

**Keywords:** nanoparticles, drug delivery, drug targeting, release stimuli, endogenous stimuli, exogenous stimuli

## Abstract

To achieve the promise of stimuli-responsive drug delivery systems for the treatment of cancer, they should (1) avoid premature clearance; (2) accumulate in tumors and undergo endocytosis by cancer cells; and (3) exhibit appropriate stimuli-responsive release of the payload. It is challenging to address all of these requirements simultaneously. However, the numerous proof-of-concept studies addressing one or more of these requirements reported every year have dramatically expanded the toolbox available for the design of drug delivery systems. This review highlights recent advances in the targeting and stimuli-responsiveness of drug delivery systems. It begins with a discussion of nanocarrier types and an overview of the factors influencing nanocarrier biodistribution. On-demand release strategies and their application to each type of nanocarrier are reviewed, including both endogenous and exogenous stimuli. Recent developments in stimuli-responsive targeting strategies are also discussed. The remaining challenges and prospective solutions in the field are discussed throughout the review, which is intended to assist researchers in overcoming interdisciplinary knowledge barriers and increase the speed of development. This review presents a nanocarrier-based drug delivery systems toolbox that enables the application of techniques across platforms and inspires researchers with interdisciplinary information to boost the development of multifunctional therapeutic nanoplatforms for cancer therapy.

## 1. Introduction

Despite the numerous advances in diagnosis and treatment that have reduced death rates in many developed nations, cancer remains the second leading cause of death globally, responsible for roughly one of every six deaths [1]. Pioneering studies of drug delivery systems (DDSs) for cancer treatment focused on sustained-release systems implanted when tumors were resected. These systems released drugs at a constant rate, enabling the physiological concentration to remain within the therapeutic window for an extended period [2,3,4,5]. However, advances in oncology suggest additional requirements for drug delivery systems. While the small-molecule antineoplastic drugs used in chemotherapy are effective at killing cancer cells, issues such as short circulation half-lives, non-specific cytotoxicity, poor water solubility, vulnerability to chemical and biological degradation in the physiological environment, and severe adverse impacts on healthy tissues limit their efficacy [6,7]. More recently, advances in molecular biology, bioinformatics, and immunology have enabled the development of a series of new antineoplastic agents and biologics [8]. Biologics have reduced toxic side effects compared to traditional antineoplastic drugs; however, they can overstimulate the immune system, leading to toxicity and inflammation [9,10,11]. Additionally, as biologics are mostly composed of proteins and nucleic acids, a change in conformation impairs their function, and they are susceptible to chemical and enzymatic degradation [12,13]. Well-engineered nanocarrier-based DDSs should enhance efficacy and reduce side effects by (1) protecting payloads from degradation and extending their circulation half-lives, (2) enhancing preferential uptake by tumor cells and reduce uptake by non-cancerous cells, and (3) releasing the active payload only when an appropriate stimulus is applied. The ideal nanocarrier-based DDS not only protects the cargo but enables control over both the timing and location of release. These systems should be able to be directed to a specific tissue or cell type and release the payload only when exposed to cancer-specific physiology. In addition, along with conventional small molecule drugs, DDSs that can effectively deliver macromolecule biologics are needed. These new requirements have stimulated significant research on nanoplatforms, targeting techniques, and the use of both exogenous and endogenous stimuli to control the release of therapeutics.

Despite the significant amount of work performed, thousands of DDSs studies are reported every year, the number of Food and Drug Administration (FDA) approved DDSs for cancer therapy is very limited [14]. The reasons for this disconnect are multifold. Therapeutic efficiency is significantly impacted by the complex interactions between the nanocarrier DDS and the physiological environment. Clearance by the immune and excretory systems, unintended accumulation in the liver and spleen, as well as difficulties in tumor accumulation and cancer cell uptake, all impact the efficacy of injectable systems. There are often inherent trade-offs in performance. Lysolipid-based liposomes possess a high thermo-sensitivity, quickly releasing their payload when stimulated; however, they exhibit stability issues and significant undesired leakage of the payload [15]. Inorganic systems possess stable structure and limited drug leakage; however, degradation and excretory pathways are a concern [7,16]. The rigidity of these materials is unfavorable for optimizing biodistribution. On the other hand, while flexible materials can improve biodistribution and bio-degradable materials can address concerns with excretory pathways, these systems often exhibit premature release of payloads [17,18,19,20,21]. There are also often issues with targeting strategies. For example, one method employed utilizes the enhanced permeability and retention (ERP) effect that arises from the unique vascularity and permeability of typical cancer tissues. However, the highly heterogeneous characteristics of tumors result in less than optimal uptake. In addition, the incorporation of target ligands does not work when receptors are not upregulated as expected [6,22]. Systems that combine different approaches or materials can potentially address many of these issues. Unfortunately, combining multiple approaches is not a straightforward task. It requires significant interdisciplinary knowledge and broad insight of not only the materials and techniques employed but also how they interact with each other, the therapeutic agent, and the physiological environment [22,23]. This review is intended to contribute to the development of nanocarrier-based DDSs by reducing the knowledge barriers that exist about the plethora of techniques employed for stimuli activated release and how they are applied to the wide range of materials used as nanocarriers. Several extensively studied nanomaterials, their application to DDSs, and efforts to address performance limitations are highlighted. The principles of activated release strategies and their application to nanocarrier DDSs are reviewed. Particular attention is given to developments reported in the last ten years. Examples of how combining multiple approaches can overcome drawbacks in some DDSs are provided. The review also includes critical factors that influence the interactions of DDSs with the biological environment, particularly those affecting bio-distribution. In summary, this review presents a toolbox for nanocarrier-based DDSs that enables the application of techniques across platforms and inspires researchers with interdisciplinary information to boost the development of multifunctional therapeutic nanoplatforms for cancer therapy.

## 2. Nanocarrier Platforms and Binding Strategies

Therapeutic agents can be carried by DDSs (1) as an encapsulated payload physically bound by a nanocapsule, (2) as a covalently bound constituent, conjugated systems, and (3) as absorbed species stabilized via noncovalent interactions. It is important to note that these classifications are not exclusive; for example, drugs can be present as absorbed species in an encapsulating nanocarrier. However, they are useful for organizing DDS literature. For nanocapsules, release occurs via breakage of the membrane or more slowly via diffusion. For conjugated systems, cleavage of the covalent bond releases the active agents. In contrast, desorption, followed by diffusion, is the primary release mechanism for compounds loaded via noncovalent interactions. In this section, the major classes of materials, the general synthesis and drug loading strategies used for each, the material properties that can be leveraged to imbue stimuli-responsive release, and the advantages and disadvantages of each are discussed. The specific techniques used to enable stimuli-responsive drug release are discussed in Section 3.

### 2.1. Polymeric Micelles

**Figure 1:**
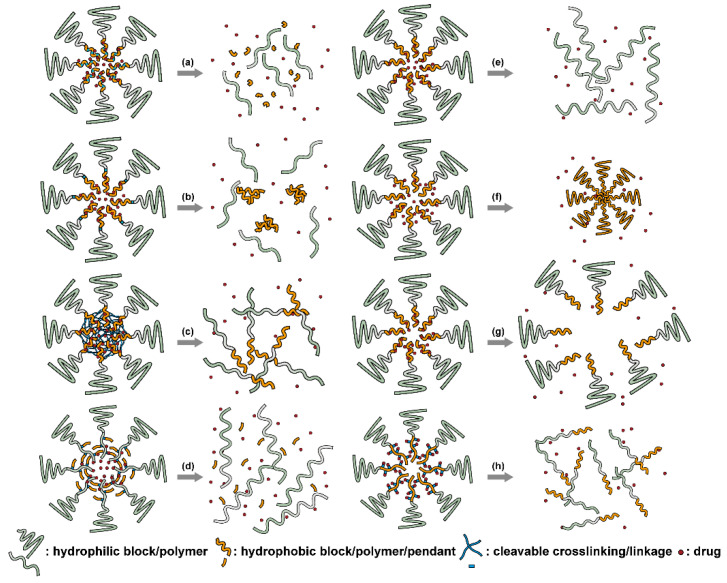
There are several strategies for stimuli-responsive micelle-based drug delivery systems (DDSs). Cleavable bonds can be used (**a**) on the hydrophobic backbone, (**b**) as the hydrophilic-hydrophobic connection, and (**c**) as crosslinks between amphiphilic molecules. Breaking these bonds results in the breakup of the micelle. Altering the amphiphilic nature of the materials (**d**) by breaking bonds that connect hydrophobic pendants to moderately hydrophobic blocks, or via stimuli-responsive (**e**) hydrophobic or (**f**) hydrophilic blocks results in micelle break up or collapse. (**g**) Micelles can be disrupted mechanically. (**h**) Payloads can be covalently bonded to the amphiphilic molecules. Breaking these bonds results in payload release and micellar breakup.

Micelles (Figure 1) are self-assembled structures formed when the concentration of amphiphilic molecules exceeds the critical micelle concentration (CMC) [24]. The core-shell structure of micelles is maintained by a thermodynamic equilibrium resulting from both hydrophobic and hydrophilic interactions. Micelles are small in size; their hydrodynamic diameters are typically 5–100 nm [25]. Due to the mild conditions required, the payload can be encapsulated during micelle synthesis [26,27]. However, stability in physiological environments remains a concern. When the concentration of the amphiphilic molecules that comprise the micelle is lower than the CMC, the micelles will dissociate. When injected into the body, the significant dilution of the micellar system coupled with an increase in the CMC due to changes in temperature, pH, and salinity and the presence of other amphiphilic molecules such as plasma proteins can destabilize the micelles and induce the release of the payload. Methods used to stabilize micelles include increasing the length of hydrophobic blocks and reducing the length of hydrophilic blocks [28,29,30]. These methods can lead to increased micelle size, which is unfavorable for the extension of circulation half-life and the utilization of enhanced permeability and retention effect (see Section 2.6.1 below) for tumor targeting. Unstable micelles are formed if the hydrophilic block is too short [31]. More commonly used methods for increasing micelle stability are to incorporate covalent crosslinking and increasing the number and strength of intermolecular interaction (such as optimized topology, π-π interaction, and host–guest complexation) in the core or shell of micelles [32,33,34,35,36,37,38,39]. When pH- [40,41,42], redox- [43,44,45,46], enzyme- [47], or photosensitive [48] bonds are used to stabilize the micelle, the corresponding stimuli can cause micelle swelling or dissociation and release of the payload (Figure 1a–c). Alternatively, processes such as protonation or temperature-induced changes to hydrophobicity can also be used to cause release for systems stabilized by intermolecular interactions (Figure 1d–f) [49,50,51,52,53,54]. Micellar stability can also be impacted by exogenous stimuli such as ultrasound (US) (Figure 1g) [46,55,56,57,58,59,60,61]. Drugs can also be conjugated to the amphiphile, usually to the hydrophobic block, via stimuli-responsive bonds (Figure 1h) [62].

In addition to conventional micelles fabricated by amphiphilic polymers, micelles have also been formed from supramolecules. In a supramolecule, noncovalent interactions are used to produce amphiphilic structures [63,64]. In these materials, the hydrophilic and hydrophobic chains terminate in groups that form strong noncovalent interactions such as host–guest interactions and electrostatic attraction between ionizable substituents. The amphiphilic structure arises due to conjugation between these groups [65]. Drug loading can be facilely conducted through self-assembly as the micelles are formed. All of the previously mentioned techniques to stabilize micelles and stimulate drug release from them can be applied to supramolecular micellar structures [66,67,68,69]. Drug release can also be initiated by the disruption of the noncovalent conjugation and triggered by temperature elevation, protonation due to pH reduction, competitive binding, and metal ion chelating in supramolecular-based systems [70,71]. As these are relatively new materials, the investigation of toxicity, biodegradability, and pharmacokinetics is very limited. The stability of the noncovalent joints also remains a concern, especially in the complicated physiological environment.

### 2.2. Liposomes

**Figure 2:**
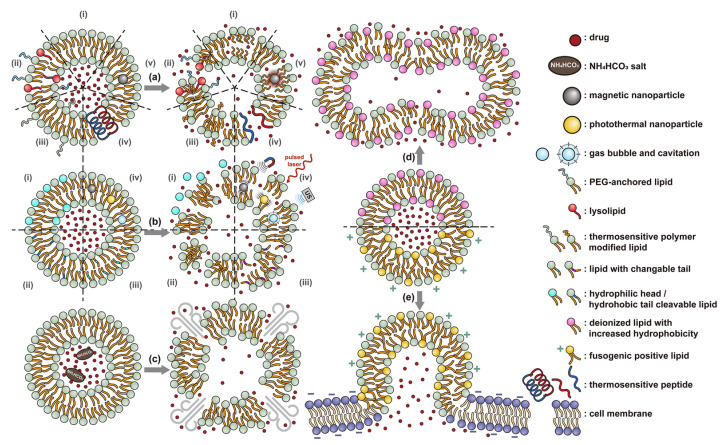
There are several strategies for stimuli-responsive liposomes. (**a**) Thermally induced effects such as (i) gel-fluid transitions (melting) in the bilayer, (ii) pore formation, induced and stabilized by lysolipids and PEG-conjugated lipids, (iii) insertion of dehydrated thermoresponsive polymers, (iv) pore formation due to conformational changes of peptides in the lipid membrane, and (v) melting of the bilayer promoted by the heat released from embedded magnetic nanoparticles in an applied AMF all serve to increase the bilayer permeability and initiate release. (**b**) Destabilization of the lipid packing via (i) cleavage of the connection between the lipophilic tails and hydrophilic head groups, (ii) cleavage of one of the lipophilic tails, (iii) isomerization or oxidation of the lipophilic tail, and (iv) cavitation induced by external agitation (vibration of magnetic nanoparticles in AMF, the photoacoustic effect, and ultrasound-generated cavitation) also initiates release. The release can also be initiated (**c**) by the disruption caused by gas generated within the liposome and during fusion with other liposomes (**d**) or cell membranes (**e**).

Liposomes (Figure 2) are self-assembled vesicles with bilayer membranes composed of lipids serving as vessel walls. As they are composed primarily of phospholipids, liposomes tend to be biodegradable and biocompatible. They initiate little to no immunogenic response. The similarity between this structure and that of cell membranes suggests their use in DDSs. Liposome-based DDSs are primarily produced by two methods: hydration of a lipid film and reverse-phase evaporation. In the first method, a lipid-containing solution is dried to form a film, which is then hydrated while being agitated to assemble liposomes [72,73]. In the second process, a solution of the lipid in an organic solvent is mixed with an aqueous solution to form a lipid-stabilized water-in-oil emulsion. As the organic solvent is evaporated from the system, it transforms into lipid bilayer vesicles [74]. In either case, the payload can be incorporated in the DDS by dissolving it in the aqueous phase used during liposome production [75]. Drugs can also be loaded after the liposome is formed as long as a suitable chemical potential gradient is established across the lipid bilayer [76,77,78,79,80,81]. As the payloads diffuse into the liposome core, they react with “trapping” agents already present in the liposome, preventing their counter diffusion and leading to their accumulation [82,83,84]. This technique, known as remote loading, can increase the loading capacity and reduce issues with leakage of the payload [85,86]. Compared with other DDSs, a unique advantage of liposomes is the capability to deliver both hydrophilic compounds loaded in the core and hydrophobic compounds loaded in the bilayer. This feature enables the co-delivery of combinational formulations to achieve a synergic effect [87]. However, liposomes’ utility for delivering lipophilic or amphiphilic drugs is more limited, as these tend to diffuse across the lipid bilayer [7].

Similar to micelles, stability is a concern in liposome-based DDSs. Many lipids have a relatively low melting point (T_m_), and body temperature increases the fluidity of the membrane, enhancing payload leakage [15]. Liposomes also have a relatively short shelf-life due to issues with fusion, aggregation, and drug leakage during storage [87]. Lyophilization can enhance liposomes shelf stability and simplifies preparation when administered [88]. However, unsaturated phospholipids are vulnerable to oxidation in vivo, which may lead to the disruption of lipid bilayers [87]. Increasing the amount of saturated phospholipid in the system can mitigate this issue [87,89,90]. The addition of cholesterol has been found to enhance drug retention and stability and to reduce the rate of opsonization in physiological environments [91,92,93,94]. However, too much cholesterol may impair stimuli-responsive behavior. In Gaber et al.’s work, the thermo-responsive behavior was almost eliminated when 30 mol% cholesterol was used [95]. A reduction in liposome aggregation and fusion can be achieved by decorating the outer surface with a protective component such as poly(ethylene glycol) (PEG) [96,97]. Modification is typically accomplished via the incorporation of a lipid conjugated with the appropriate protective group during the formation of the liposome or by modifying the lipids in the outer layer of the bilayer after liposome formation [98,99,100]. However, in the first technique, some of the protective lipids may be inserted into the inner bilayer, where it does not help reduce liposome aggregation and can reduce the payload capacity. The membrane fluidity is also impacted by the incorporation of protective lipids in both the outer and inner layers [101]. While the second approach minimizes some of these issues, it requires multiple steps and can introduce undesired residual groups to the liposomes. A unique post-insertion modification technique has been developed, in which protective lipid micelles and the unprotected liposomes are incubated together at a temperature close to the melting point of the lipids. Under these conditions, the protective lipid can spontaneously transfer from the micelle to the outer lipid layer [102]. This method efficiently inserts protective lipids onto only the outer layer of the lipid membrane, preserving the membrane fluidity and limiting issues with residual reactive groups associated with chemical modification of the lipids [98,101]. This technique can also be applied to achieve a wide range of other functional modifications [100,102,103].

**Figure 3:**
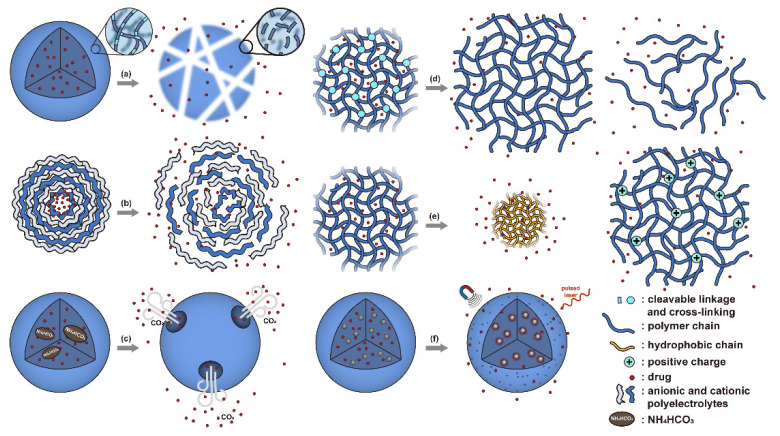
Mechanisms for release of therapeutic compounds from solid polymeric nanoparticles and nanogels. (**a**) Cleavage of bonds in the polymer chain results in degradation and dissolution. (**b**) Protonation reduces polyelectrolyte interactions weakening the structure of particles produced via LbL techniques. (**c**) The decomposition of NH_4_HCO_3_ produces CO_2_ gas rupturing the particle. (**d**) Cleavage of crosslinkers in nanogel networks results in swelling or dissociation. (**e**) Temperature elevation results in increased hydrophobicity and shrinkage of a nanogel, while protonation increases hydrophilicity and results in increased swelling. (**f**) AMF or NIR irradiation can be used to heat and agitate the system increasing payload release.

The instability of liposomes suggests three primary mechanisms for initiating drug release: (1) the creation of pores or cracks in the bilayer to enhance permeability (Figure 2a), (2) destabilizing the liposome by mechanical disturbance, via chemical degradation, or gas disruption (Figure 2b,c), and (3) initiating the fusion of the liposome with other liposomes or biological membranes (Figure 2d,e). When low-melt point lipids are used in the formulation, body temperature or local hyperthermia can be sufficient to melt the bilayer [104,105,106]. As the bilayer transitions from a gel to melt, pores form, significantly increasing the diffusion rates across it. Pore formation can be enhanced by the incorporation of lysolipids, lipids with thermally responsive polymer groups attached, and by the inclusion of peptides in the bilayer. US and alternating magnetic fields (AMF) are two commonly used strategies to disrupt liposomes via mechanical agitation [107,108,109,110,111,112]. When AMF is used, appropriate sensitizers such as iron nanoparticles need to be incorporated into the liposomes. Perfluoropentane emulsions can be encapsulated in liposomes to generate gas with US disrupting the structure [113,114,115]. Incorporation of lipids that degrade under appropriate pH, redox, enzymatic, or other conditions can also be used to destabilize the bilayer under those conditions [108,110,112,116,117,118,119].

The payload can also be released when liposomes fuse with each other or with biological membranes, (Figure 2d,e) [120,121,122,123,124]. Compared with the endocytosis pathway, liposome fusion with the cell wall bypasses the extracellular excretion and endo-lysosomal escape issues and releases payloads directly into the cytoplasm [125,126,127]. This process avoids endosomal degradation of the payload, which can be significant for macromolecular biologics [128,129]. A widely accepted mechanism for this behavior, the “flip-flop” of lipids, was proposed by Szoka’s group [130,131]. Several strategies, such as using cationic lipids [130,132], lipids that can adopt non-bilayer phases (such as 1,2-dioleoyl-sn-glycero-3-phosphoethanolamine (DOPE) [133]), decorating the surface with cell-penetrating peptides (like KALA [125] and TAT [134]), including phage fusion proteins [135], and incorporating aromatic compounds [136] in lipid bilayers have been demonstrated to improve the fusion efficiency. Reshtnyak et al. utilized the conformational changes in a fusogenic peptide that occurred at low pH as a mechanism to enhance fusion between liposomes and cells [137,138,139,140,141]. Alternatively, the fusogenic compounds can be deactivated via covalent bonding and reactivated via stimuli-induced cleavage of the bond [120,121,122,123,124].

### 2.3. Polymeric Nanoparticles

Nanoparticles formed from high molecular weight (MW) polymer can be used to encapsulate therapeutic agents (Figure 3). They are typically produced during a polymerization process or via precipitation from polymer solutions [142]. Emulsion or microemulsion polymerization can be used to create solid polymer nanoparticles. However, the residual initiator, stabilizer, and catalyst can remain and pose safety concerns. Therefore, precipitation and coacervation techniques are more widely used for biomedical applications. In these processes, the dissolved polymer undergoes precipitation, typically through the addition of a non-solvent, or coacervation, due to neutralization or complexing with electrolytes, to form solid polymeric nanoparticles [143]. Polymeric nanoparticles are generally more stable than micelles and liposomes, particularly those based on covalently bonded networks. However, they are usually susceptible to the rapid formation of a protein corona on their surface that enhances clearance by the immune system [144]. PEGylation of the surface can reduce this issue.

In addition to solid polymeric nanoparticles, hydrogel nanoparticles, also called nanogels, have also been investigated as a nanoparticle-based DDS. Nanogels are composed of networked hydrophilic polymers and can be prepared via similar methods to those used for traditional solid polymer nanoparticles [145,146]. In emulsion-based synthesis, a multifunctional monomer is included to allow the formation of a covalently crosslinked structure. In contrast, for precipitation and coacervation techniques, precursors with functional groups capable of forming physical crosslinks are used. Typically, hydrophobic–hydrophobic interactions and electrostatic interactions are used to allow for self-assembly of the hydrogel nanoparticle via precipitation techniques [145]. Generally, the networks formed via physical crosslinking are not as stable as those formed via covalent crosslinking.

Therapeutics can be loaded into solid polymeric nanoparticles and nanogels via two approaches—incorporation during nanoparticle production and adsorption [147,148,149]. One advantage over micelles and liposomes is that moisture-sensitive payloads can be loaded from nonaqueous phases during the synthesis of polymeric nanoparticles, protecting them from degradation during DDS preparation and transport within the body. For further discussion of the principles, preparation, and biomedical applications of polymeric nanoparticles and nanogels, the reader is referred to several excellent reviews [150,151,152,153].

Conventionally, polymeric nanoparticles have been used for sustained release applications [154]. When biodegradable polymers are used, the release can last for over a month as the polymer hydrolyzes or is enzymatically degraded [149]. When stimuli-responsive groups are used, payload release can be significantly faster. Similar to micelles, breaking cleavable bonds can be used to initiate the disintegration of polymeric nanoparticles and nanogels (Figure 3a,d). Various stimuli, including pH [155,156], enzymes [19,157,158,159,160,161,162], redox agents [163,164,165,166,167,168], and photoirradiation [169,170,171,172], have been used to cleave bonds located on the polymer backbone or crosslinking site. When polyelectrolytes are used, protonation under acidic conditions weakens the ionic bonding resulting in the dissolution of nanoparticles and drug release (Figure 3b) [157,173,174,175]. For nanogels, protonation (or dehydration)-induced osmotic pressure changes [176,177] and hydrophilicity changes [21,178] can also be used to induce nanogel swelling or collapse and promote drug release, (Figure 3e). Recently, Liu et al. incorporated NH_4_HCO_3_ in poly(lactic-co-glycolic acid) (PLGA) nanoparticles, (Figure 3c) [179]. CO_2_ gas was generated with sufficient pressure to rupture the nanoparticle and release the payload under acidic conditions. Finally, remote heating via AMF or near-infrared (NIR) irradiation can increase the diffusion rates of payload from these systems, (Figure 3f).

### 2.4. Porous Inorganic Nanocarriers

**Figure 4:**
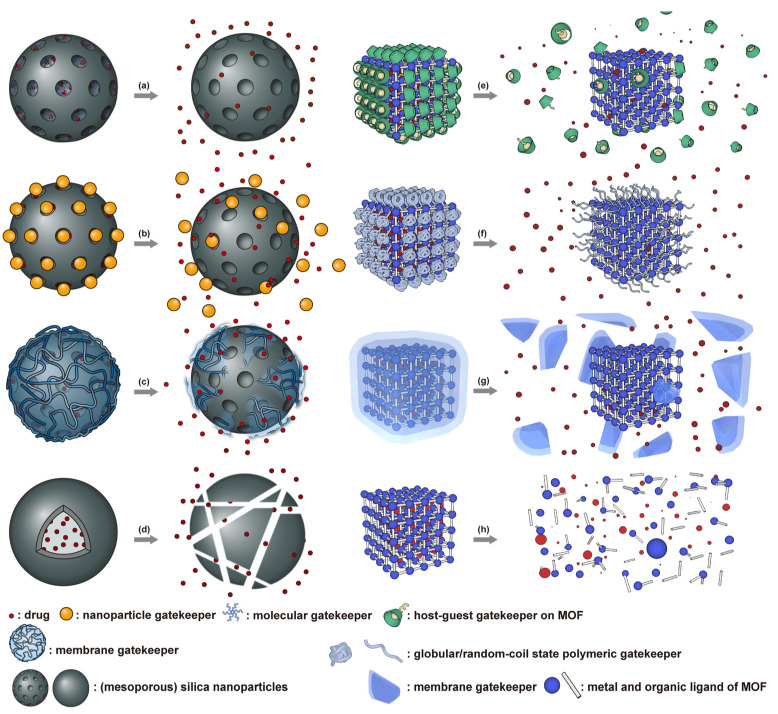
Molecular (**a**) or nanoparticle-based (**b**) gatekeepers can be used to block the pores in MSN-based DDSs. Gatekeepers based on supramolecular host–guest interactions (**e**) and polymers with a collapsed morphology are used on MOF-based DDSs. Polymer shells can serve as a gatekeeper for both systems (**c**,**g**). Removal or disruption of the gatekeepers enabling the diffusion of the payload from the pores. The MSN (**d**) or MOF (**h**) structure can also be disrupted, releasing the payload.

Mesoporous silica nanoparticles (MSN) [180] and metal-organic frameworks (MOF) [181] (Figure 4) are the most studied porous inorganic nanostructures. MSNs are synthesized via the condensation of tetraethyl orthosilicate (TEOS) in the presence of surfactant as templates [182,183]. These nanoparticles have a large surface area and tunable pore size. Issues with biodegradability hinder the development and application of MSN systems [7]. Degradation typically takes several weeks [184], and nanoparticles can accumulate in organs like the liver and spleen [185]. MSN accumulation due to repeated administration may lead to liver injury [186]. To enhance biodegradability, several strategies have been explored. Shen et al. synthesized large-pore, thin-wall MSNs with a high Q^3^/Q^4^ silicon ratio (the ratio of silicon in (HO)Si(OSi)_3_ versus in Si(OSi)_4_) [187]. These materials exhibited higher proton mobility in mesopore channels and can be degraded entirely in 24 h in simulated body fluid [188]. MSN degradation can also be accelerated by the inclusion of biodegradable [189,190,191,192,193,194,195,196,197,198] or water-soluble components [199]. To minimize premature release, pores can be blocked by gatekeepers. Three gatekeeper strategies have been developed: (1) nanoparticles attached to the pore openings via covalent bonds [200,201,202], (2) organic molecules, usually having a large dimension like β-cyclodextrin and pillararene, anchored at pores via covalent bonding or noncovalent interactions [182,203,204,205,206,207,208,209], and (3) membranes surrounding the MSN [210,211,212]. Systems that use temperature changes [204], magnetic fields [213], ultrasound [214], photoirradiation [211], pH changes [202,206], redox agents [200,201,207,210], and enzymes [205,208,212] to break the bonds attaching the gatekeeper or to disrupt the membrane have been developed (Figure 4a–c). Silica shells can also be degraded or mechanically disrupted to release the encapsulated payload (Figure 4d) [198,213].

MOF is an emerging hybrid nanostructure synthesized by the coordination of metal ions and organic ligands [181]. These systems have a highly ordered and tunable porous structure endowing them with a large surface area for drug adsorption [215,216]. Drugs can be incorporated during [217] or after synthesis [218,219]. Payload release can be controlled via gatekeepers, similarly to the approaches used for MSNs, (Figure 4e,f). Stimuli such as pH [71,180,181], glutathione (GSH) [180], salinity [70,71,220], temperature changes [71,220], photoirradiation [221], and AMF [222] have all been studied to trigger the detachment of gatekeepers from MOFs. Membrane gatekeepers have also been explored for MOF systems (Figure 4g). Payload release can also be stimulated by disruption of the MOF structure by weakening the metal ion–ligand interactions which hold the system together (Figure 4h). Techniques that use pH labile bonds [223,224,225,226], redox-cleavable ligands [227,228,229], and azobenzene-bearing organic ligands [230] have been explored. This approach has been extended to systems that use a drug [231], a prodrug [232,233], or another therapeutic agent (photothermal [234], photodynamic [235], or imaging [236]) as the organic ligand in the MOF structure, eliminating the need for a gatekeeper; any stimulus that disrupts the ligand metal coordination acts to release the drug from the MOF.

### 2.5. Systems Not Based on Encapsulation

**Figure 5:**
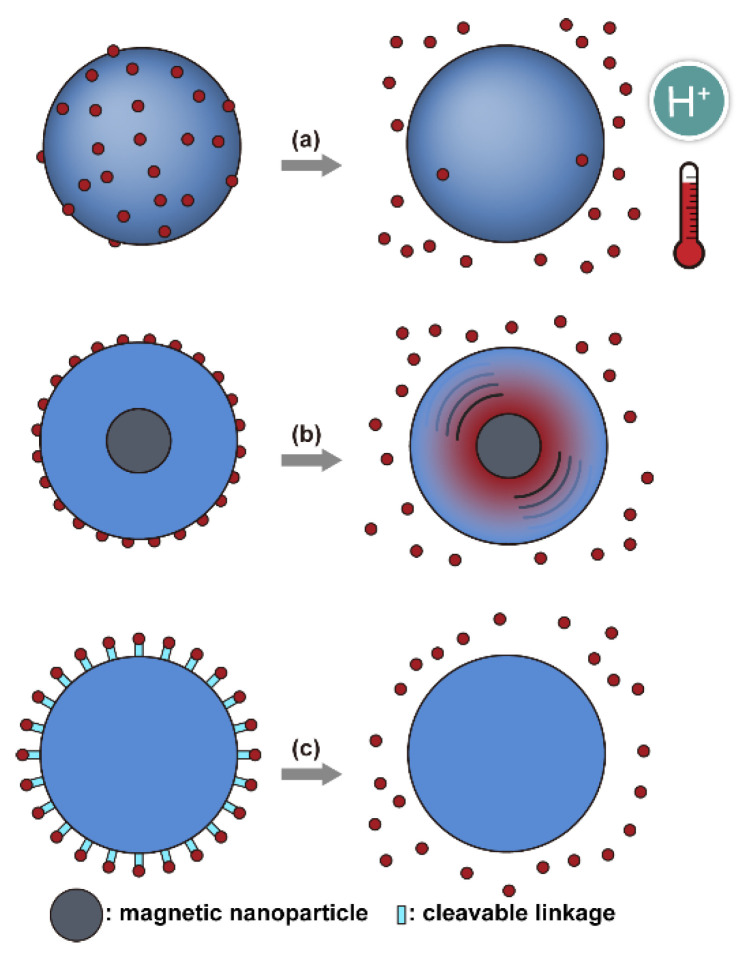
(**a**) Payload adsorbed to the surface of DDSs can be released when adsorption equilibrium shifts due to increased temperature or pH changes. (**b**) The release can also be stimulated by mechanical agitation via AMF. (**c**) For systems utilizing covalent bonding, release occurs after bond cleavage, for example, hydrolysis, enzymatic cleavage, and redox reactions.

In some systems, the drug is not encapsulated by but is attached to the nanoparticles, typically to the surface, via noncovalent interactions or covalent linkages (Figure 5). In the systems based on noncovalent interactions, i.e., adsorption systems, the payload is released as the adsorption equilibrium is shifted by stimuli such as protonation [237] or temperature elevation [238] (Figure 5a). Mechanical disruption has also been used to drive desorption (Figure 5b) [222]. Payloads attached by covalent links are released with the bonds are broken by stimuli such as pH [26,239], redox reactions [62,240,241], enzymatic attack [242,243], and photoirradiation [244] (Figure 5c). Systems based on covalent bonding have the potential to release a high fraction of the payload at the target site because they tend to avoid premature release [62,166,245,246]. However, the design of DDSs based on covalent bonding introduces the additional requirement that complementary conjugatable groups be present on the payload and the DDS [7].

Therapeutic agents have been loaded onto inorganic nanoparticles, such as noble metals [247,248,249,250], carbon-based nanomaterials [251], black phosphorus [237,252], and boron nanosheets [253], via noncovalent interactions, and similar noncovalent approaches have been evaluated for organic systems such as dendrimers [254,255,256] and polydopamine nanoparticles [238,257,258]. One major issue with DDSs based on adsorption is that attaching the drug via noncovalent interactions leaves the drug exposed to the physiological environment and susceptible to degradation or premature release, particularly in the case of solid nanoparticles where loading only occurs on the outer surface.

Covalently bonding reduces premature release and has been used to attach payloads to the surface of organic nanoparticles such as dendrimers [251,259,260,261] and polymeric nanoparticles [262,263]. Covalent bonding has also been used to prepare prodrug conjugates bonding therapeutic agents to free polymer or proteins [244,264,265,266,267]. The small size of these prodrugs, typically less than 10 nm in diameter, endows them with a long circulation half-life and enhances their extravasation via the EPR effect (Section 2.6.1) [7]. However, the drug remains exposed to the physiological environment where it is subject to degradation. Conjugating the payload to polymer chains in a nanogel [166,246,268,269] or micelles [62,240,241,270,271,272] reduces premature release and protects the payloads from physiological environments.

### 2.6. Targeting and Physiochemical Properties

DDSs for cancer therapy should accumulate at the tumor site or within cancer cells. Any fraction of the administered dosage that does not do so reduces the therapeutic efficacy and increases the potential for systemic toxicity. Therefore, strategies to maximize accumulation and cellular uptake of the nanocarriers at the treatment site and minimize this behavior elsewhere in the body are critical. These strategies can be generally classified as (1) passive targeting, (2) ligand-based targeting, and (3) stimuli-responsive targeting.

Both passive and ligand-based targeting are influenced by the interplay between the DDS and the physiological environment. Table 1 provides references for recent attempts to enhance passive targeting performance by optimizing these interactions. After entering blood plasma, the interactions between the nanocarriers and the components of the physiological environment can be significantly impacted by particle size, shape, stiffness, and surface properties. These factors affect the circulation time, clearance rate, the degree of tumor accumulation, and cell internalization processes. While the subject of this review is stimuli-responsive DDSs, an understanding of how these physicochemical properties affect targeting is essential to interpreting and comparing published research reports on stimuli-responsive DDSs. In this section, we briefly introduce passive and ligand targeting and discuss the effects of particle size, shape, surface properties, and stiffness on their efficacy. Stimuli-responsive targeting is reviewed in Section 3.8.

**Table 1:** Studies on critical characteristics of passive and active targeting strategies.

Targeting Method	Optimization Method	References
Passive targeting optimization	Nanocarrier size	[273,274]
	Protective polymeric layer (density and polymer length)	[275,276]
	Novel non-PEG protective layer	[117,277,278,279]
	Reduction in interstitial fluid pressure	[280,281]
	Degradation of physical barrier in ECM	[281,282,283]
	Normalization of tumor ECM	[284]
Active ligand targeting	Optimization of ligand-receptor interaction	[285,286]
	Ligand density	[287,288,289,290,291,292,293,294,295,296]
	Ligand orientation	[297]
	Ligand clustering	[298]
	Tether of ligands	[287,296,299]

#### 2.6.1. Passive Targeting

**Figure 6:**
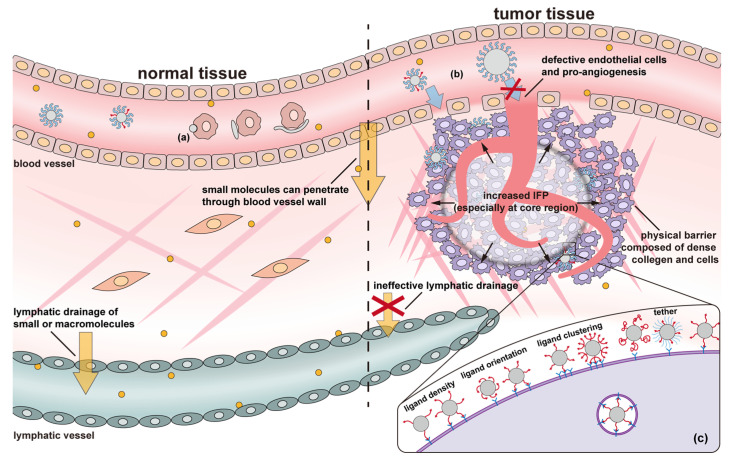
Schematic illustrating critical differences between normal and tumor tissues, lymphatic drainage of small molecules is reduced in tumor tissues, tumor vasculature has different porosity due to defective endothelial cells and pro-angiogenesis, tumors typically have an increased interstitial fluid pressure, and the dense collagen and cellular network forms a physical barrier within the tumor. Physicochemical factors affecting targeting include size, shape, flexibility, and surface properties. (**a**) Nanoparticles without proper stealthy decoration may be rapidly recognized and cleared through phagocytosis, while anisotropic or flexible nanoparticles are more likely to avoid phagocytosis. (**b**) Nanoparticles that are too large cannot enter the tumor from the vasculature despite its enhanced permeability. (**c**) Ligand density, orientation, clustering, and tethers all affect ligand-mediated endocytosis.

Tumor tissue is substantially different from normal tissues (Figure 6), where the endothelial cells are orderly and compactly arranged. In contrast, the microvasculature in tumor tissue contains enlarged endothelial gaps, and the membrane separating the vessels from the tumor is discontinuous [300]. This morphology results in numerous “openings” in the vessel walls of tumors, rendering them leaky. In typical vasculature, the gaps in the endothelial cells are 5–10 nm, while those in tumor vasculature are typically 100–780 nm, enabling more oxygen and nutrients to reach the tumor, resulting in rapid growth [7,301]. On the other hand, the dysfunction of the lymphatic drainage system reduces the clearance of therapeutics from the tumor tissue [302]. This combination of enhanced extravasation from the capillaries and reduced clearance via the lymphatic system results in the accumulation of macromolecules and nanoparticles within the tumor, known as the enhanced permeability and retention (EPR) effect [303,304]. Passive targeting makes use of this effect by carefully controlling the dimension of the DDS. If it is too small, the DDS can be rapidly cleared through the kidney. If it is too large, it is difficult for the DDS to extravasate into the tumor tissue. It is generally accepted that 30–200 nm in diameter is the appropriate dimension for DDSs to exploit the EPR effect [23]. However, the heterogeneity of tumors means that the proper DDS size may vary a lot between patients and tumor types [273,305,306]; large differences are seen even within a single tumor [307]. Even a 10 nm difference from the optimal size can result in a distinct reduction in internalization efficiency [274]. Therefore, determining the size required to optimize therapeutic efficacy in an individual patient is critical and requires a trial-and-error approach. Another issue is that whether the EPR effect exists and can be utilized on metastatic tumors is in dispute [308,309,310]. Finally, it is essential to note that non-cancerous pathological sites such as those associated with inflammations can exhibit the EPR effect as well. The accumulation of DDSs carrying antineoplastic compounds in those tissues is undesired [311,312].

While the EPR effect is enabled by the unique structure of vasculature in tumors, other characteristics of tumor tissue counteract this approach. Most significant is that many tumors have an increased interstitial fluid pressure (IFP), impeding mass transport deeper into the tumor [313]. In fact, the IFP in some tumors is sufficient to push cancer cells and growth factors out into surrounding tissue, facilitating tumor progression [314]. Similarly, IFP can reduce the amount of drug delivered to the target cells and may increase systemic toxicity by pushing therapeutics into surrounding tissues [315,316]. Often, the pressure gradient rises as the tumor core is approached, making deep tumor penetration difficult [23,313]. Other anatomical and physiological barriers, composed of several cell layers and a dense extracellular matrix (ECM), limit the ability of DDS particles to reach some cancer cells in the tumor [314]. To alleviate these issues, therapeutic agents, such as nitric oxide, histamine, TNF-α, vascular endothelial permeability factor (VEGF), that are capable of lowering IFP, improving vascular permeability, and enhancing extravasation can be included in the DDS formulation [280,281,307,308,317]. Additionally, tumor ECM can be degraded by hyaluronidase, enhancing the ability of DDSs to reach the cancer cells [281]. Finally, a combination of TGF-β inhibitor, ECM degradation enzymes, and the hormone relaxin can be used to reduce fibrosis and normalize ECM [282,283,318]. While a full discussion is beyond the scope of this review, Attia et al. and Narum et al. prepared excellent reviews of these techniques [319,320].

#### 2.6.2. Active Ligand Targeting

Advances in molecular biology revealed multiple ligands that are capable of binding to receptors that are overexpressed on cancer cells or by the periphery endothelial cells bounding the tumor [321,322]. Examples of ligands include large mono antibodies [244], small molecules such as folate and SV119 [207,323], glycoproteins that can induce receptor-mediated endocytosis [324], oligosaccharides [175], peptides [208], and nucleic acid aptamers [297]. Extensive targeting studies have revealed a myriad of targeting ligands for the delivery of therapeutics to heterogeneous tumor types [23]. The binding between these targeting ligands and the corresponding receptors is via noncovalent interactions, such as electrostatic forces, hydrogen bonds, and Van der Waals forces. Multiple studies have demonstrated the ability of target ligands decorated on a DDS surface to enhance cellular uptake and tumor accumulation [52,180,212,325,326,327,328,329,330,331]. The strong affinity between ligands and receptors can promote the accumulation of nanocarriers in tumors, essentially enhancing the EPR effect [33,332,333]. Second, ligand-targeted binding can facilitate cellular internalization of DDSs, improving the therapeutic efficiency of these systems [334,335]. Similar techniques can be used to target angiogenic endothelial cells to destroy the tumor vasculature and deprive the tumor of oxygen and nutrients. Chase et al. presented an in-depth overview of this strategy [336].

A few factors are critical in promoting cellular uptake via ligand binding (Figure 6c), e.g., the strength of the binding interactions, the density of the ligand coverage on the particle surface, and the orientation and distribution of the ligands on the DDS surface. A brief synopsis of the typical cellular internalization process is provided here to elucidate these effects. According to Nel et al., ligand-mediated uptake of nanoparticles initiates with the binding of ligands on nanoparticles that diffuse close to the cell surface with their corresponding receptors on the cell membrane [337]. Next, more receptors migrate to the connected region and bind with more ligands. The cell membrane then wraps the nanoparticle and internalizes it. During the process, specific ligand-receptor binding affinity, non-specific nanoparticle-cell membrane affinity, and the formation of a clathrin coat on the developing endosome promote nanoparticle wrapping. The entropic cost of receptor migration to the nanoparticle, cell membrane bending, and diffusion of the nanoparticle away from the cell downregulates endocytosis. Strong binding affinity minimizes the net energy costs of the process, increasing endocytosis. The choice in how the ligand is attached to the DDS also affects performance. According to Wang et al.’s simulation, the compression and stretching of ligand tethers lead to entropy loss, which increases the required energy for internalization [287]. The binding site of the ligand should be readily accessible, i.e., face outwards from the particle surface and not be sterically hindered by other surface groups [295,297,299]. Ligand density is also critical; in general, higher density promotes endocytosis [288,289]. However, high density can result in multiple ligands competing for each binding site, reducing overall binding strength [291]. Additionally, once a ligand has bound with a receptor, ligands from other nanoparticles are prevented from binding with that receptor. Thus, high ligand-density may over-recruit receptors, maximizing the endocytosis of one particle but overall diminishing the cellular internalization of the DDS [291]. When administrated in vivo, the clearance effect of ligand density should also be considered, i.e., the high density of target ligands on a surface may diminish the density of a stealth component, such as PEG, therefore increasing clearance by the mononuclear phagocyte system (MPS) [288]. Finally, some receptors are expressed on cancer cells in clustered patterns [338,339,340,341], and similarly clustered ligands enhance nanoparticle-cell binding [298]. Therefore, ideal ligand decoration should have a relatively high density, proper orientation and distribution, and be attached using an appropriate length of tethers.

Although a tremendous amount of work has been conducted to optimize target ligands, the improvement in the efficacy of these systems has been relatively modest [342,343]. This strategy works best when the DDS is effectively transported to and accumulates in the tumor near the cancer cells, thus requiring a limited clearance and effective utilization of the EPR effect [342,344]. It is difficult to meet all the required targeting goals simultaneously, i.e., stealth layers may enhance circulation time but limit uptake by cancer cells. This situation suggests the development of responsive targeting systems that enable DDSs to manifest setting appropriate characteristics that reduce clearance by the MPS when in the circulatory system and enhance uptake by the cancer cells from the tumor ECM.

#### 2.6.3. Related Physicochemical Properties

*Size*: A long circulation time allows individual DDS nanoparticles to have multiple chances to exit the vasculature at the tumor site. Thus, techniques to enhance circulation time can improve EPR-based targeting. The clearance of foreign bodies from the bloodstream primarily occurs in the kidneys, the spleen, the liver, and the MPS. Filtering mechanisms in the kidneys, spleen, and liver primarily, but not exclusively, remove foreign bodies based on size. The normal renal function typically clears particles smaller than ~6 nm. For nanocarriers in the 6–8 nm range, Longmire et al. reported that clearance depends on both size and surface charge [345]. Thus, to avoid clearance by the kidney, nanocarriers should have a hydrodynamic diameter above 10 nm. However, the nanocarrier dimensions cannot be too large due to the significant physical filtration capacity of the spleen and liver. The tight reticular mesh in the spleen can trap nanocarriers larger than 200 nm, and the liver can effectively capture particles larger than 150 nm [346,347]. Nanoparticles trapped and degraded by hepatocytes are eliminated via biliary excretion; however, this process is slow compared to other clearance mechanisms, and buildup of nanomaterials in the liver is a concern [7]. In addition to the clearance by the kidney, spleen, and liver, the mononuclear phagocyte system (MPS), components of which are broadly distributed via the circulatory system and highly concentrated in the liver and spleen, can effectively clear foreign nanoparticles as small as 10 nm [345]. While particles of 10–150 nm can avoid clearance by the kidney, spleen, and liver, additional strategies are needed to reduce recognition and clearance by the MPS.

Size can also significantly impact cellular uptake. Regardless of the binding mechanism, non-specific or ligand-mediated, the cytoplasmic membrane invaginates to engulf the nanoparticle forming an endosome, which then transports the payload to lysosomes for degradation [348]. It is important to understand that endocytosis is size-dependent. A threshold of particle size exists, ~5 nm, below which endocytosis does occur [349]. The optimal size seems to be in the range of 40 to 60 nm for both inorganic and polymeric nanoparticles [350,351,352,353,354,355]. When the size is larger than the optimal size, the endocytosis efficiency is reduced gradually [356]. These effects are related to the energy requirement for membrane bending and the free energy released by the particle adsorbing or binding to the surface. Enhancing the affinity of nanocarriers to the cell membrane increases the free energy from adsorption, promoting endocytosis, and reducing the threshold size [356]. However, it should be noted that nanoparticle aggregation may increase the effective size of nanoparticles; the clusters act as a single larger particle, which may enhance the cellular uptake of particles smaller than the endocytosis size threshold and reduce cellular uptake for larger particles [357].

*Surface Properties*: DDS surface properties, such as charge and hydrophobicity, also impact circulation time by mediating interactions with the MPS system. Generally, the cell membranes are negatively charged. Thus, a higher endocytosis efficiency is expected from positively charged nanoparticles, and one would expect that a negatively charged surface would enhance circulation time. However, while this trend is observed in carefully controlled experiments [358,359,360,361], a noticeable impact is not observed in in vivo tests [362]. The reason for this behavior is that both positively and negatively charged particles attract proteins to the surface that activate the MPS. Hydrophobic surfaces can also enhance the formation of a MPS activating protein corona [363,364,365]. As a result, a neutral or weakly negatively charged hydrophilic surface is favorable for long-term circulation. PEGylation of outer surfaces is the most used mechanism to enhance circulation time. Knop et al. prepared a systematic review of the application of PEG to nanoparticles [366]. They concluded that a short polymer chain (1–5 kDa) and a high surface density are most effective at extending circulation time. However, a significant immune-response can be initiated if a large dose of PEG-coated nanocarriers is used or after repeated dosing [366]. Other drawbacks to the use of PEG coatings to enhance circulation time include poor degradability and the formation of toxic side-products during PEG synthesis and grafting [276,366,367,368]. Additionally, after nanocarriers enter tumors, the hydrophilic PEG-rich surface may block the cellular uptake by cancer cells. The release of payloads in the tumor extracellular matrix may not only reduce therapeutic efficacy but also result in their return to the blood vessels, via the increased IFP, potentially inducing systemic toxicity [315]. Though alternative synthetic polymers have been explored to replace PEG, most of them do not exhibit equivalent performance nor have in-depth biocompatibility or long-term safety studies of them been performed [366]. In contrast, bio-inspired natural materials, i.e., polysaccharides, albumin, and red blood cell membrane, can not only extend circulation life, but also mediate selective cancer cell uptake of DDSs [277,278,369,370].

The effects of surface roughness on cellular internalization are not apparent. Schrade et al. attached 12 nm silica nanoparticles on polystyrene (PS)-based nanoparticles to fabricate rough nanoparticles and reported that uptake by HeLa cells was more significant for the smoother nanoparticles regardless of surface charge [371]. Piloni et al. reported greater uptake by murine macrophages, breast cancer cells, and fibroblasts of smooth nanoparticles than of 150 nm micelles with 20 nm-radius protrusions [372]. In contrast, Niu et al. found that the attachment of 20 nm silica particles onto smooth 230 nm silica nanoparticles enhanced the uptake by breast adenocarcinoma and squamous carcinoma cells regardless of whether the particles were hydrophilic or hydrophobically surface modified. They attributed this to the enhanced protein aggregation in the void spaces between protrusions. Verma et al. explored this behavior by preparing “rough” surfaces on gold nanoparticles [373]. They reported that nanoparticles with randomly distributed “pits” exhibited moderately enhanced uptake by mouse dendritic cells versus smooth nanoparticles. However, nanoparticles with a striated surface, prepared with alternating regions of anionic and hydrophobic groups, exhibited the highest uptake rate. Overall, the effect of surface roughness on cellular uptake is still in dispute. Part of the reason may be the different experimental conditions, material compositions, and size ranges explored.

*Shape*: Shape also influences cellular uptake, circulation time, and EPR-based accumulation in tumor sites. High aspect ratio (AR) particles exhibit different cellular uptake than isotropic particles. However, the results on cellular uptake seem contradictory: some studies indicate higher uptake for high AR nanoparticles particles [245,374,375], while others suggest a higher uptake for spherical particles [376,377]. Other nonspherical particles such as nanodiscs and nanocubes, though not as extensively, have also been explored for drug delivery. Endocytosis simulations of these shapes show that the internalization efficiency was reduced as the energy required by the cytoplasmic membrane for nanoparticle wrapping increased [378]. Wrapping a nanodisk has the highest energy cost, followed by nanorods, nanocubes, and nanospheres. Some experimental observations agree with simulations and indicate that nonspherical shapes exhibited lower endocytosis rates [351,379,380]. However, in other studies, nonspherical nanoparticles exhibit enhanced endocytosis [381,382]. Mitragotri et al. suggested that internalization initiation depends on the local dimension of the nanoparticle in contact with the cells [383,384]. Generally, the smaller the local dimension, the more likely the process is to be initiated. They evaluated several geometries and found significantly lower rates of phagocytosis for wormlike nanoparticles of polystyrene than for nanospheres [377]. Both particles had the same volume, but the aspect ratio of the wormlike particles was greater than 20. Geng et al. found that wormlike micelles also exhibited a prolonged circulation time of up to 5 days [385]. They attributed these results to macrophage capture being overcome by the large hydrodynamic forces these particles experience. They also noted that this long circulation life was reduced when they added crosslinks, suggesting that stiffness is a factor in the behavior. For cells in tumors, where hydrodynamic forces are minimized compared to the circulatory system, several studies have reported lower internalization for high AR nanoparticles [386,387,388]. Interestingly, the opposite result has been reported for particles decorated with target ligands, in which case high AR nanoparticles exhibit a higher internalization that their spherical cousins [375,389,390]. This contrast is attributed to more ligands available on high AR nanoparticles, which allows multivalent binding of nanoparticles to cells. Clearly, more work is needed to address the effect of DDS anisotropy on cellular uptake and circulation life. The effect of AR on tumor site accumulation via the EPR effect seems clearer. Several in vivo studies have demonstrated higher accumulation in tumors and reduced liver capture for high AR nanoparticles [376,391,392]. On the other hand, high AR nanoparticles seem to enhance accumulation in the lung and spleen [245,393].

*Stiffness*: As a flexible, “soft” nanoparticle interacts with a cell membrane, it can spread on the membrane, enabling more binding at the interface. This effect suggests that low-modulus nanoparticles promote the initiation of endocytosis and that the threshold size for endocytosis is also reduced. On the other hand, as the cell membrane enwraps the nanoparticle, “soft” nanoparticles can deform into cone-like shapes with a relatively sharp tip and curved cap, followed by an ellipsoidal shape oriented perpendicular to the cell membrane. These deformations require additional energy and deformation of the cell membrane to wrap the nanoparticle and complete endocytosis. As a result, softer nanoparticles undergo slower endocytosis than stiffer ones. Both effects were seen in the simulations developed by Yi et al. [394,395] and Shen et al. [396], where the endocytosis of flexible nanoparticles began earlier but was completed later than the endocytosis of more rigid particles. Shen et al.’s simulation also suggested that the smaller the nanoparticle and the lower the affinity between the nanoparticle and cell membrane, the more significant the effect that the stiffness exhibits during endocytosis. Experimental studies are somewhat contradictory, with some reporting faster endocytosis for softer nanoparticles [397,398,399]. In contrast, others suggest that stiffer particles are more readily internalized [400], and Banquy et al. reported that the nanoparticles with an intermediate Young’s modules exhibited the highest uptake efficiency [401]. It should be noted that the stiffness ranges tested in these studies are different, and there is evidence that other properties modify the effect of stiffness on cellular uptake. Banquy et al. suggest that the effects of stiffness and size are interrelated, with larger particles exhibiting a smaller stiffness effect than smaller particles [401]. The interrelationship between shape and stiffness on endocytosis of different cells was explored by Alexander et al. [382]. They found cellular uptake to be highly dependent on flow conditions, shape, rigidity, and cell lines. They also demonstrated that clearance via the MPS was reduced for soft nanoparticles. Soft nanoparticles can also more easily penetrate capillary walls during circulation [382,402], suggesting that soft DDSs could better take advantage of the EPR effect. Enhanced tumor accumulation and reduced clearance by the MPS system of soft DDSs have also been demonstrated in vivo [403,404,405].

## 3. Stimuli

The tumor physiology can be significantly different from that of normal tissue. Uncontrollable cell proliferation, poor blood perfusion, and altered cellular metabolism generate an acidic hypoxic microenvironment [406,407]. The pH in the tumor ECM can be as low as 6.8 [408,409,410]. It drops further, to 6.0–6.5 in the endosome, to 5.0–5.5 in late endosomes, and 4.5–5.0 in lysosomes [411,412]. The reduction–oxidation (redox) balance is also altered in cancer cells. Due to the abnormal cellular activity and rapid propagation, the concentration of reactive oxygen species (ROS), primarily generated through mitochondrial metabolism, is typically elevated in cancer cells [413,414,415,416]. Reductive agents such as glutathione (GSH) are usually overexpressed in cancer cells to relieve the high oxidative pressure that results from elevated mitochondrial metabolism [416,417,418]. As a result, GSH levels are elevated in inflammations, neurological diseases, and tumors, and can be several orders of magnitude higher in cancer cells than the ECM [419,420,421,422,423,424,425,426,427,428,429,430,431]. Similarly, several enzymes, including hydrolases, proteases, and oxidoreductases, are overexpressed in tumors [124,432,433,434,435,436,437,438]. Finally, many inflammatory disease processes lead to hyperthermia, and temperature elevation is observed in tumors [439,440]. All of these unique properties of tumors and cancer cells can be considered endogenous stimuli and have been exploited to stimulate the release of therapeutic agents from DDSs. Exogenous stimuli such as photoirradiation, ultrasound, oscillating magnetic fields, and locally induced hyperthermia have also been exploited to activate the release of payloads from DDSs. In this section, recent advances in the application of each of these stimuli to enhance efficacy in DDSs are reviewed.

### 3.1. pH-Responsive DDSs

As summarized in Table 2, there are four primary responses to elevated pH that have been used to initiate drug release from DDSs (1) hydrolysis of acid-labile bonds, (2) cleavage of metal-ligand coordinate bonds, (3) protonation of groups that affect noncovalent interactions, and (4) degradation of acid-labile inorganic materials. Uniquely for liposomes, reduced pH-induced membrane fusion can also result in payload release. Hydrolysis is accelerated in the presence of acid; thus, the lower pH in the ECM of tumors and cancer cell endosome/lysosomes can serve as a stimulus to induce drug release. The hydrolysis of dimethylmaleic amide [40,173,441], orthoester [442], boronic ester [27,202,272], oxime [41], benzoic imine [180,203,239,264], ketal/acetal [42,155,266,443], and hydrazone [26,270,271] have all been studied. Chen et al. demonstrated that hydrolysis reactions are affected by neighboring groups, suggesting a method to tailor the pH sensitivity of DDSs exploiting pH triggering via hydrolysis [444]. Ordinary amide and ester bonds undergo slow hydrolysis in the weakly acidic conditions of the endosome and lysosome, making them unsuitable for stimulated release systems. To overcome this issue, novel compounds have been explored, such as β-thiopropionate and hexahydrobenzoic amide, which form stabilized intermediate structures, reducing the reaction barrier and facilitating hydrolysis [241,445]. Some metal-ligand coordinate bonds are disrupted at low pH due to the competition between protons and metal. For example, the Fe^3+^-catechol complex changes from a tri-catechol to a mono-catechol complex as pH drops [446,447,448]. This effect is frequently used in MOFs as the reduced coordination number disrupts the MOF structure and releases the payload.

**Table 2:** Summary of pH-induced response mechanisms used in stimuli-responsive drug delivery systems.

Mechanism	Chemistry	References
Covalent bond cleavage	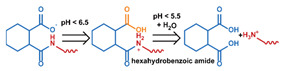	[445] ^a,b,c^
	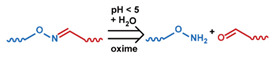	[41] ^b,c,^**
	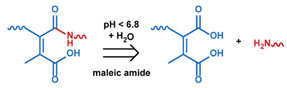	[40] ^b,c,^*** [173] ^b,d^ [441] ^a,b,c,^***
	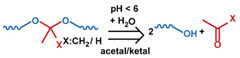	[42] ^b,c,^* [155] ^b,d,^*** [156] ^b,c,^** [443] [449] ^b,c,^**
	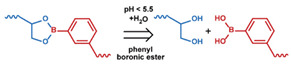	[27] ^b,c^_,_* [202] ^b,c,^*** [272] ^c,^** [325],^a,b,c,^*** [450] ^a,b,c,^***
	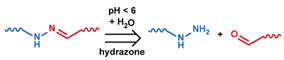	[26] ^b,c,^** [270] ^b,c,^*** [451] ^b,c,^***
	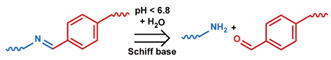	[166] ^b,c,^** [203] ^a,b,c,^*** [264] ^b,c,^** [333] ^b,c,^** [452] ^a,b,c,^*
	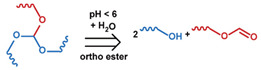	[119] ^b,d,^* [442] ^a,b,d^ [453] ^b,c,^***
	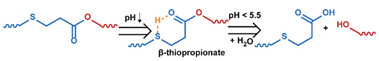	[241] ^a,b,c,^***
	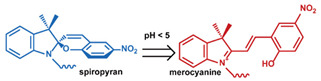	[454] ^b,c,^** [455] ^b,c,^***
Coordination bond cleavage	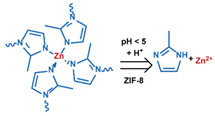	[223] ^a,b,c,^** [224] ^c^ [225] ^b,c,^*** [236] ^a,b^ [456] ^a,b,c,^***
Inorganic chemical degradation	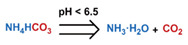	[179] ^a,b,d,^*** [457] ^c,^** [458] ^b,c,^**
	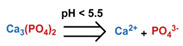	[459] ^b,c,^*** [460] ^c,^*
	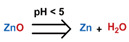	[461] ^b,c,^***
Membrane fusion	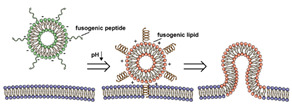	[138] ^b,c^ (peptide)
		[119] ^b,d,^* [123] ^b,c,^*** [442] ^a,b,d^ [452] ^a,b,c,^* [462] ^a,b,c^ (lipid)
Protonation	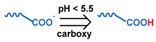	[70] ^b,c,^*** [71] ^b,c,^*** [121,122], [181] ^c,^*** [462] ^a,b,c^ [463] ^b,c,^* [464] ^c,^** [465] ^c,^*
	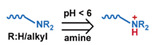	[51] ^a,b,c,^** [123] ^b,c,^*** [237] ^a,b,c,^** [238] ^a,b,c,^** [462] ^a,b,c^ [464] ^c,^** [466] ^b,c,^*** [467] ^c,^*
	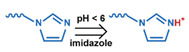	[50] ^a,b^ [206] ^b,c,^***
	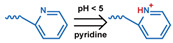	[468] ^a,b,c,^***
	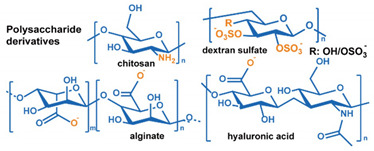	[157] ^a,b,c,^*** [166] ^b,c,^** [174] ^a,b,c,^* [175] ^a,b^ [469] ^b,c,^** [470] ^a,b,c,^***
	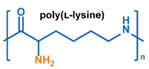	[65] ^b,c,^** [174] ^a,b,c,^* [175] ^a,b^

^a^ Evaluation included in vivo tests on mice.; ^b^ Evaluation included in vitro tests on cells.; ^c^ Small molecules, such as antitumor drugs and dyes, were used as payloads.; ^d^ Large molecules, such as proteins and plasmid DNA, were used as payloads.; * Premature payload leakage was >20%.; ** Premature payload leakage was between 10% and 20%.; *** Premature payload leakage was ≤10%.

Protonation can alter the surface charge, increase zeta-potential, and affect hydrophilicity, inducing destabilization of the nanocarrier and promoting payload diffusion due to weakened intermolecular attractive interactions [237,238,463]. Creating a positive charge via protonation can also be used to direct nanocarriers into mitochondria, enabling direct delivery of therapeutic agents to this organelle [125,445,471]. Typically, a functional group with a pKa in the range of 4.5–7.4 is used in pH-responsive DDSs that utilize protonation as the trigger. These include carboxylic acid [65,71,181], amine [51,254], imidazole [50,206], and polysaccharides such as hyaluronan [157,174,175] and chitosan [472,473]. Protonation can induce conformational changes in peptides that destabilize lipids, promoting payload release, or facilitate insertion into biological lipid membranes, enhancing endosomal escape and payload release into the cytosol [138,474], Acid-induced degradation of inorganics such as calcium phosphate (Ca_3_(PO_4_)_2_) shells or zinc oxide (ZnO) gatekeepers allow the encapsulated payloads to be released at a pH below 5.0 [459]. The CO_2_ pressure that results from the acid-induced degradation of NH_4_HCO_3_ has been demonstrated to be sufficient to crack polymeric shells, enhancing payload release [457]. Recent advances in the application of these techniques to the various platforms discussed in Section 2 are reviewed below.

#### 3.1.1. pH-Responsive Micelles

Acid-catalyzed hydrolysis has been used to endow polymeric micelle-based DDSs with pH responsiveness. Linkages that undergo acid-catalyzed hydrolysis have been (1) incorporated in the polymer backbone, typically in the hydrophobic block (Figure 1a), (2) used to connect the hydrophobic and hydrophilic blocks (Figure 1b), (3) used to attach pendant groups to the amphiphilic polymer (Figure 1d), and (4) used to form covalent crosslinks between amphiphilic molecules in the micellar structure (Figure 1c). Petrova et al. reported that the hydrolysis of a ketal linkage used to connect PEG and poly(caprolactone) (PCL) blocks increased as pH dropped and resulted in micelle destabilization and aggregation [443]. Jin et al. evaluated the pH-enhanced hydrolysis of oxime linkages, present in the PCL block backbone, to induce dissociation of PEG-PCL triblock copolymer micelles and release doxorubicin (DOX) [41]. While they reported that oxime linkage increased the fraction of drug released, they also found that these systems exhibited a distinct premature release behavior. To address issues with premature release, Zhang et al. used comb-like copolymers produced from a poly(ethylene glycol) methyl ether methacrylate macromonomer (mPEGMA) and 2,4,6-trimethoxybenzyli- dene-1,1,1-tris(hydroxymethyl) to prepare curcumin-loaded micelles [453]. The hydrophobic, 2,4,6-trimethoxybenzylidene pendants limited the premature release of the payload as compared to micelles produced without the pendant groups. However, under acidic conditions, the pendants were cleaved from the copolymer, and enhanced hydrophilicity of the micelle core led to micelle swelling and over 60% of the drug being released in 24 h. In another study, they conjugated dimethylmaleic acid (DMMA) to the hydrophobic block of a PEG-b-PCL copolymer via an amide bond and used the product to prepare DOX containing micelles [40]. The negatively charged carboxyl group on the DMMA formed a strong noncovalent bond with the positively charged DOX, and the loaded micelles exhibited a significantly lower rate of DOX leakage in neutral conditions than micelles without the DMMA pendant group. However, under acidic conditions, the amide bond was hydrolyzed, exposing a positively charged amine group and resulting in the rapid release of the payload. They also reported that the pH sensitivity of this system could be altered by changing the amount of DMMA present. The most sensitive system released 90% of the loaded DOX within 5 h under acidic conditions. Li et al. examined the pH-induced breakage of intermolecular crosslinks between hydrophobic segments in micellar structure as a stimulus for paclitaxel (PTX) release [450]. They used covalent boronate-crosslinks formed by boronic acid and the catechol groups present in a pair of cholic acid-PEG telodendrimers. Both compounds were present as pendant groups on the primary amphiphile. At pH 5, the boronate bond degraded, allowing the release of the payload.

Protonation can also be used to disrupt polymeric micelles by changing the strength of the noncovalent intermolecular interactions in the structure. Xu et al. prepared DOX-loaded supramolecular micelles based on hydrophobic poly(L-leucine) and hydrophilic dendritic poly(L-lysine) conjugated by the electrostatic interaction between amine and carboxyl groups in neutral pH [65]. Protonation of the carboxyl groups on poly(L-leucine) below pH 6.2 led to micelle disassembly and a rapid release of the payload, 50% in 3 h. In contrast, Fan et al. evaluated the dissociation of micelles upon the reduction in intermolecular hydrogen bonding between hydrophobic segments [466]. They used a copolymer that contained tertiary amine and adenine moieties on a pendant side chain in the hydrophobic block and synthesized a micelle that included uracil-based crosslinks. In their system, the deprotonated hydrophobic tertiary amine contributed to the stability of the micellar structure at neutral pH. However, in acidic conditions, protonation of the tertiary amine and the adenine groups enhanced hydrophilicity disrupting the hydrogen bonding based crosslinking, leading to dissociation of the micelles and release of the payload.

#### 3.1.2. pH-Responsive Liposomes

The insertion of acid-labile bonds in lipids can endow liposomes with pH-responsive behavior. Guo et al. explored how the location of acid-cleavable orthoesters present in lipids could be used to tailor the pH-responsiveness of liposomes used for the delivery of DNA [119,442]. They evaluated ortho esters as the linkage between hydrophobic tails and the hydrophilic head of liposomes (Type I) and at different locations along the hydrophobic tail of liposomes (Type II). Under acidic conditions of the late endosome/lysosome, the hydrolysis of the orthoester led to liposome destabilization, aggregation, and payload release. The Type I system exhibited both a higher pH sensitivity and lower cytotoxicity as compared to the Type II system [119]. Cleavage of the orthoester in Type II systems converted the lipid into a lysolipid, destabilizing the cellular membranes, inducing cytotoxicity, and diminishing transgene expression.

In contrast to bond breakage in the lipids, Chen et al. explored the use of hydrolysis to remove a protective PEG coating from liposomes [452]. In their work, PEG was conjugated to cholesterol present in the liposome via a benzoic-imine bond. Hydrolytic cleavage of the bond at pH 6 removed the protective PEG layer and exposed the fusogenic lipid DOPE. The payload was released by the DOPE-promoted fusion of the liposome with endosome or cell membranes.

Protonation under acidic conditions has also been explored as a mechanism to endow pH sensitivity to liposome DDSs. Terreno et al. used lipids with carboxyl groups on the hydrophilic lipid heads [121,122]. During the preparation of the DDS at neutral pH, the negative charge present stabilized the structure and prevented liposome aggregation. However, when the pH was reduced to 5, protonation neutralized the charge resulting in destabilization and aggregation of the liposomes and complete release of the payload, an MRI contrast agent TmHPDO_3_A. When nanocarriers are captured by cells via endocytosis, a pH of 5 is only reached when the vesicles encapsulating them transform to late endosomes/lysosomes. However, releasing the payload into these vesicles may result in degradation and efficacy reduction. An alternative approach for liposomes is to enhance membrane fusion so that the payload is released into the cytosol. In Obata et al.’s work, both carboxyl and amine groups were added to the hydrophilic liposome head [462]. In neutral conditions, the liposomes were stable and well preserved. However, as pH dropped to ~5, the protonation of both groups resulted in a weakly positive charge on the liposome surface. This charge reversal promoted fusion with endosomal membrane and drug release into the cytosol. Compared with the control liposome, which possessed a neutral zeta-potential at pH 4–7, significantly enhanced endosomal escape in HeLa cells under confocal laser scanning microscopy (CLSM) and greater cytotoxicity were observed for the pH-responsive liposome.

Protonation of fusogenic lipids and the incorporation of pH low insertion peptides (pHLIPs) can facilitate the fusion between liposomes and biological membranes. Under low pH conditions, a pHLIP undergoes conformational changes that result in its insertion into lipid bilayers [137]. Yao et al. evaluated liposomes that consisted of a blend of a pHLIP conjugated PEGylated lipid and the fusogenic lipid DOPE [138]. When exposed to the reduced pH conditions found in tumor ECM, the pHLIP undergoes conformational changes that result in its insertion into the cellular membrane or endosomal membrane [137]. This process pulls the liposome close to the membrane, promoting protonated DOPE-induced fusion, releasing the contents directly into the cytosol. In addition to pHLIP, other peptides and proteins that can interact with lipid membranes have been explored to facilitate membrane fusion; the readers are directed to the reviews by Le et al. and Sanderson for more details [475,476].

#### 3.1.3. pH-Responsive Polymeric Nanoparticles

Polymers with pH-labile linkages can be used to produce pH-sensitive polymeric nanoparticles. Hydrolysis of dimethyl ketal links in albumin containing polymeric nanoparticles was explored by Paramonov et al. [155]. They evaluated a series of systems with different degrees of hydrophobicity and found that processability, stability, and pH-sensitivity varied. The optimal system exhibited low premature release (<20%) at neutral pH and complete drug release in acidic conditions in 24 h. In contrast with the hydrolysis of main chain covalent bonds, Wei et al. used ketal both as crosslinks and to attach pendant groups on a nanogel-based DDSs [449]. Under acidic conditions, the partial cleavage of the crosslinks and the formation of hydroxy groups when the pendants were cleaved increased swelling of the nanogel, releasing the payload. In another technique, pH-induced cleavage causes a charge reversal, leading to the dissociation of polyelectrolyte-complexed nanoparticles. Lee et al. capped amine groups on the side chain of the polycation poly(2-[2-aminoethyl)amino]ethylaspatamide)) (pAsp(DET)) with cis-aconitic anhydride (Aco) via maleic amide bonds [173]. The resulting polyanion, pAsp(DET-Aco), underwent charge reversal when the amine groups were exposed via cleavage of the maleic amide bonds. Confocal laser scanning microscopy (CLSM) confirmed that the abundant positively charged amine groups exposed in the low pH environment of the endosomes promoted both dissociation of the nanoparticle and endosomal escape of the plasmid DNA (pDNA) payload into the cytosol.

Protonation weakens the attractive interactions in nanoparticles containing oppositely charged polyelectrolytes resulting in enhanced permeability of encapsulated payloads. Li et al. reported that the addition of chitosan-alginate shells to poly(methacrylic acid) (PMAA) nanogels reduced premature release in neutral conditions, but that a pH of 5, protonation of the DOX payload, amine groups, and carboxyl groups destabilized the structure and releasing 60% of the payload in 8 h [469]. In comparison, only 40% was released from PMAA nanogels without the chitosan-alginate shell. Oishi et al. evaluated the effect of protonation of a tertiary amine on the swelling of a PEG-based nanogel [176]. The extent of swelling and the required pH for protonation were impacted by the ratio of the amine-containing monomer, 2-(N,N-diethylamino)ethyl methacrylate, and the hydrophobic monomer, 2,2,2,-trifuoroethyl methacrylate (TFEMA), in the nanogel. Protonation has also been explored for mixed polyelectrolyte systems. Dreaden et al. examined a polymer nanoparticle DDS prepared via layer-by-layer coating of a fluorescent polystyrene particle with the polyelectrolytes hyaluronic acid (HA), pKa ~3, and poly(L-lysine), pKa ~10 [175]. Obvious swelling and a shift in the surface potential (from −15 mV to −3 mV) was observed at pH 7. A pH drop from 7.4 to 6.0 resulted in a 2.4-fold increase in cellular fluorescence during liver hepatocellular carcinoma (HepG2) cell assays, indicating the swollen structure and more hydrophobic surface promoted cell uptake. Protonation can also be used to enhance drug release from nanogels via charge state change and hydrophobicity modification. The protonation of carboxyl groups to reduce hydrophilicity was explored by Selinas et al. as a mechanism to induce the release of Nile blue (NB) from nanogels [464]. At neutral pH and room temperature, the loaded nanogels were stable and did not exhibit significant leakage of NB over 24 h. However, protonation of the carboxyl groups when the system was incubated at a pH of 5.2 weakened intermolecular interactions between the polymer and the NB, leading to over 35% of the payload being released in 24 h. The system also exhibited dual responsive behavior. Heating the system to 43 ℃ resulted in the release of over 80% of the payload in 24 h under tumor ECM conditions and in less than 10 h at a pH of 5.2. Thermo-responsive mechanisms are reviewed in Section 3.4. Lee et al. used the increased hydrophobicity of protonated carboxyl groups on poly(acrylic acid) (PAA) to develop a pH-responsive polymer-caged liposome system [465]. They inserted cholesterol-terminated PAA in the lipid membrane and crosslinked the PAA to form a nanogel shell, effectively suppressing payload leakage under neutral conditions. However, at low pH, protonation of the carboxyl groups perturbed the lipid membrane. The increased hydrophobicity of the PAA and resulting shrinkage resulted in compression of the liposomes, and the PAA also inserted into the bilayer. Both effects destabilized the lipid membrane enhancing permeability and releasing the payload, calcein.

A unique application of pH-responsive behavior is the rupturing of polymeric nanoparticle shells by CO_2_ gas generated from NH_4_HCO_3_ decomposition. In Liu et al.’s work, antigen and NH_4_HCO_3_ were co-encapsulated in a PLGA [179]. In acidic media, protons diffusing into the DDS induced degradation of NH_4_HCO_3_. Sufficient CO_2_ was generated to rupture the PLGA shell and destabilize the endosome membrane. These “nanobombs” enhanced the release of the antigen from the endosome and induced an immune response.

#### 3.1.4. pH-Responsive Porous Inorganic Nanoparticles

Gatekeepers immobilized via acid-labile bonds have been used to impart MSN-based DDSs with pH responsiveness. Gan et al. attached Fe_3_O_4_ nanoparticles on MSNs via boronate esters, which endowed both magnetic and pH responsiveness to the nanoparticles [202]. In a neutral environment, leakage of the dexamethasone payload was entirely eliminated by the presence of the gatekeeper. The release of the gatekeeper when the ester bond was cleaved resulted in the release of the payload. However, this did not occur until the pH was reduced to 3, significantly below the typical pH, even in lysosomes. In comparison, Zeng et al. demonstrated significant release at a pH more representative of tumor ECM and endosomes for an MSN using benzoic-imine bonded gatekeepers [203]. The amount of payload released was less than 10% at pH 7.4, while over half the drug was released at pH 6.8, and over 80% was released at pH 4.5.

Protonation can also be used to remove gatekeepers in MSN- and MOF-based DDSs. In Meng et al.’s work, protonation of benzimidazole present on the surface of MSN weakened its affinity to β-cyclodextrin (β-CD) gatekeeper [206]. This unblocked the pores leading to the release of the DOX payload. In Deng et al.’s work, MSN pores were blocked by chitosan under neutral pH conditions preventing the premature release of tumor necrosis factor α (TNF-α) [470]. Protonation of the chitosan converted it from a globule to a random coil state unblocking the pores. Tan et al. used carboxylatopillar[5]arene (CP5) as a gatekeeper on a series of MOFs [71,181]. Under acidic conditions, protonation of CP5 weakened its affinity to the tertiary amine and pyridinium units present on the MOFs, resulting in the release of both CP5 and the cargo, 5-fluorouracil (5-Fu) or rhodamine 6G (Rh6G). However, a relatively strong acidic condition (pH 4) was required to achieve significant drug release.

Degradation of acid-labile inorganic compounds used in encapsulating shells can also be used to impart pH responsiveness to MSN- and MOF-based DDSs (Figure 4c,g). In Chen et al.’s study, a CaP-HA biomineralization shell was fabricated on the MSN surface to prevent leakage of encapsulated rhodamine B (RhB) [459]. CaP was degraded at pH 4.5, releasing the encapsulated RhB. At the same time, the HA conferred CD44 targeting to the DDS. In cellular assays, high cytotoxicity of DOX-loaded MSN was observed in breast cancer (MDA-MB-231) cells. In contrast, cytotoxicity of normal fibroblast (NIH3T3) cells was low even at a high DOX concentration, 12 μg/mL. Organic shells have also been explored, such as the glucose membrane used in Zhang et al.’s study [325]. An acid-labile boronate ester was formed between glucose and the boronated ligand of the MOF, resulting in a protective glucose shell. The shell not only eliminated drug leakage in a neutral medium but also acted as a targeting strategy due to the overexpression of the glucose-transport protein in tumors. The in vivo test revealed that HeLa tumor growth was suppressed by the DOX-loaded DDS. Notably, the metal residuals of the MOF were excreted within several days after administration, indicating the existence of an efficient excretion pathway and reducing concerns about long-term toxicity.

DDSs based on MSN and MOF that respond to reduced pH conditions can also be fabricated by incorporating pH-sensitive compounds within the structure. He et al. produced an MSN-based DDS capable of rapid degradation when the pH was less than 5.5 by incorporating Ca^2+^ and PO_4_^3−^ in the silica matrix during MSN synthesis [460]. The system exhibited an enhanced release of DOX versus the control MSN without the Ca^2+^ and PO_4_^3−^ in the structure at pH 5.5. However, the MSN possessed a mesoporous structure, and leakage of ~20% of the payload was observed in 10 h in neutral conditions, relatively high among MSN nanocarriers. In MOFs, protonation of organic ligands can weaken their affinity with metal cations, leading to MOF degradation. Wang et al. encapsulated dihydroartemisinin (DHA) in the Materials Institute Lavoisier (MIL) framework composed of Fe(III) ions and benzene-1,3,5-tricarboxylic (BTC) ligands [236]. Degradation of the MIL in endosomes/lysosomes released Fe(III) and DHA. The intracellular reduction of Fe(III) to Fe(II) ions promoted the generation of ROS from DHA, enhancing the cytotoxicity of the system. However, this effect was not evident during the in vitro test, probably due to incomplete degradation of MOF. The in vivo test of this system in tumor-bearing mice revealed significant inhibition of tumor growth with a slight impact on body weights. Furthermore, a Fe_3_O_4_-carbon nanocluster was encapsulated in the MOF, endowing it with magnetic field guided targeting, increased magnetic resonance imaging (MRI) contrast, and fluorescence imaging properties. In addition to the MIL system, other MOFs exhibit acid-catalyzed degradation. A comprehensive summary was conducted in Wang et al.’s review [477].

#### 3.1.5. pH-Responsive Non-Encapsulated DDDs

For systems in which the payload is bound to the nanocarrier via noncovalent interactions, reduced pH can initiate release either by weakening the drug-carrier intermolecular attraction, by increasing drug solubility in the surrounding medium, or by enhancing ion exchange. In Wang et al.’s work, DOX and 7-ethyl-10-hydroxycamptothecin (SN38) were adsorbed onto polydopamine (PDA) via hydrogen bonding and π-π stacking in neutral conditions [238]. Protonation of the amine groups on PDA, DOX, and SN38 weakened their attractive interactions, resulting in the release of the drugs at pH 5. Electrostatic interactions were explored by Yang et al. for the release of diclofenac sodium (DS) from ZJU-101 MOF [463]. The porous structure enabled a large loading capacity, as high as 0.546 mg/mg. In neutral pH, the positively charge MOF adsorbed the diclofenac anion. While in acidic conditions, the anion was neutralized and released from the system. Despite these reports, it is important to note that pH responsiveness does not alleviate the issue with payload leakage for systems in which the cargo is loaded via noncovalent interactions.

In comparison, when the payload is conjugated to the carrier via acid-labile covalent bonds, payload leakage is significantly reduced. An intriguing intramolecular catalysis design by Cao et al. utilized a DOX-pyridinium prodrug that contained a hydrazone bond and an alkyl or oligo(ethylene glycol) (OEG) spacer [451]. The prodrug was then conjugated with pillar[6]arene (WP6) via host–guest reactions to form a supramolecule. At pH 5.5, the carboxylic groups present in WP6 acted as a Brønsted acid and exhibited a high intramolecular catalytic efficiency for degrading the hydrazone bond. Under these conditions, nearly 100% of the payload was released within 30 min. In the absence of WP6, it took over 10 h for 90% of the payload to be released at pH 5.5. In contrast, the release in neutral conditions over 6 h was low, less than 10%. Unfortunately, the system exhibited reduced cytotoxicity compared to the administration of free DOX. They attributed this result to the requirement of endosomal escape for the DDS. However, the novel rapid bond cleavage may be appliable to other DDSs. In another example of intramolecular catalysis, Lv et al. conjugated PTX as the side chain of PEG-b-poly(L-lysine) via β-thiopropionate bonds and prepared micelles using the PTX-containing copolymer [241]. The PTX was protected in the hydrophobic core of the micelle in neutral conditions, and the system exhibited low leakage of the drug. At pH 5, the β-thiopropionate forms a six-membered ring intermediated via intramolecular hydrogen bonding, resulting in rapid hydrolysis of the ester bond and PTX release. As a comparison, the control polymer with a simple ester bond conjugating PTX exhibited a much slower release.

#### 3.1.6. pH-Responsive DDSs: Limitations and Remaining Issues

Though extensively studied, several obstacles lie in the way of the clinical application of pH-sensitive systems. The type and stage of cancer impact the acidification of tumor ECM. This heterogeneity limits the application of DDSs designed to be triggered by the pH of tumor ECM, pH_ex_ [478]. Even within a single tumor, pH_ex_ can vary across the tumor volume. For example, pH_ex_ typically drops from the outer surface toward the center of tumors [408]. However, reaching a region of the tumor with a low enough pH_ex_ to activate release can be limited due to the increased IFP and physical barriers developed in tumors. Other systems rely on the reduced pH within endosomes and lysosomes, pH_en_, to trigger release. In this case, the nanocarriers need to be internalized via the endocytosis pathway, resulting in additional design considerations (Section 2.6.3). Furthermore, appropriate endosomal escape strategies may be required for these systems; otherwise, the payloads released in the late endosomes or lysosomes will be degraded by the lysosomal enzymes and acidic conditions. The readers can refer to several reviews for more detailed information on these issues [474,479,480,481]. Additionally, the pH in inflammation lesions is also reduced; thus, appropriate targeting strategies should be considered to avoid potential systemic toxicity for pH-responsive systems in general [482,483].

### 3.2. Redox-Responsive DDSs

As summarized in Table 3, the initiation of redox reactions has been used to induce release from DDSs using two primary mechanisms: (1) oxidation reactions that enhance the hydrophilicity of one or more components of the DDS and (2) the cleavage of bonds due to either high GSH or ROS levels. Oxidation of thioethers, selenoethers, and telluroethers have been utilized in redox-responsive DDSs based on hydrophilicity changes [49,484,485]. Zhang et al. reported that the increased hydrophilicity achieved by the oxidation of thioethers and disulfides results in enhanced hydrolysis of adjacent ester bonds [277]. Several studies have utilized this behavior to endow DDSs with redox responsiveness [486,487]. Other compounds undergo hydrophilicity changes upon reaction with ROS; for example, the hydrophobic functional group ferrocene can be converted into the hydrophilic ferrocenium ion by reactions with ROS [488]. The process is rapid and reversible with a slight change in Fe-C bond length. Ferrocene moieties can be incorporated in the components of micelles and vesicles or used to form oxidative-cleavable supramolecular structures with β-CD [489,490,491]. The selective oxidation of ferrocene in HeLa cells has been demonstrated in vitro by Noyhouzer et al. [492]. However, studies using ferrocene are still in their infancy compared with other oxidizable groups, and payload leakage is commonly observed in these systems. There is also a lack of reports of in vitro and in vivo testing of ferrocene-containing systems [488].

**Table 3:** Summary of redox-induced response mechanisms used in stimuli-responsive drug delivery systems.

Mechanism	Stimuli-Induced Response	Reference
Oxidation enhanced polarity	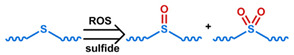	[49] ^b,c,^*** [277] ^a,b,c,^** [486] ^a,b,c,^** [493] ^b,c,^*** [494] ^a,b,c^
	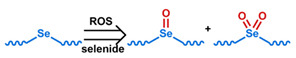	[484] ^c^ [494] ^a,b,c^ [495] ^c^
	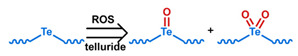	[485] ^a,b,c,^***
	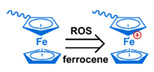	[490] ^c,^*** [491] ^b,c,^*** [492] ^b,c^ [496] ^b,c,^***
Redox-induced bond cleavage	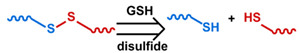 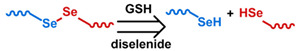 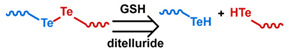	[32] ^a,b,c,^** [44] ^b,c,^*** [45] ^b,c,^*** [46] ^c,^*** [62] ^b,c,^* [117] ^a,b,d^ [163] ^b,d^ [165] ^b,c,^*** [166] ^b,c,^** [180] ^a,b,c,^*** [191] ^a,b,c,^*** [197] ^a,b,c,^** [200] ^b,c,^*** [201] ^b,c,^*** [207] ^b,c,^*** [208] ^b,c,^*** [210] ^c,^*** [227] ^b,c,^*** [228] ^a,b,c,^** [229] ^a,b,c,^* [240] ^b,c,^** [241] ^a,b,c,^*** [332] ^a,b,c,^* [326] ^b,c,^*** [327] ^b,c,^*** [328] ^a,b,c,^*** [494] ^a,b,c^ [496] ^b,c,^*** [497] ^b,d^ [498] ^b,c,^*** [499] ^c,^*** [500] ^b,c,^** (disulfide)
		[494] ^a,b,c^ [501] ^c^ [502] ^b,c,^** (diselenide)
		[503] ^a,b,c,^** (ditelluride)
	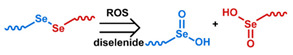	[167] ^b,c,^** [494] ^a,b,c^ [501] ^c^ [502] ^b,c,^** (diselenide)
	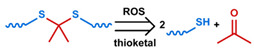	[164] ^a,b,d^ (thioketal)
	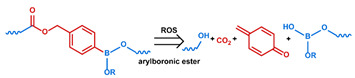	[43] ^a,b,c,^*
	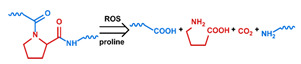	[504] ^b,d^
	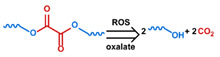	[505] ^a,b,c,^***
Other responses	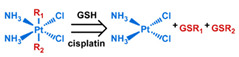	[441] ^a,b,c,^***
	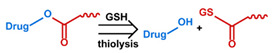	[486] ^a,b,c,^** [487] ^a,b,c,^***
	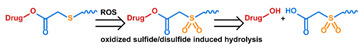	[277] ^a,b,c,^** [486] ^a,b,c,^** [487] ^a,b,c,^***

^a^ Evaluation included in vivo tests on mice.; ^b^ Evaluation included in vitro tests on cells.; ^c^ Small molecules, such as antitumor drugs and dyes, were used as payloads.; ^d^ Large molecules, such as proteins and plasmid DNA, were used as payloads.; * Premature payload leakage was >20%.; ** Premature payload leakage was between 10% and 20%.; *** Premature payload leakage was ≤10%.

In tumors, bond cleavage via redox reactions can be induced by either GSH or ROS. GSH efficiently cleaves disulfides into thiols, and this behavior is the most studied mechanism to impart redox responsiveness to DDSs. Recently, the cleavage of both diselenides and ditellurides has been explored, as they possess lower bond energy (172 and 126 kJ/mol, respectively) than disulfides (240 kJ/mol) [506]. Sun et al. examined the reductive and oxidative sensitivities of four different chalcogen groups and found that the reductive sensitivity of them followed the order of disulfide > diselenide > thioether > selenoether. In contrast, the oxidative sensitivity followed the reverse order [494]. They attributed the difference to the fact that sulfur has a greater electronegativity than selenium and that disulfide and diselenide have greater electronegativity than the thioether and the selenoether. The different groups were used to fabricate prodrugs with PTX conjugated via an ester bond to the chalcogen group. Overall, the best in vivo antitumor efficacy was achieved with a diselenide-based system, suggesting that factors other than reductive or oxidative sensitivity are critical in determining the efficacy of the system. Ma et al. demonstrated the reductive-oxidative dual cleavability of diselenide bonds [501]. The RhB encapsulated in the micelle synthesized with diselenide-inserted polymers was released in 6 h with incubation of either 0.01% H_2_O_2_ or 0.03 mM GSH. The high reactivity does not necessarily impair the stability of the diselenide-containing DDS; for example, in Tian et al.’s work, only ~10% of the payload was leaked in a neutral buffer during 24-h incubation of the diselenide-containing redox-sensitive nanogel [502]. In addition to these chalcogen groups, cleavage of thioketal [164], arylboronic esters [43], and proline bonds [504,507] can also be induced by ROS. The cleavage of bonds via redox reactions has been extended to prodrug systems. For example, Pt(IV) was reduced and released as cisplatin intracellularly in Li et al.’s study [441], and ester-conjugated SN38 was released via GSH thiolysis in Wang et al.’s work [486].

One strategy to enhance the performance of ROS-responsive DDSs is to increase the intracellular ROS level. For example, in Gupta et al.’s work, a higher level of ROS was seen in human coronary artery smooth muscle cells (HCASMCs) when they were incubated with lipopolysaccharide and interferon gamma (IFNγ) [504]. The elevated ROS resulted in higher release rates and gene transfection efficiency for the ROS-sensitive micelle-based DDS studied. In Su et al.’s work, co-administration of D-α-tocopherol poly(ethylene glycol) 1000 succinate (TPGS) was utilized to stimulate more ROS generation from the mitochondrion [43]. ROS-generating compounds can also be delivered to the cells by redox-responsive DDSs. In Li et al.’s work, the prooxidant palmitoyl ascorbate (PA) was co-delivered with camptothecin (CPT). Oxidization of PA by cellular respiration processes generated H_2_O_2_, which cleaved an oxidant-sensitive oxalate bond and released the conjugated CPT [505]. Ye et al. co-delivered β-lapachone with a redox-sensitive DOX prodrug [508]. β-lapachone undergoes a futile redox cycle catalyzed by quinone oxidoreductase type I (NQO1) in cancer cells that efficiently produces ROS [509]. Exogenous stimuli have also been used to enhance ROS levels. Li et al. utilized the photodynamic effect to generate ROS and induced a rapid redox response [485]. The use of exogenous stimuli to enhance ROS concentrations will be further discussed in Section 3.6.

#### 3.2.1. Redox-Responsive Micelles

Oxidization of thio-, seleno-, or telluroether groups can enhance the hydrophilicity of micelle moieties. Increasing the CMC of the amphiphile induces micelle dissociation and drug release. Xiao et al. explored a system in which hydrophobic thioethers were used to connect PEG diacrylate blocks [49]. Stable micelles formed spontaneously at physiological temperatures. Oxidation of the thioether shifted the hydrophilic/hydrophobic balance of the polymer and caused the dissociation of the micelles. However, the response was relatively weak, releasing the payload of Nile red (NR) required an H_2_O_2_ concentration of 50 mM. In contrast, Li et al. reported a fast response via oxidation for their system in which a telluroether was incorporated in the polyurethane (PU) backbone of a PEG-PU-PEG triblock copolymer [485]. The cisplatin payload was released in less than seven minutes. The response was enhanced both by the use of the sensitive telluroether and the co-encapsulation of the photosensitizer, indocyanine green (ICG), to generate ROS upon irradiation. Oxidation of chalcogenide-based bonds has been explored for release from branched polymer systems as well. For example, Liu et al. synthesized a hyperbranched polymer consisting of hydrophobic selenoether-containing segments linked by hydrophilic phosphate nodes [510]. No premature release was observed for the micelles produced from this material. However, in the presence of 0.1 mM H_2_O_2_, the selenoether was oxidized, resulting in the conversion of the hydrophobic segments into hydrophilic segments, and releasing the payload in five hours. Significant cytotoxicity and delivery to cellular nuclei were observed in HeLa cellular assays, a half-maximal inhibitory concentration (IC50) of 0.25 μg/mL was obtained. Interestingly, the DOX-free micelles also exhibited strong cytotoxicity to HeLa cells with an IC50 at 16 μg/mL, while normal NIH3T3 cells were intact. The phenomenon was attributed to the selective selenium-induced cancer cell apoptosis [511]. In Ren et al.’s work, selenoether-bearing pendant groups were placed on hydrophobic blocks of PEO-PAA copolymer [495]. Micelles synthesized from this copolymer dissociated in 0.1% H_2_O_2_ due to the conversion of the selenoether to selenoxide, or selenone, and reassembled when ascorbic acid was added to reduce selenoxide or selenone to selenoether. NR, used as a model drug, was completely released in 24 h when 0.1% of H_2_O_2_ was added to the release medium.

Redox-sensitive bonds can be used as critical linkages in amphiphiles. For example, Pang et al. inserted a ditelluride linkage between folic acid (FA)-terminated hydrophilic PEG blocks and hydrophobic PCL blocks. They used the copolymer to prepare redox-responsive micelles containing DOX [503]. Cleavage of the linkage by GSH led to the destruction of the micelle structure, aggregation of the hydrophobic cores, and significant release of DOX. In a unique self-upregulating approach, Su et al. used ROS-sensitive arylboronic linkages to hang hydrophobic D-α-tocopherol polyethylene glycol 1000 succinate (TPGS) pendants on hydrophilic HA [43]. HA functions as a target ligand for CD44, which is overexpressed on DOX-resistant MCF-7 (MCF-7/ADR) cells, enhancing CD44-mediated endocytosis. After endocytosis of the DDS, TPGS was released by ROS cleavage of the arylboronic linkages. When released intracellularly, TPGS can stimulate mitochondria to generate more ROS and act as a P-gp inhibitor to prevent the efflux of DOX. Thus, the DDS created a positive feedback pathway where initial ROS cleavage of the arylboronic linkages resulted in the production of more ROS via TPGS stimulation. TPGS release also induced micelle dissociation and DOX release. The system exhibited excellent cytotoxicity and accumulation in MCF-7/ADR cells during in vitro testing. The in vivo study of MCF-7/ADR tumor-bearing mice demonstrated tumor growth retardation compared to free DOX and micelles using ROS-insensitive links for TPGS.

The use of redox-sensitive cross-links in micelles has also been explored. Zhu et al. used the disulfide-containing crosslinker, propargyl 3,3′-dithiopropionate, to stabilize a methoxy poly(ethylene glycol)-b-poly((ε-caprolactone)-co-(5,5-diazidomethyl trimethylene carbonate)) (mPEG-b-PDATCL) micelles [45]. The network effectively reduced the leakage of PTX from 80% to 10% over 24 h in phosphate-buffered saline (PBS). However, when 10 mM dithiothreitol was added, the cross-links were broken, and ~60% of the payload was released over 24 h. The cytotoxicity of the PTX-loaded DDS was greater than the redox-insensitive control DDS but slightly lower than for free PTX. This effect is most likely due to the delayed PTX arrival to the cytosol via the endocytosis pathway. Both free PTX and the PTX-loaded redox-sensitive DDS exhibited higher efficacy than the redox-insensitive control DDS.

#### 3.2.2. Redox-Responsive Liposomes

Most reports of redox-sensitive liposome-based DDSs utilized lipids with redox-cleavable linkages. Breakage of these links destabilizes the liposome. Xu et al. used a combined pH- and redox-responsive liposome to deliver DOX [332]. They synthesized lipids containing a disulfide bond between the hydrophilic hexahydrobenzoic amide-modified histidine head group and the hydrophobic tails. The unique head group endowed the liposome with pH responsiveness to tumor ECM by enabling a reversal of the liposome surface charge from negative to positive at a pH of ~6.5. The potential was further increased as the hexahydrobenzoic amide was hydrolyzed, exposing the histidine group in late endosomes or lysosomes. Histidine promotes endo/lysosome escape of the nanocarriers by increasing the influx of anions by buffering the endo/lysosome, i.e., the proton sponge effect. This influx lyses the organelle due to increased osmotic pressure [481]. Finally, the disulfide bond was cleaved by GSH in the cytosol destabilizing the liposome and releasing the payload. The efficacy of the strategy was confirmed by the significant DOX accumulation in HepG2 cells observed under CLSM. In a pH 5.5 buffer, the acidic conditions accelerated drug release indicating that pH was a co-stimulus. However, more release occurred after the addition of GSH. After 24 h incubation in a GSH solution, the liposomes were completely degraded. Higher cytotoxicity was observed for free DOX than the pH-redox dual responsive DDS. However, the in vivo test to hepatocellular carcinoma (Heps) in tumor-bearing mice demonstrated that the level of DOX that accumulated in the tumor cells was much higher when the liposome-based DDS was tested than when free DOX was used. The tumor inhibitory effect was also higher with the liposome-based DDS. Candiani et al. used an intriguing disulfide-bridged Gemini surfactant, SS14, in the fabrication of a redox-responsive liposome [497]. Cleavage of the disulfide group converted the surfactant into two separate amphiphiles and promoted disassembly of the liposome. Gene transfection efficiency was elevated as the fraction of SS14 in the liposome was increased to 50 mol%. A GSH depletion and repletion cell assay revealed that the transfection efficiency varied linearly with GSH content. Sun et al. prepared a dual redox- and enzyme-sensitive liposome based on a lipid that used disulfide bonds to connect a hydrophobic tail to a histidine-containing head [117]. Alkyne-functionalized HA conjugated to the surface of the liposomes via click reaction with azide-containing cholesterol also included in the liposome structure, formed a protective shell and targeted CD44. The stability of the system was confirmed by the lack of change in particle size and negligible HA detachment in pH 7.4 and pH 6.5 buffers, and serum medium buffered to pH 6.5. The encapsulated silencing RNA (siRNA) was only released in the presence of both hyaluronidase and high levels of GSH. It also exhibited a longer circulation time in mice as compared to a control system where the HA was physically adsorbed to the surface. Degradation of the HA shell by hyaluronidase in endosomes exposed the histidine groups, promoting endosomal escape. GSH in the cytosol then broke the disulfide bond, releasing the payload.

Redox responsiveness can also be endowed to liposomes via establishing a redox-sensitive polymeric shell on the liposome surface. In Zhao et al.’s work, a 3,4,5-triproparyloxybenzoic group was grafted onto a palmitoyl oleoyl phosphoethanolamine (POPE) head group to prepare a cross-linkable lipid [512]. After the preparation of the liposome, further crosslinking of the surface was done through the creation of disulfide bridges via azide-alkyne cycloaddition click reactions. Crosslinking the liposome surface was effective at reducing leakage of the payload, carboxyfluorescein (CF). Leakage at pH 7 of encapsulated dyes was suppressed from 60% to less than 20% in 5 h after crosslinking. When dithiothreitol (DTT) was added, the disulfide bonds were broken, and a burst release of the cargo was observed.

#### 3.2.3. Redox-Responsive Polymeric Nanoparticles

Redox-responsive polymeric nanoparticles are mostly fabricated with redox-cleavable linkages inserted in the backbone of the polymer chains. Kozielski et al. synthesized polymers from a series of monomers, including a disulfide-containing monomer [163]. Polymeric nanoparticles carrying apoptosis siRNA were then prepared. They found that by altering the polymer formulation, nanoparticles with different redox sensitivity and hydrophobicity were obtained. The optimized nanoparticle possessed good stability, and both high siRNA delivery efficiency and selective toxicity, higher delivery to primary human glioblastoma (GBM 319) cells versus normal human fetal neural progenitor (fNPC 34) cells. Thioketal groups also react in a highly selective fashion with ROS and remain stable against acid-, base- or protease-catalyzed degradation [513,514]. Wilson and his coworkers, therefore, developed polymers with thioketals in the backbones to prepare ROS-sensitive DDSs for the delivery of therapeutics to intestinal tissue via oral administration [164]. The siRNA payload was mixed with cationic lipid and encapsulated in the ROS-sensitive polymers via an emulsion method. The cationic lipid formed a complex with the siRNA stabilizing the structure and promoting endosomal escape. The in vivo therapeutic efficiency was confirmed by the normalized body weight and lower inflammatory responses in colitis-bearing mice treated with the siRNA-loaded ROS-responsive nanoparticle.

In contrast to placing the cleavable group in the polymer backbone, in nanogel-based DDSs, redox sensitivity can be achieved by using redox-cleavable crosslinkers. For example, in Ohya et al.’s work, a dextran nanogel was stabilized by hydrophobic-hydrophobic interaction of oligolactide groups connected to the dextran backbone via disulfide bonds [168]. Galactose residues present in the system enhanced galactose receptor-mediated endocytosis by HepG2 cells, and oligo(ethyleneimine), also grafted to the backbone, promoted endosome escape via its buffering capacity. Degradation of the nanogel was observed after 4-h incubation in 10 mM GSH, and pyrene, the model hydrophobic drug, was released in 10 mM DTT, demonstrating the redox sensitivity of the system. The targeting efficiency of the galactose residue, the endosomal escape due to the oligo(ethyleneimine), and GSH-cleavage of the disulfide group were all confirmed in HepG2 cell assays.

#### 3.2.4. Redox-Responsive Porous Inorganic Nanoparticles

One route to redox-responsive behavior in inorganic nanoparticle-based DDSs is to anchor gatekeepers via redox-cleavable linkages. For example, in Zhang et al.’s work, β-CD was attached via disulfide bonds to block MSN pores [326]. In addition, PEG and folic acid (FA) were attached to adamantane and immobilized onto the MSN surface via the host–guest interaction between adamantane and β-CD. Due to the presence of the target ligand, FA, and the GSH-labile disulfide linkage, the MSN showed a comparable IC50 to free DOX on HeLa cells (0.65 μg/mL), and a high IC50 value for normal human embryonic kidney (HEK) cell (13.56 μg/mL). Furthermore, in the absence of the FA ligand, the MSN still exhibited a relatively low IC50, 1.26 μg/mL, for HeLa cells and high IC50, 11.84 μg/mL, for HEK cells. This result demonstrates the high level of GSH in cancer cells. Incidentally, the β-CD was modified with additional amine groups, which imbued the system with pH responsiveness due to the Coulombic repulsion between protonated gatekeepers in acidic conditions, causing the pores to open. The proton buffering capacity of the amine groups also enhanced endosomal escape. In contrast, Giménez et al. used a thiol-containing trimethoxysilane to modify the surface of an MSN [498]. They then linked PEG to the MSN via disulfide bonds with the thiol group fabricating a gatekeeper. Less than 10% of the drug leaked in medium with a GSH level of blood (~2 μM). However, rapid release was achieved in a medium containing an intracellular GSH level (10 mM), over 90% of the DOX was released within one hour. They attributed the well-regulated release to the short distance between active disulfide groups and the MSN surface. Zhao et al. modified both the surface and pores of an MSN with the thiol-containing trimethoxysilane [499]. Due to the small pore size, 2.5 nm, of the unmodified MSN, the small molecule drug, 6-mercaptopurine (6-MP), bound to the thiol group by a disulfide bond, was capable of blocking the pores and serving as a gatekeeper. The system showed no drug leakage at pH 7.4, while a burst release of 6-MP was observed after the addition of 3 mM GSH to the release medium. However, the loading capacity of the system was low, <5% by mass. They attributed this to the lack of affinity between the polar silica and hydrophobic 6-MP, as well as to the fact that the 6-MP formed the pore blocking conjugates during loading, blocking further 6-MP loading.

Redox-initiated bond cleavage in protective polymeric membranes on inorganic nanoparticles has also been used to endow MSN- and MOF-based DDSs with redox sensitivity. Typically, these membranes consist of polymers crosslinked via disulfide bonds. The overexpression of GSH in tumors acts to quickly break these bonds, releasing the cargo. This behavior is often combined with other techniques to enhance tumor cell uptake. In Palanikumar et al.’s work, a comb-like polymer with pyridine disulfide hydrochloride (PDS) and PEG on side chains was used to fabricate a polymeric membrane gatekeeper [327]. DTT was added to induce disulfide exchange reactions and form a crosslinked membrane on an MSN surface. Residual disulfide groups were used to conjugate thiol-terminated arginyl glycyl aspartic acid (RGD) ligand to confer targeting capability to the DDS [515]. The redox sensitivity of the system could be tuned by adjusting the crosslink density. With 19% crosslinking density, the premature release was suppressed to less than 10% without GSH, while the addition of 5 mM GSH to the release medium resulted in a burst release of payloads due to degradation of the polymeric membrane. In addition to crosslinked polymer coatings, protective coatings can also be formed by dense attachment of large molecules to the particle surface. In Wang et al.’s work, a GSH-sensitive protective membrane was attached to the surface of a MOF via disulfide bonds [180]. β-CD was functionalized with a bicyclononyne group via disulfide bonds. The β-CD derivative was then conjugated onto azide-modified MOF via an azide-alkyne click reaction. Due to the abundant azide groups on the MOF, the high efficiency of the click reaction, and the large size of the β-CD group, a dense layer was coated on the surface. This dense layer prevented the degradation of the MOF [516]. In addition, adamantane-terminated RGD ligands were fixed via host–guest interaction with the exposed β-CD, and a PEG block was then covalently bound to the exposed RGD ligand via a pH-liable benzoic imine bond. The functionalization protected the DDS during circulation, allowed the weakly acidic conditions of the ECM to expose the RGD enhancing endocytosis, and enabled the exposed RGD in weakly acidic tumor ECM to enhance endocytosis. The β-CD coating was then removed in the cytosol due to the high level of GSH releasing the payload. The DDS exhibited a comparable tumor suppression effect to free DOX. However, the combination of effective protection afforded by the membrane, the pH-sensitive targeting functionality, and the redox-sensitive release mechanism resulted in a significant reduction in weight loss in mice as compared to those receiving the free DOX treatment.

In addition to attaching gatekeepers or forming protective coatings, redox-cleavable linkages can be used as precursors for MSN systems. In these cases, redox reactions disrupt the particle structure leading to breakup and payload release. For example, Zhang et al. used a disulfide-bridged silane to produce a hollow silica nanoparticle loaded with DOX [191]. Antioncogene, p53, was complexed to β-CD terminated polycations attached to adamantane-containing silane, which were also decorated on the MSN after synthesis. In a reductive environment, the system degraded in several days, releasing both the drug and the gene. The effectiveness of the system was demonstrated by the inhibition of glioma growth on mice during in vivo testing. Disulfide bonds can be incorporated in multiple locations to enhance responsiveness. For example, in Wu et al.’s work, both a disulfide-bridged precursor and a disulfide-linked gatekeeper, PTX prodrug, were used in an MSN-based DDS [197]. The photothermal agent indocyanine green (ICG) and ultrasound imaging agent, perfluoropentane (PFP), were encapsulated as cargo in the system. The payload release was faster than Zhang et al.’s system under similar reductive conditions [191], probably due to the mesoporous structure. The evaporation of PFP induced by temperature elevation due to the photothermal effect of ICG not only enhanced US imaging contrast, but also promoted cellular uptake of nanocarriers by bubble-induced cytomembrane disruption. During the in vivo tests of the system on tumor-bearing mice, a reduction in tumor size was observed, accompanied by negligible weight loss. Redox-cleavable precursors have also been used to develop MOF-based DDSs. In Lei et al.’s work, a disulfide-bridged organic ligand was used to fabricated MOF for curcumin delivery [229]. One issue with these redox-degradable MOF and MSN DDSs is that the release profiles are not as well defined as those that use a gatekeeper strategy. In addition, the redox-initiated degradation, and therefore drug release, is slow, typically taking several days, and premature release is typical for MOF-based DDSs that do not utilize a gatekeeper [192,228,229].

Finally, redox induced hydrophilicity changes have also been utilized to endow inorganic nanoparticle-based DDSs with redox-sensitivity. Cheng et al. used the strongly hydrophobic phenyl sulfide (PhS) to block pores on an MSN-based DDS [493]. The “air-gate” that resulted from the nonwetting PhS completely inhibited drug leakage. Oxidation of the sulfide into sulfone or sulfoxide upon reaction with ROS enabled the diffusion of the hydrophilic payload, DOX, from the system. Cytotoxicity assays indicated high toxicity for human breast adenocarcinoma (MCF-7) cells and the low toxicity for normal human endothelial (HUVEC) cells, confirming that the ROS-sensitive DDS was selective in delivering the payload to cancer cells.

#### 3.2.5. Redox-Responsive Non-Encapsulated DDDs

The cleavage of many redox-sensitive groups makes them especially suitable for application to prodrug and particle drug conjugates. Often this response is used in conjunction with other stimuli-responsive behavior to release the payload. For example, Kang et al. reported a dual-redox-responsive micellar drug-conjugate [496]. An amphiphilic moiety was fabricated using a β-CD-terminated PEG chain and a disulfide-bonded ferrocene-CPT. Both parts were connected via host–guest interactions between β-CD and CPT. Both the oxidation of ferrocene by ROS and the cleavage of the disulfide bond by GSH-induced micelle dissociation and drug release. The system exhibited appropriate redox sensitivity. At normal cell GSH levels, ~2 mM, ~40% of the loaded CPT was released in 24 h, while in 10 mM GSH, the CPT was completely released in ten hours. At normal cellular H_2_O_2_ levels, ~1 μM [517], less than 20% of the CPT was released in 120 h, while in pathological conditions, 50 μM H_2_O_2_, over 25% of the CPT was released in 24 h. The redox sensitivity was corroborated in cell assays. The drug-loaded DDS displayed cytotoxicity as high as free CPT in lung cancer cells (A549), while reduced toxicity to normal lung fibroblast (HLF) cells. Premature release is typically observed in redox-sensitive MOF systems with weak interactions between the payload and the particle [228,229]. Gong et al. addressed this problem by covalently bonding the payload to the MOF structure [227]. They used a thiol-bearing organic ligand in the fabrication of an MOF DDS to enable the formation of disulfide bonds between the MOF and the model drug, 6-mercaptopurine (6-MP). The system exhibited a relatively high loading capacity, 35 mg/g, and there was little premature release in the absence of GSH. However, a rapid release of 6-MP was observed in 5 mM GSH; over 50% of the payload was released in 4 h.

Three additional techniques have been explored in non-encapsulated DDSs: reduction of cisplatin prodrugs, thiolysis of esters, and sulfide oxidation-enhanced ester hydrolysis. The cisplatin prodrug c,c,t-[Pt(NH_3_)_2_Cl_2_(OH)(O_2_CCH_2_CH_2_CO_2_H)] was conjugated with poly(amidoamine) via amidation in Li et al.’s study [441]. The dendrimer prodrug was then attached to PCL blocks via methyl maleic amide, and micelles were fabricated from the PCL-dendrimer amphiphile. The system was stable under neutral conditions, with only 10% leakage in 8 h. When 5 mM ascorbic acid (AA) was added, cisplatin was released completely in 4 h. Wang et al. conjugated SN38 to OEG via a short dithioether chain and an ester bond to form a prodrug system that responded to both ROS and GSH [486]. The oxidative response was attributed to the enhanced hydrolysis of the ester bond due to the enhanced hydrophilicity nearby when the dithioether was oxidized, and the reductive response was due to the thiolysis of the ester bond via GSH. The enhanced hydrolysis of ester bonds obtained when neighboring thioether groups are oxidized was also studied by Zhang et al. [277]. In their work, maleimide was conjugated to PTX via ester bonds to either a thioether- or disulfide-containing linker to form a prodrug. The amphiphilic feature of the prodrug enabled it to assemble into a micelle in an aqueous medium. The thiol groups on albumin were used to bond it to the maleimide forming a protective protein shell. This albumin shell enhanced the biocompatibility and in vivo circulation time of the DDS. In release tests, both the thioether and the disulfide facilitated the hydrolysis of the ester bonds and PTX release when they were oxidized by H_2_O_2_. At an H_2_O_2_ level of 2 mM, the system that used the disulfide linker exhibited less hydrolysis-catalyzing capability than the system that used a thioether linker. This can be attributed to the higher electronegativity and lower oxidation state of the disulfide bond. At a higher H_2_O_2_ level (10 mM), the system that utilized the disulfide bond induced hydrolysis of the ester bond and PTX release that was comparable to or greater than that observed for the thioether linker, probably due to the additional sulfur atom. The in vitro assay demonstrated selective cytotoxicity of the prodrug-based micelle to murine mammary carcinoma (4T1) cells, and lower toxicity to NIH3T3 cells. The biodistribution test to 4T1 tumor-bearing mice demonstrated a longer and more stable circulation of the micelle with albumin formed protein shell than the control micelle without the shell. The micelles with the protein shell also achieved the highest tumor accumulation, which was also higher than in other tissues after 24 h, in comparison to micelles without the protein shell. A suspension of tumor growth and negligible weight loss were observed with the micelle treatment.

#### 3.2.6. Redox-Responsive DDSs: Limitations and Remaining Issues

One issue with the use of redox stimuli in DDSs is the potential for “off-target” delivery. Along with tumor tissues, lesions, inflammations, infections, and cells associated with other chronic diseases often have elevated ROS levels [423,424,425,426]. In addition to tumor cells, GSH levels are also elevated in hepatocytes; levels up to 10 mM have been observed [518], high enough to induce a response in many redox-sensitive DDSs. Another issue is that while the environment in the endosome and lysosome usually favors oxidation, the acidic conditions are unfavorable for GSH-induced breakage of disulfide bridges. Thus, reductive-sensitive DDSs that enter the cells via endocytosis must escape the endosome or lysosome and be exposed to the high GSH levels in the cytosol [519,520]. Therefore, rational drug delivery design and multi-responsiveness should be considered in the development of redox-responsive systems.

### 3.3. Enzyme-Responsive DDSs

Many enzymes are typically overexpressed in tumors, including proteases, phospholipases, hyaluronidases, and oxidoreductases. A summary of the enzymes that have been exploited for enzyme-responsive DDSs and the bonds they are capable of breaking, as well as their substrates, are summarized in Table 4. The primary location of the target enzyme within the tumor tissue affects their interaction with DDSs. Hydrolases such as phospholipase A2 (PLA_2_) [433,434,435], phospholipase C (PLC) [124,436], cathepsin B [521,522,523,524], cathepsin D [525], and hyaluronidases [432] are overexpressed in many cancer cells and can cleave their respective substrates intracellularly. PLA_2_ and PLC are present on cellular membranes and can interact with DDSs that come into contact with the cell walls. Cathepsin B, cathepsin D, and hyaluronidase-1 are present in lysosomes and can attack DDSs internalized via the endocytosis pathway. Hyaluronidases are also expressed in the tumor ECM [526]. Matrix metalloproteinases (MMPs) can degrade peptide substrates in the tumor ECM [437], and quinone oxidoreductase type I (NQO1), which cleaves quinone propionic acid groups and azobenzene groups in the presence of nicotinamide adenine dinucleotide phosphate (NADPH), is present in the cytosol [438,527,528,529]. In addition to enzymes that are overexpressed in tumors, those that are expressed by specific tissue or organs can also be employed to stimulate drug release from DDSs that arrive at those tissues. For example, β-D-galactosidasease is expressed in primary ovarian cancers and at the brush border membrane of the small intestine [205,530,531], α-amylase and lipase are upregulated in acute pancreatitis [182], trypsin protease is excreted by the pancreas and activated in the small intestine [190], and azoreductase is expressed by the microflora of the colon [47,328,532,533].

**Table 4:** Summary of enzyme-induced response mechanisms used in stimuli-responsive drug delivery systems.

Mechanism	Enzyme and Substrate	Reference
Cleavage by hydrolases	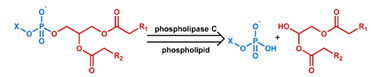	[124] ^c^
	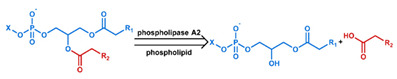	[120] ^c,^***
	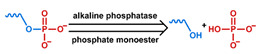	[534] ^c,^**
	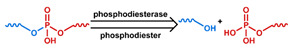	[535] ^b,c,^**
	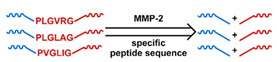	[160] ^b,c,^*** [329] ^b,c,^** [536] ^b,d^ [537] ^a,b,c,^**
	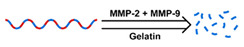	[157] ^a,b,c,^***
	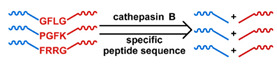	[261] ^a,b,c^ [242] ^c^ [243] ^a,b,c^ [265] ^a,b,c,^*** [330] ^b,c,^*** [538] ^b,c,^*** [539] ^a,c,^*** [540] ^a,b,c,^*** [541] ^a,b,c^
	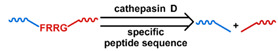	[525] ^a,b,c,^*
	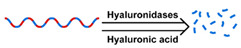	[19] ^a,b,c,^* [212] ^b,c,^*** [542] ^a,b^ [543] ^a,b,c,^* [544] ^b,c,^**
	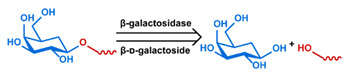	[205] ^b,c,^*** [530] ^a,b,c,^*** [531] ^a,b,c,^***
Cleavage by reductases	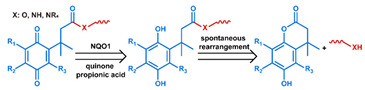	[545] ^c^
	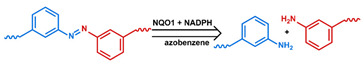	[529] ^a,b,c,^***
Cleavage by other enzymes	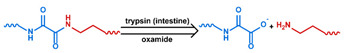	[190] ^b^
	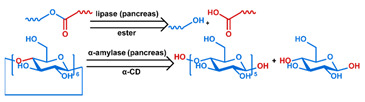	[182] ^c,^***
	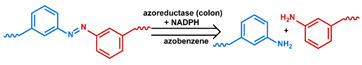	[47] [328] ^a,b,c,^*** [532] ^b,c,^** [533]

^a^ Evaluation included in vivo tests on mice.; ^b^ Evaluation included in vitro tests on cells.; ^c^ Small molecules, such as antitumor drugs and dyes, were used as payloads.; ^d^ Large molecules, such as proteins and plasmid DNA, were used as payloads.; * Premature payload leakage was >20%.; ** Premature payload leakage was between 10% and 20%.; *** Premature payload leakage was ≤10%.

Interestingly, enzymes can also serve as a therapeutic agent. One of the most studied examples of this idea is starvation and chemodynamic therapy via glucose oxidase (GOx) [546]. GOx was initially explored as an endogenous stimulus to induce pH reduction due to its promotion of gluconic acid production in diabetes patients [547]. However, delivery of this enzyme to cancer cells can exhaust glucose and convert oxygen to ROS intracellularly. Combined, these two effects promote cancer-cell death. Furthermore, the H_2_O_2_ released by GOx can be converted to O_2_ by co-delivered catalases and promote nanocarrier diffusion, as shown in You et al.’s work [548]. Such strategies are beyond the scope of this review; for further information, the readers are directed to a review by Fu et al. [549].

#### 3.3.1. Enzyme-Responsive Micelles

In micellar systems, enzyme cleavable bonds have been used within the hydrophilic and hydrophobic polymer blocks, as a link between these blocks, to attach pendant groups, and within crosslinks used between amphiphiles. Breaking of these bonds results in micellar breakup due to the destabilization of the system, (Figure 1a–d). In addition to stimulating the release of payload, enzymatic cleavage has also been used to promote endocytosis and endosomal escape in DDSs.

The most common technique is to incorporate the cleavable bond in the polymer backbone. Wooley’s team used poly(lactic acid) (PLA), which is degradable by proteases, as hydrophobic blocks for the synthesis of a protease-responsive micelle [550]. Degradation of the hydrophobic core occurred as the enzyme diffused through the hydrophilic poly(N-acryloyloxy)succinimide-copolymer-N-acryloylmorpholine shell. The NR payload was released as the micelle destabilized. In later studies, they investigated the influence of charge on proteases and the hydrophilic surface during hydrolysis [551]. As expected, when the proteases and the hydrophilic shell had opposite charges, a significantly higher PLA degradation rate was observed. This result suggests that the micellar surface charge should be considered in the development of enzyme-responsive DDSs. In another study, Hu et al. used a phosphoester-based polymer to fabricate enzyme responsive micelles [535]. While the phosphoester on the backbone can be hydrolyzed via both acid conditions and by phosphodiesterase I, significantly higher hydrolysis rates and drug release was observed in the presence of the enzyme. Choi et al. developed a HA-based amphiphile by attaching hydrophobic 5 β-cholanic acid and PEG pendant groups to HA [542,543]. Due to the selective binding of HA to CD44, the micelle was efficiently captured by cancer cells and degraded by overexpressed hyaluronidase-1 (Hyal-1) intracellularly but was not degraded in normal cells.

In contrast to degradable blocks, a single enzyme-cleavable bond can be used to connect the hydrophilic and hydrophobic blocks of the amphiphile. Zhang et al. linked PEG and PCL blocks using an azobenzene bond [328]. They also prepared an amphiphilic CPT prodrug using disulfide-linked PEG chains. The micelle-based DDS included both amphiphiles. Efficient CPT release was only achieved in high levels of both GSH and azoreductase. In the absence of high levels of GSH, to cleave the disulfide links, the system exhibited leakage of less than 10% even when azoreductase, to cleave the azo bonds, was added. In the presence of 10 mM GSH and no azoreductase, only ~30% of the payload was released, as the intact azo-bond preserved the micelle integrity. When both GSH and azoreductase were present, ~80% of the CPT was released over 48 h. Both stimuli are present in hepatocytes, GSH in the cytosol and azoreductase in the microsome, suggesting the use of such a combined stimuli-responsive DDS for the treatment of hepatic cancers.

Enzymatic cleavage of pendant groups to change the micelle CMC has also been studied in polymer micelle-based DDSs. Harnoy et al. conducted a proof of concept study on the use of penicillin G amidase (PGA) cleavable amides to attach hydrophobic phenyl acetamide groups to PEG as pendant groups [552]. NR was encapsulated in micelles composed of the PEG/phenyl acetamide amphiphile. They found that PGA was capable of diffusing through the hydrophilic shell and cleaving the amide. The released phenylacetic acid destabilized the micelles and released the NR payload. The amide bond was found to be inert to esterase, suggesting the potential selectivity of the enzyme response.

Enzymatic cleavage of stabilizing crosslinking bonds to enable payload release from micelle-based DDSs was explored by Wang et al. [534]. The prepared micelles were crosslinked with adenosine 5’-triphosphate (ATP). ATP is negatively charged with a pKa of 0.9 and formed strong hydrogen bonds with amine groups in the micellar core that prevented micellar breakup even at significant dilution. However, in the presence of phosphatase, ATP degradation effectively cleaved the crosslinks, leading to micelle dissociation and payload release.

#### 3.3.2. Enzyme-Responsive Liposome

Enzymatic degradation of lipids leads to liposome membrane destabilization and fusion. Phospholipids are intrinsically sensitive to phospholipases, which can therefore serve as a stimulus for phospholipid-based liposomes. No leakage of loaded doxycycline was observed from liposomes in the absence of PLA_2_ in a study by Thamphiwatana et al. [120]. However, ~70% release was achieved after PLA_2_ was added. While their evaluation focused on the delivery of antibiotics, the overexpression of PLA_2_ in inflammation and cancer cells suggests a similar system could be used for the delivery of therapeutics to cancer cells [434,553]. Phospholipase activity is also enhanced in acidic conditions. Shimanouchi et al. [124] studied the rate of PLC-induced liposome membrane fusion as a function of pH. They found that PLC could facilitate destabilization of the liposome and promote fusion with the endosome membrane by two mechanisms: (1) degradation of the lipids via its enzymatic activity and (2) perturbing the membrane through hydrophobic interactions. The highest rates of membrane fusion were observed at pH 5, which corresponds to the pH of late endosomes and lysosomes. This study significantly enhanced the level of understanding of the lipid membrane fusion process during drug delivery.

In addition to phospholipids, other substrates can be placed on lipids to impart enzymatic sensitivity. Recovery of fusogenic lipid behavior is a common theme. In Pak et al.’s study, a peptide substrate of elastase was conjugated at the hydrophilic head of the fusogenic lipid, DOPE [554]. Cleavage of the substrate changed the charge state and intrinsic curvature of the lipid. As a result, the fusogenic activity of DOPE was recovered, and fusion with other lipid bilayers was promoted. In another example, McCarley et al. attached quinone propionic acid to the head of DOPE via an amide bond [545]. The quinone was reduced by NQO1 to benzene-1,4-diol, which then induced amide bond cleavage via interactions with the carboxyl group. Destabilization of the liposome after amide cleavage resulted in the system converting to a micellar structure at pH 7. Such a conversion would lead to the release of payload. In a further study, they explored the dependence of NQO1 catalytic activity on the structure of the quinone propionic acid derivative. They found that the substituent located in the meta-position relative to the propionic acid on the quinone possesses a significant impact [527]. Compared with pH- and redox-mediated controlled release, the studies of enzyme-mediated liposome DDSs are not as extensive as studies of pH- and redox-mediated liposome DDSs. However, given the broad presentation of phospholipase in biological systems and the overexpression of PLA_2_ in several disease processes, further exploration is warranted [434,436,553].

#### 3.3.3. Enzyme-Responsive Polymeric Nanoparticles

Some natural polymers are intrinsically degradable by enzymes that are overexpressed in tumors. Thus, a polymeric nanoparticle fabricated by these materials possesses an inherent enzymatic response. Enzyme-sensitive residues can also be incorporated into the backbone of synthetic polymers to imbue them with enzyme sensitivity.

As observed in Choi et al.’s work, HA is efficiently internalized by cells that have HA receptors [543]. For example, both A549 cells with overexpressed CD44 and hepatoma-22 (H22) cells with highly expressed CD168 receptors exhibit enhanced endocytosis of HA-based nanogels. HA can also be readily degraded by hyaluronidases, especially hyaluronidase-1, which is overexpressed in many malignant tumor cells [555]. Thus, the use of HA offers both enhanced cellular uptake by several cancer lines and also enables hyaluronidase-responsive release. Yang et al. fabricated an enzyme-responsive DDS based on these phenomena [19]. The hydrogel was composed of HA modified with methacrylic anhydride to introduce vinyl groups. The modified HA was then crosslinked by reaction with di(ethylene glycol) diacrylate. Degradation via hyaluronidase released the encapsulated DOX. However, the significant liver accumulation of HA nanoparticles observed in both Choi et al.’s work on HA-based micelles and Yang et al.’s study of HA nanogels may lead to undesired side effects for these systems [19,543].

Gelatin is digested by MMP-2 and MMP-9, both of which are found in the liver and overexpressed in tumors [556,557]. In Long et al.’s work, DOX was intercalated in oligonucleotide (ODN) and encapsulated by gelatin [157]. The degradation of both gelatin and ODN in tumor ECM by MMPs and DNase I induced DOX release. To prevent degradation of gelatin and ODN in the liver and blood, respectively, a PEGylated and histamine-modified alginate shell was added [557,558]. This shell was removed by protonation at pH below 6.9, a pH found in tumor ECM but not the liver. The multilayer structure exhibited no leakage in saline, while in tumor homogenate (THS), DOX was completely released in 24 h. When injected to tumor-bearing mice, the nanoparticles were predominately accumulated in tumors rather than other organs.

Synthetic polymers can also be formulated that are enzymatically degraded. Dorresteijn et al. synthesized a nanoparticle composed of a triblock copolymer, polylactide-b-peptide-b-polylactide, through nonaqueous emulsion polymerization [160]. MMP-2 can digest the peptide sequence used, reducing the MW of the polymer and resulting in a reduction in the glass transition temperature (T_g_) of the system to below physiological temperatures, enhancing permeability, and releasing the payload. While the release rate was relatively slow, three days were required to achieve ~35% release, cytotoxicity, even higher than that of free drug, was observed in cell assays. As a comparison, the copolymer produced with a peptide not sensitive to MMP-2 digestion did not exhibit responsive release.

#### 3.3.4. Enzyme-Responsive Porous Inorganic Nanoparticles

A widely used method to confer enzyme responsiveness to MSN nanoparticles is the use of peptides to immobilize gatekeepers. In Cheng et al.’s work, an α-cyclodextrin (α-CD) gatekeeper was noncovalently absorbed through interactions with aminopropyl groups present on the surface of an MSN [330]. An RGD targeting ligand was then conjugated to the aminopropyl groups through a cathepsin B-cleavable peptide linker. The RGD ligand sterically hindered the removal of the α-CD preventing leakage of the DOX payload even at low pHs. However, after endocytosis, cathepsin B cleaved the peptide, removing the RGD ligand. In the low pH environment of later endosomes and lysosomes, the affinity between α-CD and the aminopropyl groups was weakened, allowing α-CD detachment from the DDS and release of the DOX. A highly selective release of DOX and high cytotoxicity of the system was observed in HeLa cell assays due to the RGD-mediated endocytosis and cathepsin B-induced gatekeeper removal. In another work, Liu et al. used conjugated human serum albumin (HSA) as gatekeepers on MSN [537]. The peptide linker used was composed of a cell-penetrating peptide (CPP) and an MMP-2 substrate. Effective prevention of DOX leakage in the absence of MMP-2 and rapid release of DOX in its presence was observed. A phenylboronic acid (PBA) ligand was also decorated on the HSA to achieve targeted delivery to hepatoma carcinoma cells. The CPP and PBA resulted in nearly twice the uptake by HepG2 cells than for normal cells. Li et al. fabricated a novel gatekeeper that consisted of a cationic CPP conjugated to an anionic peptide via cathepsin B cleavable peptide linker [538]. The neutral charge of the linked species enhanced circulation time, and when the peptide linker was cleaved in tumor ECM, endocytosis was efficiently mediated by the exposed CPP.

The second approach to endow MSN- and MOF-based DDSs with enzyme sensitivity is to coat the particles with enzyme-degradable shells. The coating limits leakage of the payload during transport to the tumor site, after which removal of the coating enables the release of the therapeutic agent. In Chen et al.’s work, HA was conjugated to the surface of an amino-functionalized MSN-based DDS. The coating prevented the release of the drug during transport to the tumor and provided CD44 targeting behavior [212]. Kim et al. applied this idea to MOF-based DDSs; HA layers were fabricated onto Zr-based porphyrinic MOF, PCN-224, via physical adsorption after DOX loading [544]. The porous structure and strong noncovalent interaction led to a high loading capacity of 1.08 mg DOX/mg MOF. The HA shell effectively eliminated drug leakage, and selective cancer cell cytotoxicity was observed. In addition, the organic ligand used in the MOF, (4-carboxyphenyl)porphyrin, is also a photosensitizer. Thus, the system exhibited enhanced cytotoxicity when irradiated with a 640 nm light source.

Although not as extensively studied, enzyme-cleavable linkages have also been incorporated into MOF or MSN systems to endow them with enzymatic response behavior. Fatieiev et al. conducted a proof-of-concept study in which an oxamide-bridged alkoxysilane was used in silica nanoparticle synthesis [190]. They followed nanoparticle degradation by transmission electron microscopy (TEM) and dynamic light scattering (DLS); complete degradation of the nanoparticle occurred in 48 h when trypsin protease was included in the medium.

#### 3.3.5. Enzyme-Responsive Non-Encapsulated DDDs

Several drug conjugates have also been synthesized with enzyme-cleavable substrates. In one of the earliest manifestations of this technique, Duncan and his coworkers conjugated antitumor drugs to N-(2-hydroxypropyl)methacrylamide (HPMA) using cathepsin B-degradable peptide linkages [242,539]. The prodrug structure released the payload in several hours. It exhibited lower systemic toxicity, longer circulation time, and enhanced accumulation of the drug when evaluated in a mice model [265]. More recently, Gu’s group applied cathepsin B-degradable peptide linkers to dendritic systems [243,261]. Dendrimers are better able to take advantage of the EPR effect due to their dimensions, and the branched structure made it possible to conjugate multiple DOX molecules within one dendrimer. PEG chains were used as protective decoration. When tested on tumor-bearing mice, the tumor growth rate was reduced, while weight loss was negligible. Even more recently, Duncan et al. used reversible addition-fragmentation chain transfer (RAFT) polymerization to synthesize an amphiphilic HPMA-gemcitabine (GEM) polymer-drug conjugate [540]. A GEM-containing monomer, N-methacryloyl- glycylphenylalanylleucylglycyl-gemcitabine (MA-GFLG-GEM), and an HPMA monomer were polymerized on opposite ends of a GFLG-bridged 4-(phenylcarbonothioylthio) pentanoate-terminated compound, CTA-CFLG-CTA. This resulting amphiphile was then fabricated into micelles. The anticancer drug, GEM, was protected against degradation by being located in the micellar core, and premature release was minimized due to the covalent bonding. In release tests, the presence of papain, a protease, was used to simulate the degradation of the GFLG links by cathepsin B. Nearly 100% of the payload was released in four hours compared to negligible release in the absence of papain. However, although the copolymer was designed to be excreted via renal exclusion after digestion by cathepsin B, there was a significant accumulation of it in the kidneys 36 h after injection in mice.

#### 3.3.6. Enzyme-Responsive DDSs: Limitations and Remaining Issues

Though a significant amount of work has been conducted in the development of enzyme-responsive DDSs, several issues remain that limit clinical application. First, enzyme dysregulation is distinct in tumors with different types and stages, and some enzymes are upregulated in other disease processes [559]. Thus, personalization of the DDS to the specific tumor subtype and stage, as well as accounting for other diseases or physical conditions of the patient, is required. Second, the liver contains a large concentration of hydrolases and has evolved to capture and clear foreign materials and toxins. Enzyme-mediated hydrolysis in the liver leads to undesired payload release and systemic toxicity. Therefore, enzyme-responsive DDSs systems based on hydrolases need to be further optimized to include a protective layer to limit liver accumulation and hydrolysis [157].

### 3.4. Thermo-Responsive DDSs

There are three primary techniques used to endow DDSs with thermo-responsive behavior (Table 5)—the use of thermo-sensitive polymers or lipids in their construction, the inclusion of thermo-responsive attractive intermolecular interactions (binding) in the DDS structure, and the inclusion of thermally labile covalent bonds in the DDS building blocks. However, while tissue hyperthermia results from many inflammatory diseases and has also been observed in cancer tumors [439,440], the temperature difference between the tumor and the normal physiological conditions is typically not sufficient to be reliably used as a stimulus. Thus, techniques to induce local hyperthermia are often used in conjunction with thermo-responsive DDSs [104]. Hyperthermia treatment has also been demonstrated to increase the accumulation of DDSs at the treatment site and increase cancer cell sensitivity to both radiotherapy and chemotherapy, thereby enhancing the efficacy of the treatment regime [15,560].

**Table 5:** Summary of thermo-responsive mechanisms used in stimuli-responsive drug delivery systems.

Mechanism	Stimuli-Induced Response	Reference
LCST Polymers	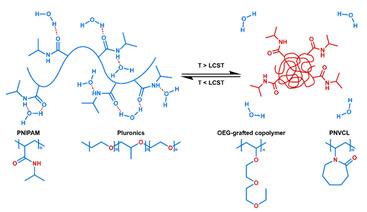	[21] ^b,c,^* [204] ^c,^*** [464] ^c,^*** [468] ^a,b,c,^*** [561] ^c,^** [562] ^a,b,c,^** [563] ^c,^*** [564] ^c,^** [565] ^c,^* (PNIPAM)
		[52] ^b,c,^** (Pluronics)
		[54] ^b,c,^** [566] ^a,c^ [567] ^a,b,c,^*** (OEG-grafted copolymer)
		[568] ^b,c,^* (PNVCL)
UCST polymers	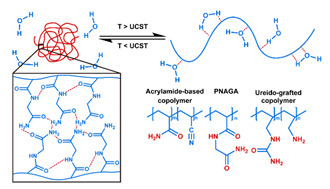	[569] ^b,c,^** [570] ^a,b,c,^* [571] ^b,c,^*** [572] ^a,b,c,^** acrylamide-based copolymer)
		[279] ^b,c,^** (PNAGA)
		[573] ^c^ (ureido-grafted copolymer)
Thermo-responsive peptides	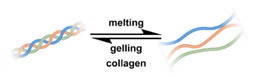	[574] ^c,^*
	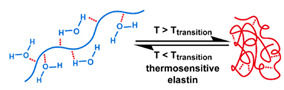	[575] ^a,b,c,^**
	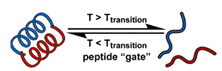	[18] ^a,b,c,^* [576] ^c,^***
Lipid-related transitions	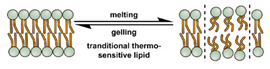	[105] ^c,^** [279] ^b,c,^** [577] [578] ^a,b,c,^*** [579] [580] ^c^
	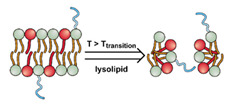	[105] ^c,^** [106] ^a,c,^* [581] ^c,^* [582] ^c,^** [583] ^a,c^
Gas disruption	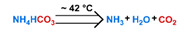	[116] ^b,c,^** [584] ^b,c,^** [585] ^a,c,^**
Thermo-sensitive binding	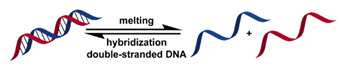	[586] ^c,^*** [587] ^b,c,^*** [588] ^b,c,^**
	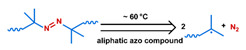	[311] ^a,b,c,^** [589] ^a,b,c,^*** [590] ^b,c,^***
	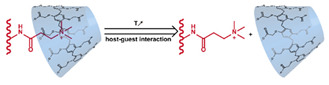	[71] ^b,c,^***
Thermo-weakened adsorption	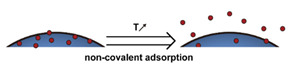	[238] ^a,b,c,^** [591] ^b,c,^** [237] ^a,b,c,^** [252] ^a,b,c,^**

^a^ Evaluation included in vivo tests on mice.; ^b^ Evaluation included in vitro tests on cells.; ^c^ Small molecules, such as antitumor drugs and dyes, were used as payloads.; ^d^ Large molecules, such as proteins and plasmid DNA, were used as payloads.; * Premature payload leakage was >20%.; ** Premature payload leakage was between 10% and 20%.; *** Premature payload leakage was ≤10%.

Thermo-responsive polymers are the most studied technique to endow DDSs with thermo-responsive behavior. Most systems utilize polymers with lower critical solution temperatures (LCST) or upper critical solution temperatures (UCST) a few tens of degrees above normal physiological temperature. Extensive work has been conducted using LCST polymers such as poly(N-isopropylacrylamide) (PNIPAM), poly(N-vinyl caprolactam) (PNVCL), OEG-graft polymers, and Pluronics [568,592,593,594,595]. At temperatures below the LCST, the polymer is hydrated in aqueous solution and forms hydrogen bonds with water molecules. When the temperature is elevated above the LCST, the hydrogen bonds are broken, and the resulting desolvation results in the polymer shrinking to form a hydrophobic globule, expelling the payload from the system. The transition temperature for these polymers can be tailored by adjusting the composition and degree of polymerization [596]. In contrast, UCST polymers preferentially form hydrogen bonds with water above a critical temperature and hydrogen bonds intramolecularly or intermolecularly with other polymer chain segments at lower temperatures. As the temperature is increased above the critical temperature, the polymer is solvated, resulting in swelling that increases the diffusion of payload from the DDS. For biomedical applications, commonly used UCST polymers include acrylamide-based copolymers such as poly(N-acryloylglycinamide) (PNAGA) and ureido-grafted copolymers [573,597]. Though less studied than LCST polymers, the unique thermally induced solubilization in UCST polymers has attracted significant interest more recently [597,598]. In addition to synthetic polymers, the use of thermosensitive biopolymers in thermo-responsive DDSs has gained interest in recent years. Precisely defining the amino acid sequence and chain length can be used to tailor these materials so that they exhibit sharp thermally induced transitions. They also possess excellent biodegradability and biocompatibility [599]. Although this field is still its infancy, several thermosensitive peptides have been reported and used to develop thermo-responsive liposome-based DDSs [575,576,600]. In these peptides, hydrogen bonds break above a critical temperature leading to conformational changes resulting in rapid destabilization of lipid membranes. Double-stranded oligonucleotides (dsDNA) are another class of thermosensitive biopolymers. Above their “melting” temperature, the hydrogen bonds between the double strands break, leading to their separation [586]. The transition point can be tailored by changing the length of dsDNA.

The weakening of intermolecular interactions at elevated temperatures has also been explored in thermo-responsive DDSs. In supramolecules, the host–guest interaction is weakened by elevated temperatures [71], disrupting structures based on them, and releasing payloads. Liposomes have been fabricated from lipids possessing a gel–fluid transition temperature (melting temperature, T_m_) close to physiological temperature [105,106]. Grain boundaries between gel and fluid phases serve as cracks, enhancing the permeability of the membrane. Lysolipids stabilize these grain boundaries due to their smaller intrinsic curvature. PEGylated lipids can further enhance lipid membrane permeability by forming and stabilizing pores [105,580].

Thermal cleavage of covalent bonds has also been explored as a mechanism to endow DDSs with thermo-sensitivity. Compared to physical changes such as desolvation, swelling, and peptide conformational changes, the breaking of covalent bonds can be more reliably controlled, better avoiding the premature release of the payload. Thus, while not as extensively studied as other approaches, application of the thermal-labile covalent linkages, specifically, the azo bond, is of significant interest for drug delivery [331,589,590]. DDSs have also been developed that release the payload when an inorganic compound undergoes thermal decomposition [116].

#### 3.4.1. Thermo-Responsive Micelles

The primary mechanisms driving thermo-responsive release from micelle-based DDSs is the collapse of the hydrophilic layer and dissociation of the micelle. When the hydrophilic layer collapses, the payload carried in this region of the micelle is pushed out into the medium. If the payload is carried in the hydrophobic core of the micelle, hydrophilic layer collapse reduces the diffusion barrier, allowing the release of the payload. Collapse of the hydrophilic layer results from the deswelling that occurs for LCST polymers when the temperature is elevated above the LCST. The low LCST and high CMC of Pluronic polymers limit their application in thermo-responsive DDSs, as they would respond to normal body temperature. Guo et al. overcame this obstacle by introducing a PLA block to Pluronic F127, reducing the CMC, and increasing the LCST [52]. They were able to increase the LCST of the Pluronic F127-PLA copolymer to between 35 and 45 °C by increasing the length of the PLA blocks. They attributed this behavior to an increase in the attractive interactions between the PLA blocks and the F127 blocks as the PLA blocks grew in length. This increased intermolecular affinity in the core required more energy to induce micelle dissociation. At temperatures below the LCST of the copolymer, both the poly(ethylene oxide) (PEO) and poly(propylene oxide) (PPO) segments of the pluronic were hydrated. As a result, the copolymer phase segregated, forming a micellar structure with the F127 block, forming a hydrophilic shell around the hydrophobic PLA core. At temperatures above the LCST, hydrogen bonding in the PPO segments was reduced, and they dehydrated, resulting in the collapse of the structure. The system was demonstrated to preserve the payload at 37 °C and release it when the temperature was raised to 40 °C. HeLa cell assays demonstrated that the system had a higher IC50 (1.45 μg/mL) than free DOX (0.99 μg/mL) at 37 °C, but lower IC50 (0.28 μg/mL) than free DOX (0.41 μg/mL) at 40 °C. A similar enhancement of cytotoxicity was observed in the DOX-loaded poly(γ-2-[2-(2-methoyethoxy)ethoxy] ethoxy-ε-caprolactone)- b-poly(γ-octyloxy-ε-caprolactone) micelles studied by Cheng et al. [54]. They attributed the effect to the increased cell internalization enabled by the increased hydrophobicity of the micelle. In contrast to the increased hydrophobicity exhibited by LCST polymers, UCST polymers exhibit reduced hydrophobicity at elevated temperatures, which can induce dissociation of UCST polymer-based micelles and release of the payloads [569]. Li et al. prepared a polymer with an UCST in the physiological temperature range by conjugating poly(acrylamide-co-acrylonitrile) and PEG [570]. Enhanced cytotoxicity of the drug-loaded micelle was demonstrated via cancer cell assays. However, the transition temperature range of the system was wide (4 to 44 °C). As a result, the micelle exhibited significant leakage at 37 °C.

#### 3.4.2. Thermo-Responsive Liposomes

Several lipids have melting points close to physiological temperature and are, therefore, interesting candidates for the preparation of thermo-responsive liposomes. Mills et al. demonstrated this behavior in a dipalmitoylphosphatidylcholine (DPPC)-based liposome [105]. A peak in permeability was observed as the system approached the Tm. However, as the temperature was increased beyond the Tm, the grain boundaries disappeared (the system was fully “molten”). The permeability dropped and then began to rise again with temperature due to the increased mobility of the lipid. Introducing other lipids such as distearoyl phosphocholine (DSPC) and hydrogenated soy phosphocholine (HSPC) to the liposome formulation can enhance the thermal sensitivity by reducing the packing efficiency of the combined system, increasing the membrane permeability [104]. This effect was demonstrated by Needham et al., who reported enhanced DOX release in a system that contained both DPPC and HSPC [106]. Insertion of metal nanoparticles into lipid membranes also affects membrane integrity [577]. Qui et al. used this behavior to control the effective Tm of liposomes through the addition of 6 nm Fe_3_O_4_ nanoparticles to a lecithin-based liposome [580]. The inclusion of Fe_3_O_4_ nanoparticles also confers additional functionality, such as a response to magnetic fields and a photothermal response; both responses are reviewed below [578,579].

While the appearance of grain boundaries during melting enhances permeability, the effect on payload release rates is minor. In contrast, a more substantial permeability enhancement can be achieved through the addition of lysolipids, which possess higher intrinsic curvature than ordinary lipids, to create stable pores in the membrane at elevated temperatures. Needham et al. included the lysolipid monopalmitoylphosphatidylcholine (MSPC) in a DPPC liposome [581]. When the temperature of the system was at the Tm, an extremely rapid release of the payload was observed. According to the authors, the enhanced lipid mobility at the melting point allows the lysolipid to form stable pores (~10 nm in diameter) in the membrane. These pores are stabilized by micellar structures formed by MSPC, which connected the liposome core and the outer medium, (Figure 2a(ii)). They also reported that the inclusion of PEGylated lipid could further stabilize the lysolipid-formed pores, due to its micelle forming behavior, and further increases the release rates. Interestingly, Mills et al. found that the improved release rate from the formation of micelle-stabilized pores was reduced as the temperature was increased [105]. They attributed this behavior to the loss of pores when the membrane was fully molten.

The interaction between the thermo-responsive liposomes and the physiological environment may alter their thermal responsiveness. Serum proteins adsorbed on lipid membranes can slightly enhance stability [601]. Proteins can insert into the grain boundaries or directly penetrate the lipid membrane, enhancing the permeability and thermo-responsiveness of the liposome. In addition to proteins, Hossann et al. found that exchange with serum cholesterol could also increase lipid membrane permeability [601]. While a PEG coating is considered an effective approach to avoid interaction between serum proteins and nanoparticle DDSs [366], several studies have demonstrated enhanced drug leakage in the presence of serum proteins or cholesterol for liposomes with PEG decoration, especially for lysolipid-incorporating liposomes [582,601,602]. The loss of lysolipids through extraction or exchange processes via protein interactions can also reduce the thermo-sensitivity of liposomes, as demonstrated in Banno et al.’s work [582]. An obvious reduction in DOX release rate was observed after the lysolipid-containing liposomes were incubated in mouse blood for 2 h. Thus, further studies are required on the protection of lipid and lysolipid from the serum destabilization effect for these thermo-responsive liposomes.

In addition to lipid phase transitions, the temperature-dependent solubility of some polymers has been leveraged to create thermo-responsive liposome DDSs. Kono et al. conjugated poly[2-(2-ethoxy)ethoxyethyl vinyl ether] (poly(EOEOVE)) to four octadecyl vinyl ether (OD) molecules [567]. The resulting molecule poly(EOEOVE)-OD4 exhibits an LCST of around 40 °C. At temperatures below the LCST, the system formed stable liposomes. However, at temperatures above the LCST, hydrogen bonding was reduced, and the now hydrophobic EOEOVE chains dehydrated and inserted themselves into the bilayer, significantly destabilizing the structure. At 37 °C, the system exhibited minor drug leakage, <5%. As the temperature increased, the rate of drug release from the system dramatically increased, and complete drug release was obtained at 47 ℃ in 5 min. The addition of PEG-modified lipids to the formulation effectively enhanced the circulation life of the DDS. Over 40% of the injected dose was still circulating in the blood 6 h after administration.

Thermo-responsive peptides have also been incorporated in liposomes to create a new class of thermo-responsive DDSs. Al-Ahmady et al. included a leucine zipper sequence peptide in the formulation of a DPPC-based liposome [18]. Below a critical temperature, this peptide exhibits an α-helix conformation with hydrophobic residues exposed to one side. Neighboring peptides wrap around each other to form a superhelix coiled-coil dimer aggregate. Above the critical temperature, the peptides unfold, and the aggregates dissociate from each other, creating cracks in the lipid membranes that are still in the gel state. The enhanced permeability releases the payload. The thermal sensitivity of this system was lower than that of lysolipid-containing liposomes in release tests. However, an extended circulation life and improved tumor accumulation were observed in vivo.

Sung’s group explored a unique method to endow liposomes with thermo-responsiveness [116,584,585]. In their approach, NH_4_HCO_3_ was incorporated into the liposome core. At 42 °C, decomposition of the salt releases a significant amount of CO_2_. The pressure generated disrupts the liposomes and releases the payload within one minute. The system was tested on human lung cancer (H460) tumor-bearing mice. Compared with control liposomes that did not contain NH_4_HCO_3_, there was a higher accumulation of DOX in H460 tumors and lower retention in the liver when local hyperthermia was applied to the tumor site. Additionally, the CO_2_ bubbles generated enhanced ultrasound imaging.

#### 3.4.3. Thermo-Responsive Polymeric Nanoparticles

It is difficult to induce morphology changes in a compact polymeric nanoparticle via the level of hyperthermia in typical tumors, as the required temperature increase is often several tens of degrees. However, remote heating techniques via the incorporation of magnetic nanoparticles or photothermal agents have been used in these systems. These approaches will be discussed in Section 3.5.4 and Section 3.6.3. In contrast, the temperature elevation required to disrupt dendrimers and nanogels is milder. Systems based on polymer hydrophobicity changes and conformational changes of peptides have been explored in dendrimer-based DDSs. In Zhao et al.’s work, PNIPAM was conjugated on the branches of PAMAM dendrimer to produce a DDS that changed hydrophobicity with temperature [561]. At temperatures below the LCST, the indomethacin payload could efficiently diffuse through the hydrated PNIPAM and be released to the surroundings. At temperatures above the LCST, the PNIPAM shrank to dehydrated globules, and indomethacin diffusion was limited. The “low-temperature release” behavior exhibited by this system does not apply to the treatment of tumors via hyperthermia-stimulated release. In contrast, the system developed by Kojima et al. and based on the temperature-induced conformational change of collagen-mimic peptide conjugated to PAMAM dendrimers initiated release at physiological temperatures [574]. At 4 °C, the triple helix structure of collagen-mimic peptide resulted in a dense network that limited the release of the payload, rose bengal (RB). At physiological temperatures, the melting of the collagen-mimic peptide led to a rapid release of ~100% of the loaded RB in 5 h. Unfortunately, most likely due to the thin protective layers, drug leakage was significant for both the system explored by Zhao et al. and the system studied by Kojima et al. While thermo-responsive dendrimers are less studied than other thermo-responsive systems, they merit further study due to their small dimensions. They are generally 5–20 nm in diameter, which is advantageous for extending circulation times and taking advantage of the EPR effect, as described in Section 2.6.1 [603].

Thermally responsive nanogels can be established by crosslinking a thermosensitive polymer. For example, in Wang et al.’s work, a chitosan-PNIPAM network was established by copolymerizing chitosan, NIPAM monomer, and a crosslinker, methylene bisacrylamide (MBA) [562]. At temperatures above its LCST, dehydration of PNIPAM led to shrinkage of the nanogel, expelling of the payload, PTX, and the exposure of the chitosan to the surface of the nanogel. The reduced volume increased hydrophobicity, and positively charged chitosan surface significantly enhanced the internalization of the nanogels in human hepatocarcinoma (SMMC-7721) cells, compared with the nanogel at temperatures below the LCST. However, the difference was reduced at longer incubation times. This effect was attributed to the system reaching the internalization capacity of the cells [604]. Salinas et al. developed a pH-temperature dual-responsive nanogel by copolymerizing NIPAM and N-acryloyl-L-proline (A-Pro-OH) in the presence of the crosslinker MBA [464]. Above the LCST, the nanogel shrank, leading to a rapid release of the payload, NB. The pH-dependent protonation of carboxyl groups on proline further enhanced the hydrophobicity of the nanogel and resulted in a 1.5 °C reduction in the LCST of the system. At pH 7.4 and room temperature, less than 10% of the loaded NB was released in 24 h. Lowering the pH to 5.2 resulted in around 35% of the payload being released, while simultaneously reducing the pH to 5.2 and raising the temperature to 43 °C resulted in over 80% of the payload being released in 10 h.

When LCST polymers are used, the thermo-responsive nanogel would be expected to shrink when the temperature is increased to above the transition temperature. However, in He et al.’s work, a nanogel composed of poly[2-(pyridine-2-yldisulfanyl) ethyl acrylate-co-poly(ethylene glycol) methacrylate-co-N-isopropyl methacrylamide] crosslinked via disulfide bonds, exhibited a roughly 10× increase in diameter when the temperature was increased above the transition temperature [468]. They reported that the transition temperature could be facilely tuned from 30.5 to 47 ℃ by adjusting the crosslinking density. Furthermore, because the crosslinking was established by disulfide bonds and pyridine was present in the backbone of the copolymer, the nanogel also possessed pH and redox responses. At pH 5 or in 10 mM DTT, the nanogel also exhibited extreme expansion. Though the expansion mechanism is yet to be determined, the authors suggested this unusual dimension change could provide a new strategy for endosome escape.

#### 3.4.4. Thermo-Responsive Porous Inorganic Nanoparticles

A thermosensitive polymer can also be attached to inorganic nanocarriers. However, systems that use LCST polymers exhibit unexpected behavior when used in this fashion. For example, in You et al.’s work, PNIPAM was attached to an MSN-based DDS as a gatekeeper [563]. At lower temperatures, the polymer expands in a random coil with enough free volume to allow payload release. At temperatures above the LCST of the polymer, it shrank into a globular state, reducing free volume and retarding release. Similarly, “low-temperature release” was reported by Nagata et al. for PNIPAM-modified MOF [564]. In contrast, in Zhou et al. and Brunella et al.’s studies, PNIPAM prevented the release of the hydrophobic payload, ibuprofen (IBU), from an MSN-based DDS by forming hydrogen bonds with the payload below the LCST, and releasing the drug when the bonds were broken at elevated temperatures [204,565]. The behavior of LCST polymers in these systems suggests that UCST polymers may be a better choice of thermo-responsive gatekeepers. Hei et al. conjugated poly(acrylamide-co-acrylonitrile) onto an MSN via a disulfide bond [571]. Under the UCST, the polymer collapsed into globular morphology and blocked the pores on MSN, while above the transition temperature, it converted to a random coil state and released the payload. The drug leakage under 25 ℃ was less than 5%, while above UCST, over 50% payload was released in 7 h. The release exhibited an “on-off” pattern, i.e., the release was suspended as the temperature dropped below the transition point. Additionally, with the addition of DTT, over 80% payload was released in 24 h via breakage of the disulfide bond. There are still drawbacks to this system. Dehydration of the thermosensitive polymer led to an obvious aggregation of the MSN at temperatures below the UCST, which is undesirable for the exploitation of the EPR effect. In addition, the transition temperature requires further optimization, given the significant drug leakage that Hei et al. reported at 37 ℃.

The thermo-responsiveness of dsDNA makes it an attractive material for use as a thermo-responsive gatekeeper. In Schlossbauer et al.’s work, a protein-based macromolecular gatekeeper was conjugated to a DNA strand; the system was then coupled with the complementary DNA strand attached to the MSN surface [586]. As the temperature was increased, hydrogen bonding between the two strands was weakened, allowing the dsDNA to dissociate, releasing the gatekeeper, and allowing the cargo to be released. The response temperature could be tailored by changing the DNA strand length. In Chen et al.’s work, a pair of acetylene-terminated self-complementary DNA strands were directly used as the gatekeeper by anchoring them in the pore of an azide-functionalized MSN [587]. Below the T_m_, the DNA gatekeeper prevented drug release with minimal leakage. At 50 ℃, dissociation of the couple opened the pores, leading to ~95% of the cargo, RhB, being released in 4 h. To achieve remote control of the response, Chang et al. embedded gold nanorods (AuNR) in their MSN and then conjugated one of the strands of a dsDNA to the MSN [588]. The temperature increases when the system is irradiated with NIR wavelength light due to the photothermal effect. Under these conditions, the dsDNA dissociated, and the unanchored strand left the DDS, resulting in the release of the DOX payload through the opened pores. In the cell assay, unirradiated DDSs exhibited slight cytotoxicity, while irradiation for 5 min resulted in higher toxicity than free DOX. Additionally, double-stranded siRNA was attached to the MSN via the same approach that was used for the dsDNA, and successful siRNA delivery was observed in vitro when the system was irradiated.

The host–guest intermolecular affinity in supramolecular pairs is also weakened as the temperature is increased, suggesting their use as gatekeepers in thermo-responsive MSN and MOF DDSs. For example, in the work of Yang’s team, a supramolecular pair consisting of a CP5 ring and QAS stalk were decorated on the surface of UiO-66 MOF [71,220]. The cargo, 5-fluorouracil (5-Fu), was well-preserved with negligible leakage at physiological temperature. At 60 ℃, drug release was completed in ~8 h as CP5 was removed. Additionally, competition from divalent metal ions and protonation of the CP5 under pH 4 also effectively weakened the host-guest affinity and induced drug release.

In addition to physical transitions, thermo-labile bonds have been applied to thermo-responsive MSN and MOF DDSs. In Lei et al.’s work, the β-CD gatekeeper was attached to the MSN surface via an azo linker [331]. An RGD ligand, an MMP-2 substrate peptide, and a PEG chain were sequentially linked to the β-CD. After the DDS extravasated into tumor ECM, the presence of MMP-2 cleaved the peptide, releasing the protective PEG layer and exposing the RGD ligand. The RGD ligand was then able to enhance endocytosis of the nanocarrier. Finally, the payload was released when the application of local hyperthermia cleaved the azo linker releasing the β-CD gatekeeper. The photothermal agent indocyanine green (ICG) was also encapsulated in the MSN. Under NIR laser, the drug release was more rapid than that under 60 ℃ hyperthermia. In the cell assay to 4T1 cells and human embryonic kidney normal (293T) cells, the high fluorescence signal from the DDS in 4T1 cells confirmed the targeting efficiency of the MMP-2 substrate and RGD ligand. With NIR applied, the DOX-loaded DDS exhibited higher cytotoxicity than free DOX with the same amount of ICG added and NIR applied. It should be noted that though the liver accumulation of the system was low, relatively high accumulation in the lungs was observed, which could be attributed to the sequestration of the 30–80 nm particles in the pulmonary capillaries [605,606].

#### 3.4.5. Thermo-Responsive Non-Encapsulated DDDs

Elevated temperatures reduce attractive forces associated with noncovalent bonding. It can also enhance the dissolution and diffusion rates of a drug from the DDS and increase solubility in the release medium, limiting reabsorption by the DDS. Jiang et al. engineered a MOF DDS, which exhibited a strong affinity to diclofenac sodium (DS) via π-π interactions [591]. High loading capacity of 41.7% by weight and leakage below 30% over 24 h at physiological temperatures were observed in the absence of a protective shell. Meanwhile, at 45 °C, absorption equilibrium shifted, and more than 60% of the adsorbed drug was released in 24 h. In contrast to gatekeeper systems, Chen et al. and Tao et al. both evaluated black phosphorus (BP) nanosheet-based DDSs [237,252]. The large surface area and negative zeta potential enabled an extremely high 950% loading capacity for DOX, with leakage of less than 10% in a neutral buffer. BP is a narrow bandgap semiconductor material and can efficiently convert light to heat when irradiated at NIR wavelengths and can generate ROS from ground-state oxygen when irradiated at 660 nm [607,608]. Thus, when irradiated at 808 nm (0.8 W/cm^2^), over 80% of the loaded DOX was released in 20 min. Chen et al. found that in cell assays, the viability of 4T1 cells incubated with unloaded BP was over 95%, but was only 30% when the cells were incubated with DOX-loaded BP and irradiated with an 808 nm laser. When the cells were irradiated with both an 808 nm and a 660 nm (0.015 W/cm^2^) source, ROS was also generated, and the cell viability dropped below 5% [237]. Tao et al. evaluated a system that included a protective PEG coating attached via electrostatic interactions. In their studies, the target cells, in vitro HeLa and in vivo HeLa tumor-bearing mice, were pretreated with chloroquine (CQ), reducing the acidification of endosomes and protecting the BP from degradation in the lysosomes. They found that the antitumor efficacy was significantly higher with the CQ pretreatment [252].

While more limited, thermally cleavable covalent bonds have also been explored as response enablers for thermo-responsive DDSs not based on encapsulation. Temperature elevation can induce the cleavage of gold-sulfur bonds, azo bonds, and the adduct of imine base and isocyanate [590,609,610]. However, to date, only azo bond cleavage has been utilized in thermo-responsive DDSs. Chen et al. connected DOX to poly(ethylene glycol)-blocked-poly(L-lysine) (PEG-b-PLL) via an azo linkage [589]. The copolymer was then coated onto AuNR, forming a nanocomposite. Under NIR irradiation, the photothermal effect exhibited by the AuNR induced cleavage of the azo bond and released the payload.

#### 3.4.6. Thermo-Responsive DDSs: Limitations and Remaining Issues

The primary limitation in the development of thermo-responsive DDSs is the difficulty of controlling the response temperature of many materials. It is critical that the DDS does not respond to physiological conditions to limit the premature release of the payloads [15]. Minimizing premature release requires adjusting the target response temperature to several or tens of degrees higher than physiological temperature, requiring the application of external heating. In most cases, the response temperature is not well defined, and instead, a gradual change in the release behavior is observed as the temperature is increased, making precise control over release temperature impossible. Furthermore, the tumor temperature is not always higher than other tissues; in some studies, tumors have an even lower temperature than surrounding tissues [611]. Possible “off-target” effects can also arise due to the presence of inflammatory tissues, which can also have a relatively high temperature. In fact, the subtle environmental differences between tumors and normal tissue and the variations in these differences between tumor types can result in “off-targeting” issues with many DDSs that rely solely on endogenous stimuli. This issue has driven the development of DDSs that exploit exogenous stimuli.

### 3.5. Magneto-Responsive DDSs

Magnetic fields can be used as non-intrusive and remotely applied exogenous stimuli. There is a history of the use of magnetic nanoparticles (MNPs) to enhance contrast in magnetic resonance imaging (MRI) of tumors [612]. Magnetic nanoparticles have also been used to guide nanocarriers to a tumor site via static magnetic fields and to generate local hyperthermia via alternating magnetic fields (AMF). Static fields are applied externally or via magnets implanted during surgery. The accumulation of magnetically responsive DDSs at the tumor site enhances their cellular uptake [613,614]. One issue is the proper design of the field geometry and strength to avoid the accumulation of the DDS in “off-target” locations [615]. In contrast to the accumulation observed in static fields, magneto-responsive nanoparticles exhibit an enhanced oscillatory motion in an AMF (Table 6). High-frequency AMF can transfer energy to the DDS, producing heat and vibration, which can enhance diffusion and dissolution of the payloads and, if significant and long enough in duration, disrupt the structure of the nanocarrier [213,616]. Low-frequency AMF typically does not induce significant heat in the DDSs but can promote release by enhancing the diffusion of the drug from the DDS into the release medium [222].

**Table 6:** Summary of magneto-responsive mechanisms used in stimuli-responsive drug delivery systems.

Mechanism	Stimuli-Induced Response	Reference
Alternating magnetic field	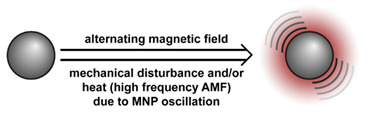	[111] ^c^ [213] ^b,c^ [590] ^b,c,^*** [614] ^b,c,^** [616] ^a,b,c,^*** [617] ^c,^*** [618] ^c,^*** [619] ^c,^*** [620] ^b,d,^** [621] ^b,c,^** [622] ^b,c,^** [623] ^c,^*** [624] ^b,c,^*** [625] ^b,c,^*** [626], [627] ^b,c,^*** [628] ^b,c,^*** [629] ^b,c,^* [630] ^a,c^ [631] ^c^ [632] ^c,^* (high frequency AMF)
		[222] ^c,^* (low-frequency AMF)
Static magnetic field	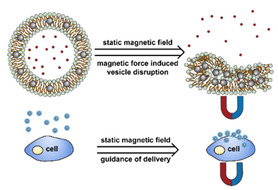	[67] ^c,^*** (static magnetic field stimulated release)
		[633] ^b,c^ [634] ^a,b,c^ [635] ^a,b,c^ [636] ^b,d^ [637] ^b,d^ [638] ^b,d^ (static magnetic field guidance delivery)

^a^ Evaluation included in vivo tests on mice.; ^b^ Evaluation included in vitro tests on cells.; ^c^ Small molecules, such as antitumor drugs and dyes, were used as payloads.; ^d^ Large molecules, such as proteins and plasmid DNA, were used as payloads.; * Premature payload leakage was >20%.; ** Premature payload leakage was between 10% and 20%.; *** Premature payload leakage was ≤10%.

The size of MNPs used in DDSs should be small enough (theoretically <25 nm in diameter for Fe-based materials) to achieve superparamagnetism, so that after the magnetic field is removed, the remanence is negligible and will not lead to nanomaterials agglomeration [639]. This limitation, together with the fact that most organic materials are non-magnetic, means that most magneto-responsive DDSs are composite systems in which comparatively small MNPs are attached to a non-magnetic DDS to endow it with magnetically responsive behavior [615]. There are three primary strategies used to incorporate MNPs in DDSs. First, MNPs can be embedded or encapsulated in the nanocarrier during DDS fabrication. Arias et al. has reviewed the encapsulation of MNPs in polymeric and inorganic materials [640]. MNPs can be also be encapsulated in the aqueous core of liposomes, embedded in their lipid membrane, or attached on their surface [15]. Second, MNPs can be synthesized in situ after the DDS is formulated. For example, in Guo et al.’s work, Fe_3_O_4_ nanoparticles were synthesized in situ on the surface of polydopamine via Fe^2+^ and Fe^3+^ ions [641]. Third, the surface of a DDS can be decorated with functionalized magnetic nanoparticles via covalent bonds. In Xia et al.’s work, alkene-terminated Fe_3_O_4_ was attached to the MSN surface via microwave-induced hydrosilylation [642].

#### 3.5.1. Magnetic Field-Guided Drug Delivery

Static magnetic fields can be used to guide magnetic nanoparticles and DDSs that include embedded or attached magnetic nanoparticles to tumor sites, enhancing drug delivery efficiency. This strategy can be applied to virtually any kind of DDS, as guidance is achieved solely through the interaction of the MNPs with the static field. Therefore, recent advances magneto-guidance are reviewed in this section, regardless of the DDS type. DDSs utilizing AMF are reviewed in the subsections below corresponding to the respective DDS.

In cell assays, when a static magnetic field is applied to attract magneto-responsive drug-containing nanocarriers to the cells, improvement of both cell uptake and cytotoxicity is observed. Zhang et al. demonstrated this behavior for DOX-loaded MSN that encapsulated 25 nm Fe_3_O_4_ nanoparticles [633]. Cell viability was reduced from ~45% without the magnetic field to ~30% with the magnetic field in HepG2 in vitro studies. In Hua et al.’s study of the antitumor drug 1,3-bis(2-chloroethyl)-1-nitrosourea (BCNU) conjugated to carboxyl functionalized Fe_3_O_4_ nanoparticles via an amide group, the IC50 was reduced from 18.9 to 9.5 μg/mL in the presence of a magnetic guide [634]. Zhang et al. reported a similar reduction in IC50 for silica-coated Fe_3_O_4_ MNPs for DOX delivery. The IC50 dropped from 0.25 to 0.12 μg/mL with the application of the static magnetic field during in vitro tests on human hepatoma (QGY-7703) cells. They reported a threefold higher accumulation of the DDS at the tumor site during in vivo testing when the static magnetic field was applied. Hua et al. reported similar improvements for the delivery of BCNU from BCNU-conjugated Fe_3_O_4_ MNPs in rats [634]. This technique has also been demonstrated to enhance gene transfection efficiency. Prijic et al. and Al-Deen et al. both reported roughly twice the protein expression when pDNA-loaded DDSs were used in the presence of a static magnetic field [636,637].

#### 3.5.2. Magneto-Responsive Micelles

One facile strategy for enabling AMF-triggered drug release is to utilize magneto-responsive nanoparticles to induce a temperature elevation sufficient to cross the T_g_ or the T_m_ of the polymers used in the micelle. Glover et al. explored release based on the melting of the PCL core in a system composed of PEG-PCL block copolymer and co-encapsulated Fe_3_O_4_ MNP and DOX [632]. Kim et al. conjugated PEG-PLA block copolymers to a carboxyl-functionalized Fe_3_O_4_ MNP and encapsulated DOX [629]. In both cases, AMF served to melt the hydrophobic core (PCL or PLA), destabilizing the system and releasing the payload.

Thermo-responsive polymers can also be combined with MNPs to enable magneto-responsive behavior. Kim et al. explored this technique using poly(N-isopropylacrylamide-co-acrylamide)-block-poly(e-caprolactone) (P(NIPAAm-co-AAM)-b-PCL)-based micelles that co-encapsulated Fe_3_O_4_ MNPs and DOX [628]. Exposure to AMF (77 Oe) increased the temperature to ~43 °C, inducing shrinkage of the P(NIPAAm-AAM) shell due to dehydration of this block, expelling the loaded DOX from the system. Interestingly, they reported that the release exhibited an “on-off” behavior in which release was initiated by applying an AMF and stopped with the AMF was removed. Exposure to high energies (140 Oe AMF) increased the temperature to 55 °C, resulting in the melting of the PCL core, destabilization of the micelle, and a burst release of the payload. In contrast to a single copolymer, Huang et al. evaluated blended polymer systems based on Pluronic F127 and varying amounts of PVA for the AMF-stimulated release of ethosuximide (ETX) [617]. Exposure to AMF triggered a temperature increase due to the Fe_3_O_4_ MNP co-encapsulated in the system. The resulting reduction in the hydrogen bonding between the Pluronic and water caused the system to dehydrate. The reduction in hydrogen bonding between the Pluronic and PVA destabilized the micelle, further promoting ETX release. As the fraction of the PVA in the system increased from 0 to 80 wt.%, the response temperature increased from 25.8 to 47.4 °C, and the amount of ETX released by exposure to AMF decreased.

#### 3.5.3. Magneto-Responsive Liposomes

MNPs can be either encapsulated in the aqueous core or embedded in the lipid membrane of liposomes to impart magneto-responsive behavior to them. When located in the core, the heat produced during the application of AMF induces a phase transition of the lipid leading to thermo-responsive release. Pradhan et al. co-encapsulated MNPs and DOX in the core of DPPC-based liposomes [614]. Additionally, an FA-conjugated lipid was used in the liposome to enhance targeting. In cell assays, the combination of static magnetic guidance and the FA ligand enhanced internalization of the DDS in human epidermoid carcinoma (KB) cells. After the application of an AMF, the KB cell viability was reduced to less than 10%. In contrast, embedding a hydrophobized MNP in the lipid membrane enables the direct transfer of heat to the bilayer, and the oscillatory motion of the MNP under AMF can mechanically disrupt the membrane. This process is more efficient than the secondary effect of heat-induced phase change that results for MNP encapsulated in a core. Amstad et al. compared hydrophilic MNP encapsulated in an aqueous core and a hydrophobized MNP embedded in the lipid membrane [111]. They reported that significantly more drug, calcein, was released from the system with the MNP embedded in the bilayer. However, they also reported that the hydrophobic surface on the Fe_3_O_4_ particles was not stable, leading to their agglomeration and diffusion out of the bilayer, both of which impaired the magneto-responsiveness of the system. Qiu et al. reported an “on-off” release behavior for their system, which was composed of hydrophobized MNP embedded in the bilayer of lecithin-based liposomes [618]. When AMF was applied, a zero-order release profile was observed, and release from the system when incubated in a heated water bath was less than that observed when the system was exposed to AMF. This behavior suggests that the motion of the MNP in the bilayer promoted cargo release via a mechanism that was not temperature related. When the AMF was removed, the release was suspended, indicating the agitation was non-destructive and reversible. Katagirl et al. developed an “on-off” capable DDS based on the magneto-responsive insertion of poly(EOEOVE) into the bilayer [619]. In their system, OD-conjugated poly(EOEOVE) was incorporated with MNPs in a lecithin-based liposome. Under AMF, the resulting temperature increase induced dehydration of the poly(EOEOVE) and its insertion in the bilayer resulting in 90% of the loaded pyranine being released in one hour. When the AMF was removed, the poly(EOEOVE) rehydrated and left the bilayer, and release stopped. The inclusion of the MNP did not impair the stability of the liposome, and a similar “on-off” mode to that found by Qiu et al. was observed.

Zhou et al. has challenged the previously clear distinction between the use of static magnetic fields for guidance and AMF for stimulated release [67]. In a proof-of-concept study, they reported the use of a static magnetic field to trigger payload release from the vesicles, non-spherical liposomes, with Fe_3_O_4_ MNPs embedded in the shell. They synthesized the amphiphile (AP5-glycol) by attaching five oligomeric glycol chains to one side of pillar[5]arene and five alkyl chains to the other side. An applied static magnetic field forces migration of the MNPs within the bilayer resulting in destabilization of the system and release of the payload. While the vesicle-to-irregular structure transition responsible for the release is unique to vesicles, the study is of interest as it enables magneto-responsive release via low-cost static magnetic fields.

#### 3.5.4. Magneto-Responsive Polymeric Nanoparticles

One technique to impart magneto-responsiveness to solid polymeric nanoparticles leverages the increased permeability of polymers heated above their glass transition temperature. Despite being based on diffusion, this response can be rapid. Hu et al. used this mechanism to develop systems for the delivery of PTX and DOX from PVA nanoparticles [616] and macromolecular pDNA from Janus-structured nanoparticles of poly(styrene allyl alcohol) (PS-PAA) [620]. Both systems contained Fe_3_O_4_ MNPs. Release from these systems was rapid; 10 min AMF exposure was effective in releasing the PTX and DOX from the PVA-based nanoparticles, while a burst release of pDNA was observed after only one minute of exposure in the Janus nanoparticle-based system [630]. Hayashi et al. reported similar results for the release of DOX from polypyrrole (PPy) nanoparticles with co-encapsulated Fe_3_O_4_ MNPs. They reported that 60% of the loaded DOX was released after 20 min of AMF exposure.

MNPs can also be encapsulated in thermo-responsive nanogels to enable magneto-responsive release. Cazares-Cortes et al. co-encapsulated Fe_2_O_3_ MNPs and DOX in oligo(ethylene glycol) methyl ether methacrylate (OEGMA)-based nanogels (MagNanogel) [621]. Application of an AMF induced drug release by heating the system and activating the thermally induced shrinkage of the nanogel. They also compared the release performance of MagNanogel and an MNP-encapsulating compact nanoparticle composed of ethylene glycol dimethyl acrylate (MagMIP) [622]. The DOX was loaded in MagMIP via imprinting polymerization so that under AMF, the DOX could be released via heat-accelerated dissolution. The MagNanogel exhibited higher loading capacity and released a higher fraction of the payload. However, it also exhibited a greater tendency for premature release via leakage through the network. These differences impacted cell assay results. Although the MagNanogel showed higher cytotoxicity than the MagMIP when exposed to an AMF, the control groups without AMF exposure also exhibited cytotoxicity. The drug leakage issue has also been observed in other studies of magnetically responsive nanogels [643,644].

#### 3.5.5. Magneto-Responsive Porous Inorganic Nanoparticles

Thermo-responsive gatekeeper strategies were discussed in Section 3.4.4. These strategies can be activated in MNPs containing inorganic nanoparticles by exposure to an AMF, enabling magneto-responsive inorganic nanoparticles. The MNP itself can also serve as a gatekeeper. In Ruiz-Hernández et al.’s work, DNA-conjugated MNP gatekeepers were immobilized via complementary DNA strands bound to an MSN [623]. The local temperature elevation due to AMF exposure induced dehybridization of the dsDNA, releasing the MNP and uncapping the MSN pores. The fraction of MNPs removed depended on the AMF exposure time, and unreleased MNPs continued to effectively block pores, resulting in an “on-off” behavior for the system. In contrast to the dehybridization of dsDNA, Thomas et al. used a thermally weakened guest–host interaction to create an “on-off” release behavior for an MSN-based DDS [625]. In this system, an Fe_3_O_4_ MNP was encapsulated in a hollow MSN, and Cucurbit[6]uril was bound to amine groups immobilized on the surface. AMF-induced temperature increase uncapped the Cucurbit[6]uril ring, releasing the DOX payload. No leakage of the payload was observed before the AMF was applied, but once applied to the system, a burst release of ~40% DOX was achieved.

Membranes coated on inorganic nanoparticles can be destabilized by vibration and heat generated due to AMF exposure and release payloads. “On-off” behavior has been reported for these systems as well. Hu et al. synthesized an Fe_3_O_4_ shell on the surface of a PVP-coated MSN [213]. A short (30 s) AMF exposure induced small reversible deformations of the shell and allowed the payload to diffuse through the shell. AMF exposure times longer than 120 s ruptured the shell and resulted in a burst release from the system. In contrast to using a magneto-responsive coating, Bringas et al. used a lipid membrane to effectively block drug leakage from MSN that co-encapsulated both MNP and methylene blue (MB) [627]. In the absence of AMF, the MB leakage was less than 10% over 6 h. However, when exposed to AMF, the temperature elevation and mechanical oscillation effectively disrupted the lipid membrane and induced a complete (>95%) release of the cargo in 6 h.

#### 3.5.6. Magneto-Responsive Non-Encapsulated DDDs

AMF can be used to induce drug release from DDSs that are based on adsorption via noncovalent interactions or thermally cleavable covalent bonds. Mechanical oscillation can be used to promote cargo release such as in the work of Fang et al., which used low frequency (20 Hz) AMF to promote the release of 5-Fu adsorbed in a ZIF-90 MOF [222]. While AMF promoted release without inducing an obvious thermal effect, there was significant leakage of the payload from the system, as no protective layer or gatekeeper was used. Griffete et al. used molecular imprinting techniques to enhance the stability of DOX adsorbed on a poly(acrylamide) (PAAM) shell containing a Fe_2_O_3_ MNP [645]. The system exhibited only a slight leakage of the payload in water; however, when AMF was applied, a significant fraction of the loaded DOX was released. Prodrug and conjugate systems can be tethered to MNPs to enable magneto-responsive release such as in the work of Derfus et al. [626]. They utilized the melting of a hybridized dsDNA, one chain attached to the magneto-responsive nanoparticle and the other attached to the payload, to enable the magneto-responsive release of fluorescence dye. The thermal sensitivity of the system was reduced as the length of the dsDNA strand was increased; a higher temperature as a result of higher AMF power was required to stimulate release. This interesting proof of concept study suggests that systems in which several different length dsDNA strands are used might enable dosage control by adjusting the AMF power.

#### 3.5.7. Magneto-Responsive DDSs: Limitations and Remaining Issues

Although remote therapeutic strategies employing magneto-responsive DDSs are elegant and non-intrusive, there are still obstacles limiting their clinical application. Incorporating MNP into thermo-responsive DDSs avoids the aggregation issue that arises when utilizing isolated MNPs. However, encapsulation can lead to a reduction in the magnetization, limiting responsiveness. This effect can be attributed to the addition of non-magnetic materials and the enhancement of the spin disordered effect when MNP is coated by silica [616,627,630,646]. Not only can a high dosage of magnetic materials be toxic, even when they are embedded in nontoxic carriers [647], but the exposure to high frequency (50–400 kHz) AMF may cause peripheral nerve damage and cardiac issues [648]. Furthermore, magnetic therapy is difficult to apply for in the case of metastasis or disseminated tumors. Lastly, the proper set up and localized targeting of an AMF, particularly for deep-seated tumors, can be complicated and expensive. Despite these limitations, magnetically responsive systems are a good option when the tumor location is well defined but in a difficult or dangerous place for surgery [649].

### 3.6. Photo-Responsive DDSs

The use of the exogenous stimulus photoirradiation to activate the release of payload from DDSs is one of the most active fields of research (Table 7). Compared with other stimuli-responsive DDSs, one advantage of photo-responsive DDSs is that release rates are relatively rapid. For instance, in Chen et al. and Han et al.’s studies, dual-responsive systems were investigated [455,650]. While the response to other stimuli, pH or redox, occurred over hours, the photo-triggered release occurred within several minutes, or even several seconds. Three primary strategies are employed to endow DDSs with photo-responsive behavior: (1) photo-induced intra/intermolecular reactions, including the formation of covalent bonds, bond cleavage, and isomerization, (2) activation of redox-responsive behavior due to photosensitizer-generated ROS via the photodynamic effect, and (3) activation of thermo-responsive behavior via the photothermal effect of some nanoparticles [651]. In many cases, the strategies enable an “on-off” behavior; the release can be started and stopped by applying and removing the photo-stimulation.

**Table 7:** Summary of photo-responsive mechanisms used in stimuli-responsive drug delivery systems.

Mechanism	Stimuli-Induced Response	Reference
Photo-induced cleavage	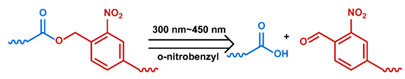	[159] ^c,^*** [169] ^c,^*** [170] ^c,^*** [171] ^c,^*** [268] ^b,d^ [269] ^c^ [246] ^b,c,d,^*** [650] ^c,^*** [652] ^c,^** [653] ^c,^***
	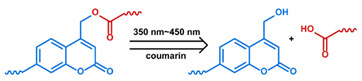	[172] ^c,^*** [654] ^a,b,c,^***
	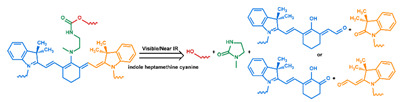	[244] ^a,b,c,^*** [655] ^b,c,^***
	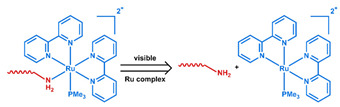	[656] ^b,c,^***
Photo-isomerization	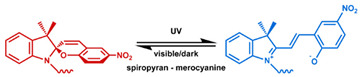	[178] ^d,^* [454] ^b,c,^** [455] ^b,c,^*** [657] ^a,b,c,^** [658] ^b,c^
	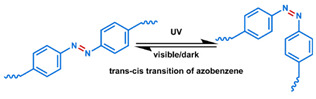	[221] ^c,^*** [230] ^b,c,^*** [659] ^c,^** [660] ^c,^* [661] ^d,^* [662] ^b,c,^*** [663] ^b,c^
Photo-induced bonding	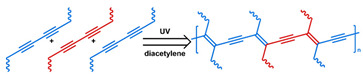	[118] ^c,^***
Photo-induced rearrangement	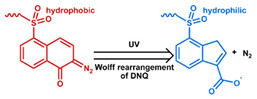	[48] ^b,c^
Photo-dynamic effect	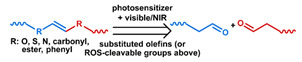	[167] ^b,c,^** [664] ^b,c,^* [665] ^b,d^ [666] ^a,b,c^ [667] ^b,c,^*** [668] ^c^ [669] ^c^ [670] ^c,^*** [671] ^b,c,^***
	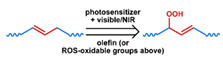	[485] ^a,b,c,^*** [672] ^a,c,^*** [673] ^a,c,^*** [674] ^a,b,c,^***
	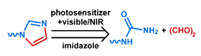	[675] ^a,b,c,^**
Photo-thermal effect	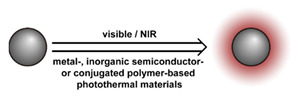	[21] ^b,c,^* [237] ^a,b,c,^** [252] ^a,b,c,^** [279] ^b,c,^** [456] ^a,b,c,^*** [467] ^c,^* [572] ^a,b,c,^** [578] ^a,b,c,^*** [588] ^b,c,^** [589] ^a,b,c,^*** [676] ^c,^*** [677] ^b,c,^*** [678] ^a,b,c,^** [679] ^a,b,c,^* [680] ^c,^*** [681] ^a,b,c,^***
Photo-acoustic effect	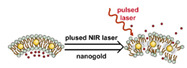	[682] ^c^ [683] ^b,c,^***

^a^ Evaluation included in vivo tests on mice.; ^b^ Evaluation included in vitro tests on cells.; ^c^ Small molecules, such as antitumor drugs and dyes, were used as payloads.; ^d^ Large molecules, such as proteins and plasmid DNA, were used as payloads.; * Premature payload leakage was >20%; ** Premature payload leakage was between 10% and 20%.; *** Premature payload leakage was ≤10%.

Several chemical groups have been demonstrated to undergo bond cleavage under appropriate photoirradiation. Examples include o-nitrobenzyl derivatives, o-alkylated aryl ketones, coumarin derivatives, and pyrene derivatives [684,685,686]. When these bonds are used in systems such as micelles and polymer nanoparticles, their cleavage destabilizes the system and induces the release of the payload. Photoirradiation can also induce bond formation, and this phenomenon has been exploited to disrupt lipid packing in membranes and trigger release from liposomes [118]. Finally, photo-induced isomerization reactions have been exploited to enable photo-induced drug delivery in several systems [687]. A classic example of a photo-induced isomerization is that observed for azobenzene derivatives. Under UV light (300–400 nm), the conformation changes from trans to cis, and the reverse occurs in dark conditions or when irradiated with visible light (>400 nm). This conformation change has been used as a valve in porous inorganic nanoparticles [663]. Azobenzene derivatives have also been utilized to form host–guest structures with macrocycles; conversion to the cis conformation weakens the interaction, releasing the complementary components in the host–guest pair [688]. Another well-known example is spiropyran, which is normally hydrophobic. When irradiated with 365 nm UV light, it converts to the more hydrophilic merocyanine [657]. Such a change has been exploited to induce release in micelles and nanogels [454,657].

The second strategy leverages ROS-induced release behavior. A photosensitizer capable of converting ground state oxygen to ROS upon irradiation is included in the formulation [689]. Optimization of these systems can be achieved by improving the performance of the photosensitizer and providing sufficient ground state oxygen. Zhang et al. reviewed recent advances and techniques for overcoming the drawbacks of conventional photosensitizers, such as poor biodegradability and weak photodynamic effect [690], and Liu et al. reviewed a new class of photosensitizers [691]. These materials not only overcome many drawbacks of conventional photosensitizers but also have a unique aggregation-induced emission (AIE) effect. It should be noted that endogenous factors such as the decreased O_2_ concentrations and increased GSH levels typically seen in cancer cells reduce the production of ROS via the photodynamic effect, limiting the effectiveness of this strategy for cancer therapy [692,693,694]. Suppression of GSH levels via metal adsorption [692], disulfide depletion [694], and GSH synthetase inhibition [695], and elevation of O_2_ levels via O_2_ transporting [696,697] and in situ conversion by intracellular H_2_O_2_ [693,698] have been demonstrated to overcome the hypoxia issue and enhance the photodynamic effect.

The third strategy relies on materials that can convert absorbed light into heat efficiently. Several materials exhibit a strong photothermal conversion efficiency, including carbon-based materials such as carbon nanotubes and graphene, metallic nanostructures such as gold nanorods, semiconductors such as MoS_2_, conductive polymers such as poly(pyrrole) and poly(aniline), and dopamine-melanin derived materials such as polydopamine [608]. By adding these materials, typically as discrete nanoparticles, many of the thermo-responsive systems discussed above can be endowed with photo-responsive behavior. Additionally, when pulsed irradiation is applied to photothermal agents, the photoacoustic effect can induce transient cavitation in an aqueous medium, similar to the effects of ultrasound. This transient cavitation can be used to disrupt liposome-based DDSs, releasing the contents [699].

#### 3.6.1. Photo-Responsive Micelles

As one of the most extensively studied systems, all three photo-responsive strategies have been applied to micelle-based DDSs. Photocleavable linkages and groups that undergo photo-isomerization can be placed in the hydrophobic backbone or used to link pendant groups to the amphiphile. Cleavage of bonds and the polarity change that accompanies isomerization disrupts the micelle releasing the payload. These techniques can be applied with others to create dual responsive systems such as in the work of Han et al., who explored a system that included both photocleavable o-nitrobenzyl methyl ester groups and redox-responsive disulfide bonds alternately spaced on the hydrophobic block backbone [650]. The redox response was slow, with ~50% of the payload being released over 4 h, while the photoresponse was fast, with ~15% of the payload being released in one minute. In contrast, Liu et al. used the photo-induced isomerization of 2-diazo-1,2-naphthoquinone (DNQ) to effect release [48]. Upon irradiation at 808 nm, Wolff-rearrangement converts the hydrophobic DNQ into the hydrophilic 3-indene carboxylic acid (3-IC), disrupting the micelle and releasing the payload. Hydrophobic spiropyran converts to hydrophilic merocyanine when irradiated at 250–380 nm, and Tong et al. demonstrated a system that used spiropyran isomerization when irradiated at 365 nm to release PTX [657,658]. However, it is challenging to apply irradiation in this wavelength range remotely without tissue damage. Irradiation in the NIR region is preferred for remote stimulation, as body tissue is more transparent to NIR wavelengths than higher energy photons. To enable the use of NIR for a spiropyran-based system, Chen et al. leveraged the upconversion behavior of nanoparticles doped with lanthanide ions to convert spiropyran to merocyanine [455]. A large number of transition states in the 4F electron shells of the upconversion nanoparticles (UCNPs) enables them to adsorb two or more low energy photons and emit a single high energy photon. Chen et al.’s system included an encapsulated UCNP in the core and responded to 980 nm irradiation. The response was quick, with 40% of the payload being released in 30 min.

Photosensitizers can be used to endow redox-responsive micelle-based DDSs with photo-responsive behavior. These systems rely on the formation of ROS when the micelle is irradiated and the subsequent redox-based response of the system. ROS reactions can be used to shift the hydrophilic–hydrophobic balance of the amphiphile, such as in the work of Dai et al., who co-encapsulated the photosensitizer, zinc phthalocyanine (ZNPC), and DOX in poly(propylene sulfide) (PPS)-PEG-based micelles [674]. Under visible irradiation (660 nm, 1 W/cm^2^), the ROS generated were sufficient to oxidize the sulfide groups, destabilizing the micelle, and releasing the payload. ROS reactions can also be used to cleave the amphiphile backbone, such as in the work of Saravanakumar et al. [664]. They utilized a redox-cleavable copolymer with PCL and PEG blocks conjugated via a vinyl dithioether to fabricate a responsive micelle. Both DOX and the photosensitizer, chlorin e6 (Ce6), were co-encapsulated in the micelle. Irradiation at 660 nm (50 mW/cm^2^) induced ROS generation, vinyl dithioether cleavage, micelle dissociation, and DOX release. Significant cytotoxicity of the system in HeLa cell assays was observed. They attributed part of the enhanced cytotoxicity to the elevated ROS levels achieved by the incorporation of the photosensitizer. Li et al. demonstrated this effect by encapsulating only Ce6 in poly(aspartic acid)-PEG-based micelles [675]. When irradiated (660 nm, 100 mW/cm^2^), the ROS produced oxidized imidazole groups attached to the poly(aspartic acid) blocks, inducing dissociation of the micelle. They reported significant improvement of Ce6 distribution in tumors and enhanced tumor growth retardation.

Similarly, photothermal materials added to thermo-responsive DDSs endow them with photo-responsive behavior. Many of the photothermal materials respond to NIR wavelengths, making these systems suitable for use in relatively deep tissues without the need for upconversion of the applied irradiation. As most of the thermo-responsive mechanisms rely on reversible processes, melting and crystallization, for example, these systems can exhibit “on-off” release behavior. For example, Wu et al. prepared UCST copolymer-based micelles and co-encapsulated Fe_3_O_4_ and DOX in them [572]. These micelles rapidly release their payload when irradiated at 808 nm (2 W/cm^2^) and quickly cease release as the DDS cooled when irradiation was removed. The response was very rapid compared to most DDSs. Similar rapid release and “on-off” behavior were reported by Hui et al. for their ICG and DOX co-encapsulated system [279]. This core-shell hybrid DDS was composed of a UCST micelle coated with a red blood cell membrane. A burst release of 20% of loaded the DOX occurred within five minutes when they irradiated the DDS (808 nm, 0.8 W/cm^2^). Similarly to the enhanced cytotoxicity effect seen in redox enabled photo-responsive systems, thermo-enabled photo-responsive systems can exhibit enhanced overall efficacy via thermally induced cell death.

#### 3.6.2. Photo-Responsive Liposomes

All three photo-responsive approaches also apply to liposomes. O-nitrobenzyl groups have been evaluated as the linker between the hydrophobic tail [653] and the lipid head and as part of the alkyl tail chains [652]. Irradiation at 360–365 nm cleaves the bond, destabilizing liposomes based on these lipids. In most cases, the response is fast, Bayer et al. reported that ~80% of the payload was released over one hour of UV irradiation. Bond formation can also be used to destabilize liposomes that contain unsaturated lipids. Yavlovich et al. utilized this approach by incorporating alkyne-containing lipids in the liposome and found that most of the calcein payload in their system was released within 30 min [118]. They proposed that crosslinking of unsaturated lipids caused them to aggregate forming defects and pores in the bilayers, enhancing the permeability of the lipid membrane. In addition to bond cleavage and formation, the conformational change of azobenzene upon irradiation has also been used to drive release from liposomes [659,700]. However, the inclusion of azobenzene has the unwanted side effect of impacting the membrane integrity leading to premature release [701]. Liu et al. attempted to address this issue by conjugating the azobenzene groups to cholesterol, immobilizing them [659]. However, they reported that the overall change in release rate upon irradiation was not significant and visible irradiation did not stop the release. Thus, they effectively eliminated the photo-responsiveness from the system.

The configuration and polarity changes upon oxidation of unsaturated lipids have been used to impart photo-responsive behavior to liposomes via redox-responsive mechanisms. Rwei et al. incorporated the hydrophobic 1,4,8,11,15,18,22,25-octabutoxyphthalocyaninato-palladium(II) (PdPC(OBu)8) as a photosensitizer into a 1,2-dilinoleoyl-sn-glycero-3-phosphocholine (DLCP)-based liposome that contained the anesthetic agent tetrodotoxin (TTX) [672]. The ROS generated by the photosensitizer, when irradiated at 730 nm (50 mW/cm^2^), oxidized the unsaturated lipid and enhanced release; on-demand, local anesthesia was observed in rats after 15 min of irradiation. A photosensitizer can also be incorporated into the lipid bilayer as done by Luo et al. [673]. They found that rapid release of the payload occurred within minutes upon irradiation at 660 nm (310 mW/cm^2^) of a porphyrin-phospholipid (PoP)-based liposome.

Photothermal agents are a simple way to endow thermo-responsive liposomes with photo-responsive characteristics. “On-off” behavior has been demonstrated for liposomes containing gold nanospheres irradiated at 250 nm [676] and gold nanorods irradiated at 808 nm [578] via the reversible melting of the lipid. A photo-acoustic effect results from pulsed NIR irradiation of gold nanoparticles and has also been used to enhance the release from liposomes through mechanical disturbance of the structure [682]. The photo-acoustic effect arises from rapid local heating, which generates vapor microbubbles that collapse violently, similar to cavitation induced by US. Wu et al. found that this phenomenon was enhanced when the Au particle was tethered to the lipid membrane. They later applied this technique to release CF from lysolipid-containing liposomes in just a few seconds without increasing the temperature of the surroundings [683].

#### 3.6.3. Photo-Responsive Polymeric Nanoparticle

For polymeric nanoparticles, photo-degradability can be achieved by the insertion of photo-sensitive linkages in the polymer backbone or as a crosslinker. Almutairi’s group conducted a series of work in this field [169,170,171,702]. They found that under appropriate irradiation, an o-nitrobenzyl derivative could initiate a self-immolation process to degrade the polymer. This process was rapid under UV irradiation (350 nm), occurring in one minute, but significantly slower under NIR irradiation (750 nm), taking three hours. They attempted to address this issue by incorporating a UCNP and found that when this system was irradiated at 980 nm, the payload was released in one hour. In yet another design, they placed a “trigger” group at the terminal position enabling a domino mode of self-degradation. A burst effect was still observed under UV irradiation, but NIR irradiation (750 nm) induced release was faster, 2 h, in the absence of the UCNPs [169]. A comprehensive summary of self-immolative groups was presented by Wong et al. [700]. In nanogel-based systems, the photo-responsive group can be located in the crosslink in addition to the polymer backbone. Both the cleavage of covalent bonds and the weakening of noncovalent crosslinks have been reported. Coumarin-based crosslinkers were evaluated by Huang et al. [172]. They found that UV irradiation (365 nm) cleaved the crosslinker leading to swelling or dissociation of the nanogel and release of the payload. Peng et al. evaluated a noncovalent crosslinker based on a host–guest interaction between azobenzene-bearing dextran and β-CD-bearing dextran [661]. Irradiation of this system at 365 nm induced isomerization of the azobenzene, weakening the host–guest interaction and releasing the payload. The UV initiated spiropyran–merocyanine transition has also been used to promote release from nanogels [454]. Gu et al. utilized a photo-thermal response to enable the enzymatic degradation of a poly[acrylamide-co-N-(3-aminoprypyl)-methacrylamide]-based DDS [159]. Their system used peptide-based crosslinkers that were conjugated with an o-nitrobenzyl photo-sensitive group to prevent digestion by CP3 encapsulated in the DDS via noncovalent adsorption. UV irradiation (409 nm) was served to cleave the o-nitrobenzyl photo-sensitive group enabling the digestion of the crosslinker by CP3. Cleavage of the crosslinker released more CP3. This positive feedback mechanism accelerated the release of the apoptosis-inducing agent. The limited penetration of UV and premature release of the nanogel remain as obstacles to the application of these systems.

Redox-responsive bonds have also been leveraged to enable photo-responsive properties in polymer nanoparticle-based DDSs. For example, Yuan et al. synthesized an amphiphilic copolymer containing AIE photosensitizer, ROS-cleavable amino acrylate (AA) links, and oligoethylenimine (OEI) [665]. Under visible irradiation (640 nm), the ROS generated from the photosensitizer cleaved the AA bonds, degrading the polymer and releasing the payload, pDNA. There are few reported attempts to combine photosensitizers and ROS-sensitive groups to achieve photo-responsive nanogels. One exception is the work of Xu et al., which demonstrated a system based on the photosensitizer Zn(II) phthalocyanine and ROS-cleavable thioketal groups [666]. When irradiated at 600 nm (100 W) for 5 min, sufficient ROS was generated to cleave the thioketal links and release the DOX payload.

Photothermal agents have also been incorporated into nanogels to confer photo-responsiveness. Similar to liposomes, gold nanoparticles are commonly used for this application. One enhancement to these materials is to coat them with silica, which enhances their stability enabling higher irradiation intensities. For example, Zhange et al. incorporated a silica-coated nanogold in a PNIPAM-based nanogel [679]. Under NIR (760 nm, 16 W/cm^2^), temperature elevation, shrinkage of the nanogel, and polarity change of the polymer chain promoted the release of the payload, DOX, and enhanced extravasation from the blood vessels in the tumor. However, while liver dysfunction was not observed, the significant liver and spleen accumulation of the DDS and the DOX leakage reported merits further evaluation. A unique approach to photo-thermally responsive polymer nanoparticles was demonstrated by Viger et al. [677]. The mechanism they developed utilizes the NIR (980 nm)-induced resonance of water in confined nanodomains. These domains resulted from the hydration of PLGA chains located on the particle surface. During irradiation, the water nanodomains were directly heated, increasing the temperature above the T_g_ of the polymer and promoting the release of the payload. The response was rapid—80% of the loaded NB was released in 5 min—and the system exhibited an “on-off” response. The advantage of this system is that it avoids the need for additional nanoparticles to mediate the photothermal process, using only FDA-approved polymer as a substrate.

#### 3.6.4. Photo-Responsive Porous Inorganic Nanoparticle

Photo-responsive gatekeepers have been extensively studied, and enabling mechanisms include photo-cleavable covalent bonds, photo-isomerization to control noncovalent binding, ROS-cleavable bonds, and thermo-labile bonds. He et al. demonstrated the use of UCNPs encapsulated in an MSN core to allow cleavage of a linkage between a ruthenium complex and a primary amine under NIR (980 nm) irradiation [656]. Depth of penetration was demonstrated by conducting an in vitro test by applying the NIR through a 1 mm thick pork tissue. Photo-isomerization mediated noncovalent binding based on host–guest interactions between β-CD and azobenzene groups has also been utilized [221]. UV irradiation (365 nm) initiates trans-to-cis isomerization of the azobenzene, weakening the interaction and releasing the gatekeeper and payload. Azobenzene has also been used as the gatekeeper for MSN [662]. In the trans conformation, the pores were blocked, while UV-initiated conversion to the cis conformation opened the pores, releasing the payload. UCNPs were incorporated in the system to enable the response from NIR (980 nm) irradiation. Relaxation to the trans configuration in the absence of UV or NIR irradiation resulted in an “on-off” capability for the system. Azobenzene derivatives have also been used as the organic ligands in MOF structures [703,704]. In Epley et al.’s study, MOF was fabricated using Zr and azobenzenedicarboxylate (AZB) [230]. The trans-to-cis transition of AZB under 1000 W white light induced degradation of the MOF structure and the payload was gradually released over several hours. Preliminary in vitro testing demonstrated the successful delivery of NR into HeLa cells. However, though the MOF was mostly degraded in 8 h, the release was prolonged, and there was no apparent photo-induced release of the second payload, IBU, which is highly hydrophobic. As with other systems, photo-initiated cleavage of a ROS-cleavable bond requires the addition of a photosensitizer. For example, Wang et al. utilized the photosensitizer methylene blue to initiate the cleavage of a vinyl dithioether linker used to attach a β-CD-adamantane supramolecular gatekeeper [667]. Finally, the photothermal effect can be used to remove gatekeepers tethered by host–guest interactions or thermo-labile azo bonds. Li et al. used temperature elevation due to NIR (808 nm, 17.6 W/cm^2^) to weaken the host–guest interaction between a sulfonatocalix[4]arene cap and a quaternary ammonium salt used as a gatekeeper [680]. Lei et al. utilized the photothermal response of ICG to cleave the azo bond tethering β-CD gatekeepers in their MSN-based DDS [331].

#### 3.6.5. Photo-Responsive Non-Encapsulated DDDs

As for drug conjugates, controlled release can be achieved using photo-cleavable groups or ROS-sensitive linkages. Griffin et al. synthesized a series of o-nitrobenzyl-containing monomers with different reactive groups at the benzylic position. They demonstrated that nearly all therapeutic agents could be conjugated with at least one of these monomers [268]. In a further study, they modified the o-nitrobenzyl groups so that they could be cleaved via irradiation at different wavelengths [269]. This enables the formulation of complex hydrogel systems in which multiple drugs can be loaded independently and each released by irradiation at a unique wavelength. Similarly, Azagarsamy et al. utilized two photocleavable linkers, o-nitrobenzyl and coumarin, to independently control the release of different therapeutic agents (365 nm for coumarin, 405 nm for o-nitrobenzyl) [246]. In another work, Schnermann’s team used a cyanine-based photo-responsive prodrug capable of releasing conjugated payload under NIR (690 nm) [655]. A photooxidation-intramolecular cyclization mechanism was proposed for the process. Drug conjugates can be combined with other DDSs to protect the payload. Zhao et al. prepared a UCNP-MSN core-shell structure and loaded it with chlorambucil-conjugated coumarin-based prodrug via ethanol [654]. Upconversion of NIR irradiation (980 nm, 570 mW/cm^2^) by the UCNPs resulted in cleavage of the ester bond linking the chlorambucil payload, resulting in its release. The system also exhibited an efficient “on-off” release behavior. Similarly to other systems that use photoirradiation initiated ROS cleavage of linkers, photosensitizers are required when using ROS-cleavable linkers in photo-responsive drug conjugates. Under irradiation, the ROS generated by the photosensitizer induces cleavage of the linker and release of the payload, as well as synergistically enhancing the antitumor efficacy of the system [668,669,670]. A unique application of this technique is the two-step activated prodrug developed by Hossion et al. [671]. The photosensitizer, 5-(6)-carboxyfluorescein, was deactivated before administration and reactivated in vivo in the presence of intracellular esterase. Under visible light (540 nm, 8 mW/cm^2^), the ROS generated from the photosensitizer cleaved the amino acrylate linker releasing the payload. The requirement of two stimuli for activation of release is called “logic” based release and is one promising method to enhance efficiency and reduce off-target effects.

In contrast to bond cleavage, photothermal heating is a facile mechanism to enhance the release of drug loaded on nanoparticles via adsorption. The local heating weakens the noncovalent interaction and enhances drug diffusion from the DDS. NIR irradiation results in significant temperature elevation and rapid drug release from these systems [237,467,681]. For example, in Yin et al.’s work, a chitosan-complexed MoS_2_ nanosheet was used to deliver DOX [678]. The significant temperature elevation due to NIR (808 nm, 1.4 W/cm^2^) dramatically accelerated DOX release and led to a quasi “on-off” mode release.

#### 3.6.6. Photo-Responsive DDSs: Limitations and Remaining Issues

Although photo-induced drug delivery is precise, convenient, and rapid, it has several drawbacks. First, UV or visible light is typically required for responses based on bond cleavage or ROS generation. Unfortunately, UV light can damage nucleic acids, leading to safety concerns [705,706], and tissue penetration for both UV and visible light is limited. Techniques to apply NIR, which has fewer side effects and better tissue penetration, are being developed. Compounds that respond via a two-photon adsorption effect, possess a lower energy barrier, or exhibit aggregation-induced emission are promising [707,708,709,710,711]. Irradiation and energy conversion techniques such as second harmonic generation (SHG), enhancement of two-photon absorption (TPA), UCNP, triplet-triplet annihilation upconversion (TTAUC), and generation of light energy from other forms of energy are also potential solutions to this issue [712,713]. Second, materials that exhibit these energy conversion behaviors, as well as compounds that can initiate a photo-induced chemical reaction and photosensitizers, usually possess complex chemical composition; thus, the biocompatibility, biodegradability, and long-term safety of them and their degradation products require careful evaluation [685]. Biocompatibility and biodegradability are also concerns relating to some photothermal materials [714,715]. The safety and cost of advanced light sources also require further evaluation and development. Third, while NIR exhibits deep tissue penetration, up to 10 cm below the skin [664], biological constituents in the path have substantial absorption of NIR [716]. For some DDSs, several minutes of intense irradiation is required, raising concerns regarding safety [651].

### 3.7. Ultrasound-Responsive DDSs

Another exogenous stimulus that has found application in responsive DDSs is ultrasound, Table 8. It has been used for biomedical imaging for many years. High-intensity focused ultrasound (HIFU) can be focused precisely on a local region of the body and has been demonstrated to cause little to no damage as the longitudinal pressure wave passes through the dermis and body tissue. In general, higher frequencies enable the energy to be focused more precisely and on a smaller volume [717] but result in enhanced attenuation and reduced penetration depth [718]. Generally, the biomedical ultrasound frequencies are in the 0.5–50 MHz range; HIFU uses the lower end of this range, 0.8–3.5 MHz [717,719]. Focused ultrasound can induce cavitation, acoustic streaming, and local hyperthermia in the focal volume. It can also induce bond breakage via the strain induced by the alternating pressure field. Ultrasound-responsive DDSs utilize these responses to activate drug release.

**Table 8:** Summary of ultrasonically induced response mechanisms used in stimuli-responsive drug delivery systems.

Mechanism	Stimuli-Induced Response	Reference
Cavitation	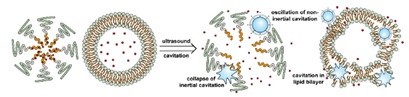	[57] ^c^ [58] ^a,b,c,^*** [107] ^c^ [108] ^c,^** [720] ^b,c^ [721] ^a,c^ [722] ^c^ [723] ^c^ [724] ^c,^*** [725] ^b,c,^**
	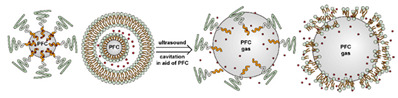	[55] ^a,b,c^ [113] ^b,c,^*** [114] ^b,d^ [500] ^b,c,^** [726] ^b,c^
	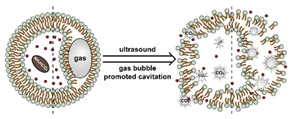	[109] ^d,^* [458] ^b,c,^** [727] ^c,^** [728] ^b,c,^*
Thermal effect	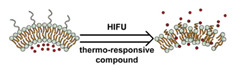	[575] ^a,b,c,^** [729] ^c,^*** [730] ^a,b,c,^*** [731] ^c,^*
Acoustic streaming	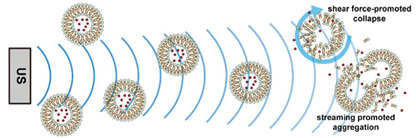	[732] ^c^
Bond cleavage	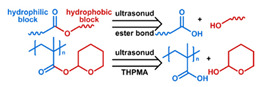	[46] ^c,^*** [59] ^c^ [61] ^c^ [60] ^c^ [214] ^b,c,^** [733] ^c,^***
	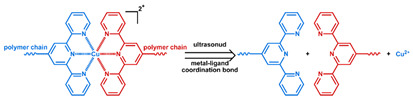	[734] ^c,^***

^a^ Evaluation included in vivo tests on mice.; ^b^ Evaluation included in vitro tests on cells.; ^c^ Small molecules, such as antitumor drugs and dyes, were used as payloads.; ^d^ Large molecules, such as proteins and plasmid DNA, were used as payloads.; * Premature payload leakage was >20%; ** Premature payload leakage was between 10% and 20%; *** Premature payload leakage was ≤10%.

As the pressure waves propagate through the tissue, the medium experiences compression and rarefaction. Cavitation results from the evaporation of the medium during rarefaction, and collapse of the resulting microbubbles during compression. This phenomenon is enhanced as the medium is heated due to ultrasound-induced hyperthermia. Cavitation can be classified as non-inertial and inertial (or transient) cavitation. Non-inertial cavitation is characterized by stable microbubbles that experience volumetric oscillation in moderate-intensity ultrasound. In contrast, the microbubbles that characterize inertial cavitation go through nucleation and violent collapse. A parameter correlated to the cavitation is the mechanical index (MI), which is defined as the local peak negative pressure (PnP, unit MPa) divided by square root of the frequency (F_c_, unit MHz), MI = PnP⋅F_c_^−0.5^ [735]. In the range of biomedical ultrasound, higher MI results in more inertial cavitation, which can maximize the disturbance to nanocarriers and induce drug release [736]. Relatively low frequency and moderate amplitude ultrasound can achieve comparable or even superior drug release efficiency compared to high-frequency large-amplitude ultrasound. Thus, low frequency is favored in triggering release [57,113,737]. However, at least one study reports a synergistic effect from combining low and high-frequency ultrasound [721]. Free radicals are generated during cavitation under ultrasound, suggesting that this behavior might be used to trigger release from redox-responsive DDSs [738]. However, to the best of the authors’ knowledge, no ultrasound-triggered redox-responsive DDSs have been reported in the literature. In addition to cavitation in the liquid adjacent to the nanocarriers, cavitation can also occur in the hydrophobic regions of DDSs such as the lipid bilayer of liposomes, potentially initiating release from these systems via mechanical disruption of the bilayer [739,740]. Larger molecules can experience stretching forces in an ultrasound field, potentially leading to the cleavage of stressed bonds [741]. Moore’s group performed an extensive study of polymers that can be cleaved via mechanical stretching [742,743,744]. Xia et al. conducted a series of studies examining the use of this effect in ultrasound-responsive DDSs (see Section 3.7.1 below).

Bulk acoustic streaming is the motion of particles in the direction of the wave propagation that results from the reflection and scattering of the ultrasound wave in the medium. Cavitation can also induce microstreaming [745,746]. Bulk streaming can promote the motion of the DDS and released drugs in a particular direction, deeper into the tumor, for example. It can also improve the probability of nanoparticle collisions. This effect is potentially important for liposome-based DDSs in which collisions induce membrane fusion, destabilizing the bilayer, and promoting payload release [732]. Microstreaming can also induce high shear stress, destabilizing nanocarriers.

The vibration and collision of fluid molecules, as well as the friction between the fluid and surrounding tissue, convert the US wave energy into heat. When HIFU is applied, local hyperthermia can be induced remotely, enabling thermo-responsive DDSs to be triggered by ultrasound [747]. Recent advances in MRI techniques enable HIFU to be guided via MRI, increasing the precision and safety of therapeutic hyperthermia via ultrasound [748,749].

In addition to triggering drug delivery, ultrasound can also disturb vascular endothelial integrity and the cellular membrane and, thus, can be used to increase extravasation at the tumor site and cellular uptake. This effect is related to microbubbles, and several mechanisms have been proposed [750,751]. It is also reported that the combination of ultrasound and microbubbles can open the blood–brain barrier (BBB), allowing the delivery of 10 nm nanoparticles to the brain via the circulatory system [751]. While still smaller than many DDSs, this is the size range of drug conjugates and dendrimers [752].

#### 3.7.1. Ultrasound-Responsive Micelles

Typically, ultrasound-induced drug release from micelles is activated via disruption of the micelle by cavitation. Mohan et al. used 3 MHz ultrasound to induce the release of DOX from PEG-PCL micelles [726]. Pruitt et al. demonstrated that ultrasound irradiation increased the speed of action of DOX-loaded Pluronic micelles, 50% viability of human leukemia (HL-60) cells was achieved at 20 h in the absence of ultrasound but after only ~2 h with ultrasound [720]. This effect was demonstrated in vivo by Rapoport et al. for PTX loaded PLA-PEG micelles with 1 MHz ultrasound [55]. As the primary driver for micelle disruption in these systems is cavitation, these studies found that low-frequency ultrasound was preferred. In contrast, Hasanzadeh et al. reported that a combination of low and high-frequency ultrasound resulted in the highest release rates of DOX from Pluronic-based micelles [721]. Intriguingly, several researchers have reported the ability of ultrasound-responsive micelles to re-encapsulate the payload when the ultrasound stimulus is removed [57,58,722]. Wu et al. observed this quasi “on-off” behavior in curcumin-loaded Pluronic micelles stimulated with 1.9 MHz ultrasound [58]. Shen et al. noted that in PNIPAM-PEI micelles with co-encapsulated DOX and perfluorohexane (PFH), ultrasound resulted in vaporization of the PFH and 100% of the DOX being released [500]. However, after the ultrasound was removed, DLS indicated that the micelles reformed, albeit with a smaller size due to the removal of the PFH from the system. Whether or not such behavior occurs to a significant extent in vivo remains a question, as it is difficult to inspect nanocarriers after administration and cellular processes affect drug transport and micellar reformation.

Disruption of the micelle can also be caused by the ultrasound-initiated cleavage of covalent bonds. Most efforts to date have focused on ester bonds [59,60,61]. Xia et al. attached 2-tetrahydropyranyl methacrylate (THPMA) as a hydrophobic pendant group to block copolymers and fabricated the resulting materials into micelles. Application of 1.1 MHz HIFU cleaved the ester bond connecting the pendant group changing the hydrophilic–hydrophobic balance of the system releasing the NR payload [60,61]. They later demonstrated that other ester bonds are ultrasound labile such as the ester bond linking PLA and PEG in a deblock copolymer [59,753]. MW determination after ultrasound-induced degradation indicated that the cleavage occurred at the link between the two blocks, and they suggested that the low bond energy (~268 kJ/mol) and longer bond length (~2.03 Å) would enable the ultrasound-induced cleavage of disulfide bonds as well. This behavior would have provided a route to combined ultrasound- and redox-responsive micelles. However, the FTIR spectrum of degradation products revealed that cleavage occurred at the ester bond, not the disulfide bond [46]. It has also been suggested that triazole groups were cleavable via ultrasound [650]. However, Xia’s team concluded it was the hydrolysis of adjacent ester bonds that was responsible for the cleavage of the polymer chains [733]. They also demonstrated that coordination bonds, Cu(II)-terpyridine (Tpy)-connected PEG and PPG blocks, for example, could be cleaved via HIFU [734].

#### 3.7.2. Ultrasound-Responsive Liposomes

Cavitation nucleated in the aqueous core of the lipid bilayer has been used to initiate the release of therapeutic agents from liposomes. These nucleated cavitation sites grow until they form a pore through the bilayer releasing the payloads [739,740,754]. Lin et al. demonstrated this phenomenon by releasing calcein from liposomes upon application of 20 kHz ultrasound [108]. They found that at gel–fluid transition temperatures, the effect was enhanced. However, the low fraction of calcein released (~20%) suggests that this effect is not extensive in ordinary liposomes. Three strategies are employed to enhance the ultrasound responsiveness (sonosensitivity) of liposome systems. First, heterogeneous components are incorporated in the lipid bilayers to impair the membrane integrity. For example, by varying the composition of 1,2-distearoyl-sn-glycero-3-phosphoethanolamine (DSPE) and cholesterol, Evjen et al. successfully enhanced the sonosensitivity of a DSPE-based liposome [723]. They demonstrated a 7-fold increase in release rate over the standard PEGylated liposome. They extended this idea by incorporating saturated DSPC into unsaturated DOPE-based liposomes, finding that the effect was diminished at DOPE content of 55–65% [107]. Similar effects were demonstrated by Mujoo upon the addition of bile salts to soy lecithin liposomes; all bile salts increased the sonosensitivity of the system, but taurocholate had the greatest effect [724]. However, they reported that the addition of taurocholate to DOPE-based liposomes decreased sonosensitivity and hypothesized that bile salts could undermine the integrity of the soy lecithin-based membrane, but formed a more stable structure in DOPE-based bilayers.

The second technique used to enhance the sonosensitivity of liposomes is to entrap gas in the liposome core or lipid bilayers. Huang et al. conducted extensive studies of liposomes in which air was incorporated in the bilayer via mechanical agitation [109,729]. They found that the gas not only deteriorates membrane integrity but also amplifies cavitation when the liposomes are exposed to ultrasound. A 2–3-fold improvement in the ultrasound-responsive release was observed. They also reported that when nitric oxide was co-encapsulated in the liposome core, it not only enhanced sonosensitivity but also enhanced contrast in ultrasound imaging was observed [728]. One issue that Huang et al. noted was that the entrained gas leaked rapidly from the prepared liposomes. To address this issue, Nahire et al. incorporated NH_4_HCO_3_ into liposomes, so that gas would be generated under acidic conditions [458]. The system required two stimuli, a pH drop to 5 to stimulate CO_2_ production and ultrasound at 1 MHz to enhance CO_2_ production and liposome destabilization.

The third approach to enhance sonosensitivity is to incorporate perfluorocarbon (PFCs) droplet into the liposome core. Lin et al. demonstrated the emulsification of PFC droplets by single layer lipid membranes and the co-incorporation of the droplets with a payload into liposomes via agitation [113]. The enhanced the sonosensitivity of the system was demonstrated by comparing in vitro cell assay results from PFC loaded and PFC free liposomes for the delivery of DOX to HeLa cells. Javadi et al. reported a similar effect for a calcein delivery to HeLa cells but observed a weaker effect for green fluorescent protein (GFP) pDNA payloads [114]. They also reported that there was not an obvious relationship between ultrasound power and cell uptake, suggesting that more work is required in this area.

Hyperthermia can be used to endow thermo-responsive liposomes with ultrasound-responsive behavior. Three primary approaches have been explored; the gel–fluid transition of conventional lipids, pore formation by lysolipids, and destabilization of the lipid bilayer via the insertion of thermosensitive polymers [575,729,730]. Novell et al. incorporated cholesterol to reduce the gel-fluid transition temperature in a DPPC/DSPE-PEG liposome [729]. While the system was very leaky, i.e., 25% of the loaded calcein was released in PBS buffer after 5 min, the system was sensitive to ultrasound. After 5 min of HIFU exposure, over 50% of the loaded calcein was released. Liang et al. explored the use of the lysolipid, MSPC, to enhance sonosensitivity [730]. They also enhanced bilayer stability through the addition of triethoxysilane containing lipid to form a silica shell on the outer surface of the membrane. Calcein, DOX, and NR were evaluated as model drugs. In the absence of HIFU, only NR leaked from the system, <2.5%. When HIFU was applied, all the payloads were released rapidly. During in vivo studies, tumor size reduced from 100 to 25 mm^3^ in MDA-MB-231 tumor-bearing mice after 16 days of administration. However, the highest accumulation occurred in the liver. Park et al. explored the insertion of a thermo-responsive peptide as an approach for ultrasound-responsiveness in DPPC-based liposomes [575]. The peptide was connected to a lipophilic tail and possessed a tunable LCST between 37 and 50 ℃. During the in vivo test in SCC-7 tumor-bearing mice, the DOX-loaded liposome resulted in obvious tumor growth retardation with HIFU (1 MHz) exposure. However, the system exhibited significant drug leakage, which may partially be the reason that the therapeutic efficacy was not as good as that reported in Liang et al.’s study [730].

There are few studies of acoustic streaming as a mechanism for ultrasound-responsive liposomes. For example, Oerlemanse et al. evaluated the effects of ultrasound on release in a thermostatic water bath for both thermo-responsive and non-thermo-responsive liposomes [732]. When HIFU was applied, both systems exhibited substantial release of the payloads over 30 min. As there was no temperature increase, they attributed the release to acoustic streaming, which caused the liposomes to collide with each other and the walls of the container. The high shear forces resulted in liposome destabilization and payload release. It is unclear how this phenomenon can be leveraged in a DDS. Thus, while intriguing, evaluations of acoustic streaming as a release mechanism in biological systems is yet to be conducted.

#### 3.7.3. Other Ultrasound-Responsive Systems

Nearly all studies of ultrasound-induced drug release utilize micelle or liposome DDSs. Notable exceptions are the work of Dermirel et al. [731]. on ultrasound-responsive nanogels and the reports of Paris et al. [214] and Li et al. [725]. on polymer-coated MSN DDSs. Dermirel et al. demonstrated that ultrasound accelerated the release rate of RhB from a poly(vinyl caprolactam-co-2-dimethylaminoethyl methacrylate)-based nanogel [731]. They attributed this behavior to the vigorous mechanical disturbance from the ultrasound enhancing diffusion rates. Paris et al. incorporated the ultrasound sensitive monomer THPMA into a thermosensitive copolymer immobilized on an MSN to prepare dual responsive MSN DDSs. At normal physiological temperatures, the copolymer was hydrophobic and collapsed into a globular state serving as a gatekeeper. However, the application of ultrasound cleaved the ester bond on THPMA, reducing the hydrophobicity of the copolymer and causing it to adopt an extended conformation releasing the payloads. Li et al. explored PDA as an ultrasound-responsive coating on MSN DDSs [725]. Under the mechanical agitation from 1.1 MHz HIFU, the permeability of the PDA layer was increased, releasing the DOX payload. The layer recovered upon the removal of the HIFU, enabling an “on-off” release behavior. While leakage was observed for the system, it was significantly lower than for the nanogel in Dermirel et al.’s study and even some silica-coated liposome systems [575,731].

#### 3.7.4. Ultrasound-Responsive DDSs: Limitations and Remaining Issues

There have been few studies of ultrasound-responsive nanocarriers beyond micelles and liposomes. Considering the abundant thermo-responsive techniques applied to other systems, in the authors opinion, ultrasound-induced drug delivery systems based on ultrasound-induced hyperthermia merit more exploration. Ultrasound-induced hyperthermia is particularly relevant for tumors seated in deep tissues, as HIFU enables ultrasound to reach relatively deeper locations facilely compared to AMF and photoirradiation. However, some problems remain to be solved to enable ultrasound DDSs to find clinical applications. The main issue is the relatively low ultrasound response of small nanocarriers. Nanocarriers should be relatively small, usually less than 250 nm, to take advantage of the EPR effect and avoid rapid clearance during circulation [7]. This compact structure and small dimensions are not favorable for achieving high sonosensitivity. Though some sonosensitive materials can be incorporated in nanocarriers to enhance their response to ultrasound, they impact DDS stability and reduce circulation half-life in physiological environments [723]. While microbubbles can be used as nucleation sites to enhance cavitation and sonosensitivity of micron-scale nanoparticles, these large particles typically have poor circulation half-life and exhibit unfavorable biodistribution [7,755]. Recent advances include the use of phase-change agents such as PFCs and gas generating agents such as NH_4_HCO_3_, which have small volume until vaporized by ultrasound or reacted at low pH [756,757,758,759]. However, the difficulties in exclusion and degradation of PFCs raise safety concerns [760,761], and NH_4_HCO_3_ may also respond to inflammation tissues with low pH [482,483]. Another potential improvement in ultrasound-responsive DDSs might be enabled by the report of Kwan et al. on a nano-cup capable of trapping gas within the cavity [762]. They demonstrated that during the US rarefaction cycle, the gas could be released. Extended circulation half-life and good ultrasound responsiveness make this nano-cup another promising agent to enhance ultrasound responsiveness. In addition to the sonosensitivity problem, ultrasound cannot penetrate air-filled regions. Thus, the strategy is not appropriate for tumors beside or surrounded by organs such as the lungs and bowels [717]. Even if the tumor site is accessible for ultrasound, to avoid physical damage, the MI for biomedical ultrasound application is limited to 1.9, which is why high sonosensitivity is required for ultrasound-responsive DDSs [763]. Finally, while the HIFU technique penetrates deep into the tissue, MRI guidance is required to optimize therapeutic efficiency. Combined HIFU-MRI systems are complex and expensive.

### 3.8. Stimuli-Induced Targeting

As noted, a PEG coating can enhance the circulation life of DDSs; however, the hydrophilicity and steric hindrance reduce cellular uptake. Targeting ligands can enhance cellular internalization but increases clearance via the MPS system. Additionally, some target ligands exhibit off-target effects [764]. While the 30–200 nm dimension is suitable for passive targeting via the EPR effect, crossing the physiological barriers and achieving deep tumor penetration requires diameters on the order of 20 nm or less [765,766,767,768]. In this range, the extravasated nanocarriers can return to blood vessels and be excreted by the renal system [7,315]. Furthermore, the neutral/weakly negative and hydrophilic surface helps limit clearance [7], while hydrophobic and positively charged surfaces promote cell adhesion and uptake [769,770], or, in the case of liposomes, fusion with the cellular membrane [130,132]. Thus, in general, the properties needed to reduce the clearance rate via the MPS system limit deep tumor penetration and cellular uptake. A potential solution is the development of DDSs with stimuli-responsive targeting behavior (Table 9). Three strategies have been reported in the literature for stimuli-responsive targeting: (1) stimuli-induced target exposure, (2) stimuli-induced size change, and (3) stimuli-induced surface property change. As these changes are engineered to occur in tumor ECM, the specificity requirement of target ligands can also be relaxed. To enable the response pH cleavable groups such as maleic acidic amide derivatives (hydrolyzed at pH 6.5–6.8) [771], compounds that undergo protonation such as chemicals with pKa/pKb close to 6.8 (such as poly(L-histidine) [772], poly(β -amino ester) [773], sulfonamide [774], and substrates of enzymes typically overexpressed in tumor ECM are used.

**Table 9:** Summary of stimuli-responsive targeting strategies.

Strategy	Stimuli-Induced Response	References
Exposure of target ligands	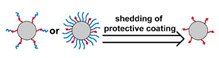	[180,208,331,774,775]
	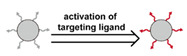	[776,777,778,779]
Change in size of nanocarriers	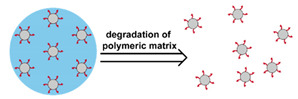	[158,441,780,781,782,783,784]
	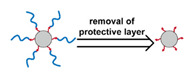	[774,785,786]
	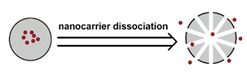	[772,773,787,788]
Change in surface property of nanocarriers	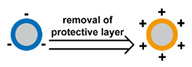	[771,784,789,790]

#### 3.8.1. Stimuli-Induced Targeting Ligand Recovery

Recovering the activity of shielded or deactivated targeting ligands or cell-penetrating peptides (CPP) in the ECM can be used to enhance endocytosis while limiting their effects on clearance or “off-targeting” behavior. This approach has been used in both MSN and micelle-based DDSs [180,208,331,775]. Zhang et al. linked an RGD targeting ligand to an MSN-based DDS and decorated the ligand with poly(aspartic acid) (PASP) via an MMP cleavable Pro-Leu-Gly-Val-Arg (PLGVR) peptide [208]. The shielded RGD was not able to regulate cellular uptake. However, as the MSN extravasated into tumor ECM, MMPs removed the PASP layer by digesting the GRVGLPH peptide, exposing the RGD ligand and enhancing endocytosis. Yoo et al. extended this approach to micelle-based DDSs by synthesizing an amphiphile that included a PEG shielded poly(L-lysine)-based CPP [775]. The PEG end was removed via digestion of the PLGLAG linker by MMP in the ECM, enabling the poly(L-lysine) to promote cellular uptake. 

Deactivated CPPs have also been explored in prodrug systems. Jiang et al. prepared a fluorophore-poly(arginine) conjugate and deactivated the poly(arginine) CPP by complexing it with an anionic peptide, poly(glutamic acid), via an MMP-2 cleavable linker [778]. The cellular uptake of the fluorophore by fibrosarcoma (HT-1080) cells was roughly three times the uptake of a similar system in which a scrambled linker replaced the MMP-2 cleavable linker. Bode et al. reported an elegant proof-of-concept study, in which alanine (ALA) and glycine-proline dipeptide (GP) were conjugated to the lysine residues of a transactivating transcriptional activator (TAT) [779]. The CPP capacity of the modified TAT was deactivated by the modification of as few as two lysine residues. In the presence of corresponding enzymes, the CPP capacity of TAT was recovered. Similarly, CPP deactivation via legumain-removable pendants was used in Liu et al.’s study of stimuli-responsive targeting in liposomes [777]. Useful for targeting stimuli in the tumor ECM are not limited to enzymes. Jin et al. fabricated a smart micelle with TAT deactivated via acid-labile amide [776]. Using the hydrolysis in weakly acid tumor ECM, the CPP functionality of the TAT was recovered. The clearance of the micelle system in mice was significantly reduced. However, as the pH reduction in the tumor ECM was limited, enhancement of the tumor inhibitory effect and tumor accumulation were not as noticeable as enzyme-activated systems.

#### 3.8.2. Stimuli-Induced Size Change

Decreasing the size of nanocarriers after they extravasate into the tumor enables deeper penetration. Three approaches have been explored to achieve size reduction of DDSs in the tumor ECM—the breakup of DDSs that are composed of smaller particles, the degradation of polymer coatings, and the dissociation of aggregated small DDS particles. Wong et al. confirmed that the first approach merited further exploration [158]. They encapsulated 10 nm quantum dots (QD) in 100 nm gelatin nanoparticles. Deeper (up to 300 mm from the injection site) and more homogeneous tumor distribution of the QDs was observed compared with 100 nm silica particles. Gao’s group studied the degradation of gelatin-based DDSs to release smaller DDS particles [780,781,782,783]. In the ECM, MMPs degraded the nanogel, releasing the dendritic DOX-conjugates enabling deeper penetration into the tumor [780]. In later studies, they encapsulated DOX-loaded iRGD-decorated gold nanoparticles in gelatin nanogel and co-administered the adjuvant agent Losartan. Losartan reduces the tumor ECM density enabling deeper penetration into the tumor mass [781,782]. More recently, Gao’s group demonstrated similar behavior for dendritic DOX-conjugates encapsulated in HA-based nanogels [783]. The degradation of the HA in the presence of hyaluronidases released the dendrimer. In this system, they also incorporated a photosensitizer and nitrooxy groups to enable NIR-stimulated release of nitrous oxide, to enhance ECM permeability. A similar approach can be used with micellar-based DDSs. In Li et al.’s work, a micelle DDS was used to deliver dendritic prodrug to tumors [441]. The prodrug was attached to PCL blocks via dimethylmaleic amide. At pH 6.5–6.8, the dendritic prodrug was released via cleavage of maleic amide. The relatively large size of the micelle enabled targeting via the EPR effect. In contrast, the dendritic prodrug was small enough (~5 nm) to enable deep penetration into the tumors; after 4 h of micelle administration in pancreatic tumor-bearing mice, the prodrug was detected as deep as 200 μm from tumor vasculatures.

Size reduction can also be achieved by removing polymer coatings. Han et al. explored this behavior for HA-coated dendrimers [785]. Removal of the HA via MMP cleavage of the linker enhanced tumor accumulation over a system that did not use an MMP cleavable linker and one that did not use a HA coating. An MMP cleavable linker was also used to conjugate a PEG coating onto the TAT-PEG-DSPE micelle evaluated in Yao et al.’s study [786]. In addition to a size reduction, removal of the PEG layer also exposed TAT facilitating cellular uptake. The majority of the DDS was retained in the tumor over 96 h. In contrast, the control micelles without a removable PEG layer were rapidly drained from the tumor.

Another approach protects smaller DDS particles by linking them into larger particles until they arrive at the tumor. The breakup of these agglomerates in the ECM enables deep penetration into the tumor mass. In the work of Su et al., the authors loaded PFC and PTX onto magnetic nanoclusters, then coated the nanoclusters with lactoferrin [787]. The lactoferrin shell protected the payloads from premature release and targeted the nanocarriers to brain cancer (RG2) cells. The application of AMF for 5 min led to the disruption of the nanocarrier and evaporation of PFC. The PFC gas enabled the deep penetration of nanocarrier fragments and PTX release. PFC and extraneous stimuli-induced size change was also used in Liang et al. [788]. They conjugated the hydrophobic drug camptothecin and hydrophilic floxuridine and fabricated a micelle from the resulting amphiphile. PFC microdroplets were incorporated in the micelles, enabling the use of the system as an ultrasound contrast agent. Upon application and removal of ultrasound, the micelles broke apart and then reformed without the PFC microdroplets. The reduced size of the system enhanced its extravasation and accumulation in tumor tissue. 4T1 tumor-bearing mice in vivo testing demonstrated that the application of ultrasound to the system reduced the fraction of micelles trapped in the liver, and the accumulation of the conjugate in the tumor was four times that of PFC-CF micelles without ultrasound applied.

#### 3.8.3. Stimuli-Induced Surface Property Changes

Nanoparticles with hydrophobic and weakly positive to neutrally charged surfaces show enhanced cellular uptake over those with hydrophilic and negatively charged surfaces. Thus, altering the surface properties after the DDS has entered the tumor ECM is the third technique to enable stimuli-induced targeting of DDSs. The pH-induced cleavage of bonds to expose positively charged residual groups or hydrophobic groups can be used to endow DDSs with stimuli-induced surface property changes. Dimethylmaleic amide is readily hydrolyzed at a pH of 6.8, exposing a positively charged amine group and has found use in DDSs that incorporate stimuli-induced surface property changes to enhance uptake. Sun et al. utilized a PCL-PEG block copolymer with dimethylmaleic amide as the link between the blocks [790]. When docetaxel (DTXL) micelles entered the tumor ECM, cleavage of the bond removed the PEG group exposing the amine. The smaller particle with a weakly positive surface charge exhibited roughly twice the cellular uptake as a control micelle that did not use the cleavable bond. In this system, the removal of the PEG block also decreased the size of the DDS, further enhancing the uptake of the system. The combination of reduced particle size and weakly positive surfaces was also explored by Feng et al. [784]. They conjugated the prodrug cisplatin (IV) to amine-functionalized carbon dots (CDs), residual amine groups resulted in a positive charge on the surface. The drug-loaded CDs were then encapsulated in the core of a PEG-poly(allylamine)-based micelle in which DMMA was conjugated to amine groups on the copolymer. Hydrolysis of DMMA in tumor ECM resulted in dissociation of the micelle and release of the drug-loaded CDs. The small size and positive charge of the CDs facilitated deep penetration and cellular uptake of the system. Cisplatin was released once encountering the high levels of GSH in cancer cells. In the assay to A2780 cells, significant cytotoxicity was observed on the nanocomposite, while the cell viability was intact when incubated with the control non-pH-sensitive nanocomposite.

Enzymes overexpressed in tumor ECM can also be used to alter surface properties and enhance cellular uptake. Ge et al. explored the removal of hydrophilic shells via enzymatic reactions as a mechanism to promote both endocytosis and endosomal escape [536]. In their work, a peptide substrate of MMP-2 was used to link a hydrophilic PEG block and a modified poly(aspartamide) block (hydrophobic) with abundant amine groups. Removal of the PEG in tumor ECM via enzymatic cleavage of the peptide linker exposed the less hydrophilic positively charged core, which promoted endocytosis and facilitated endosomal escape. In a later study, a micelle that retained an RGD ligand as the peptide residue after MMP-2 cleavage was developed [329]. Exposure of the RGD ligand further enhanced endocytosis.

Another technique to enhance uptake via stimuli response is to recover the fusogenic properties of lipids used in liposome-based nanocarriers. Hatakeyama et al. used an MMP-cleavable peptide linker to attach PEG to the fusogenic lipid DOPE [789]. They evaluated liposome DDSs using both PEG-peptide-DOPE and PEG-DSPE lipids for delivery of luciferase-silencing siRNA. In luciferase-expressing HT-1080 (HT1080-luc) cell assays, they found that systems containing the cleavable PEG coating performed better than those without cleavable PEG coatings. The performance of the PEG-peptide-DOP-based DDS in vitro was not as good a system without PEG coatings, possibly due to the need to remove the coating to enable cellular uptake. However, in vivo tests on HT1080-luc tumor-bearing mice indicated that the combination of the protective PEG coating and the recoverable fusogenic lipid behavior of DOPE was superior to other formulations in terms of both tumor accumulation and luciferase activity. Furthermore, biodistribution analysis showed long circulation time and reduced accumulation in the liver and spleen.

Exogenous stimuli have also been used to control surface properties to enhance targeting. Na et al. used an elastin-like thermo-responsive polypeptide to enhance cellular internalization of a liposome [600]. They attached the polypeptide to the terminal end of the PEG-DSPE lipid included in the liposome formulation. In neutral pH and physiological temperature, the peptide adopted an expanded conformation due to its hydrated state, while at elevated temperature, the peptide dehydrated and aggregated on the lipid surface. The hydrophobic surface promoted the internalization of the liposome but did not trigger drug release. The significantly enhanced fluorescence in cells, observed under CLSM during in vitro HeLa cell testing, reflected the enhanced cellular uptake. Additionally, they demonstrated that the transition temperature of the system was reduced from 43 to 40 °C in the presence of serum. Various proteins and electrolytes moderated the interaction between the peptide and water, suggesting that further studies of the interaction of thermally responsive liposomes and physiological medium are required to enable the rational design of these systems.

## 4. Perspective and Outlook

Despite the significant advancements in stimuli-responsive DDSs, several critical challenges remain. The current limitations and concerns related to each stimulus are discussed in the corresponding sections above and summarized in Table 10. However, in addition to stimuli specific issues, there are additional overarching problems that need to be addressed before the tremendous number of proof-of-concept studies conducted can be translated into approved drug delivery systems [14]. Fundamental issues such as immunogenicity, long-term toxicity, and excretion pathways remain a concern for many DDSs. While many proof-of-concept studies are conducted each year, they often do not fully address questions relating to the feasibility of their use in clinical settings. For example, systems triggered by endogenous stimuli need to be able to respond appropriately to the heterogeneous characteristics of tumors, while for systems triggered by exogenous stimuli, questions such as whether the penetration depth required can be achieved and if the exposure period is safe need to be carefully considered.

**Table 10:** Summary of advantages and limitations of stimuli used for responsive DDSs.

Stimuli	Advantages	Limitations
pH	Reduced pH is common in tumors and varies with cell vesicles enabling response to be targeted	pHex varies from between tumor types and even within a tumor. Off-target effect in inflammation lesions and endosomes of normal cells
Redox	Endogenous factor that can also be induced by exogenous stimuli Capable of consuming GSH (GSH-responsive groups)	Off-target effect of ROS-responsive systems Off-target effect of GSH-responsive systems, particularly in hepatocytes GSH-responsive systems require endosome escape to reach high-level GSH in the cytosol
Enzyme	High specificity Overexpressed enzymes present in tumor ECM and within cancer cells	Enzyme dysregulation can be dramatically different between tumors Many active hydrolases are also present in the liver
Thermo	Can directly accelerate drug release Can enhance sensitivity to radiotherapy and chemotherapy	Endogenous temperature increase minimal and widely variable Off-target effect due to inflammation-induced temperature elevation
Magneto	Can be used to guide biodistribution and cellular uptake Can be applied to deep tumors	Reduced magnetization of MNP incorporated in nanocarriers Safety concerns of high MNP dosage and intense AMF Expensive and complicated magnetic field setup
Photo	Rapid response Precise temporal and spatial control	Safety concern of short wavelength and intensive irradiation Biocompatibility, biodegradability, and long-term safety of complex photo-responsive materials and their degradation products Limited penetration depth
US	Deep penetration Complex responsive materials not required Capable of facilitating tissue penetration and cellular uptake	Low US sensitivity for small DDSs Safety concerns for some materials used to enhance sonosensitivity Requirement of a “non-gassy” pathway Safety concerns of intense ultrasound Requirement of an expensive and complicated monitor (imaging) system setup (HIFU application)

New techniques and concepts have emerged in recent years. Many multi-responsive platforms have been reported to address heterogeneous characteristics of tumors, but responsiveness to multiple stimuli does not necessarily improve therapeutic efficiency; instead, it might enhance off-target effects. Perhaps a better strategy is a “logic” response (Table 11), i.e., the release does not occur until multiple stimuli are present [117,157,328]. This can make the delivery more precise, particularly when both endogenous and exogenous stimuli are required [159,464,791]. The demands placed on DDSs vary depending on how far along the path to the ultimate site of release they have traversed. An exciting possibility to address this problem is the concept of multi-step responsiveness in which the characteristics of the nanocarrier change in response to local stimuli in a fashion that drives the DDS to the next stage of delivery, as discussed in Section 3.8. The combination of platforms holds promise to enable multi-step responsiveness. For example, stability is improved, and premature release is reduced, with a core-shell structured combination of some polymer nanoparticle, MSN, and drug conjugate-based systems [624,654,725]. The use of a lipid membrane or a biocompatible polymer coating on many systems has been shown to enhance circulation time [279,725], and functional groups decorated on these organic layers can enable targeting [792] or release [465,627,793,794].

**Table 11:** Drug delivery systems with “logic”-based release behavior.

Stimuli	Strategy	Testing	References
pH + gelatinase	pH-responsive polyelectrolyte layer protects gelatin-based nanocarrier from gelatinase present in the liver and is dissociated in the weakly acid condition of tumor ECM.	In vivo: S180 tumor	[157]
pH + hyperthermia	LCST polymer-based nanogel maintains structure until significant shrinkage occurring in the presence of both protonation and temperature elevation.		[464]
pH + photoirradiation	pH-responsive gatekeepers and photo-responsive gatekeepers are decorated on the pores of MSN sequentially. Payloads can be released only when both stimuli present.		[791]
GSH + azoreductase	The micelle-based system contains both a disulfide-bridged amphiphilic prodrug and azo bond-bridged amphiphiles. The conjugated drug can be released only when both GSH and azoreductase are present to induce dissociation of the micelle.	in vitro: HepG2 and EC in vivo: H22 tumor on mice	[328]
GSH + hyaluronidase	A liposome composed of a disulfide-containing lipid is coated with a HA layer. The payload is released only when the HA is degraded by hyaluronidase, and the liposome is dissociated due to GSH cleavage of the disulfide bond.	in vitro: A549 in vivo: LLC tumor on mice	[117]
Protease + photoirradiation	A photo-cleavable group protects crosslinks based on a protease substrate. The polymeric nanoparticles can not be degraded by protease until the system is activated by photoirradiation to remove the protective group.	in vitro: HeLa cell	[159]

Delivery of a combination of multiple therapeutics or combining a therapeutic with imaging characteristics is also meaningful as these systems can synergistically enhance therapeutic efficacy. Systems that can effectively deliver multiple therapeutics have the potential to overcome issues such as multidrug resistance, IFP, and the physical barrier in tumor ECM [43,269,280]. Similarly, the ability to precisely deliver immunotherapy agents represents a new application for nanocarrier-based DDSs, the characterization of tumors [307,795,796,797]. The better visualization achieved by including imaging agents with the nanoplatforms will enable the key properties of tumors to be better characterized for each patient. Understanding these features will contribute to further personalization of cancer treatment [15]. Systems that incorporate imaging features will enhance the traceability of drug delivery systems in vivo, enabling a better understanding of the interactions of these systems with physiology leading to further improvements in DDSs.

Another issue hampering the development of DDSs is that the complexity of the biological system makes it hard to assess their performance objectively. The response to nanocarriers in blood plasma is difficult to reproduce in simulated environments, i.e., the serum proteins, MPS cells, various electrolytes, and the dynamic flow rate of heart pumped blood in a flexible circulatory system are difficult to reproduce in a lab setting, and may significantly impact the release profile. For example, in vivo, a protein corona forms on many nanocarriers’ surfaces, affecting their physiochemical properties and enhancing MPS clearance. Once DDSs enter tumor vasculatures, the gap dimensions on vessel walls, increased IFP, and other tumor morphologies can prevent them from undergoing extravasation and deep penetration. The heterogeneity of tumors leads to different receptor and enzyme expressions, which may reduce the efficacy of target ligands and passivate responsiveness. After nanocarriers internalized into cancer cells, the difficulty in endo/lysosome escape and multidrug resistance may further impair the effectiveness of medicines. Thus, in vivo performance is difficult to predict from in vitro tests. Even when both in vitro and in vivo experiments are conducted, the differences in nanocarrier dosage, cell types, animal status, and xenograft protocols make it difficult to evaluate their therapeutic efficacy impartially. These differences make comparisons between systems problematic. In addition, even the excellent performance of a DDS exhibited during in vivo testing is not sufficient to indicate that the system would exhibit improvement over current clinical treatments, as the experiments are usually conducted using solid tumors inoculated in animals as a model. However, locally confined solid tumors can be effectively ablated via surgery or radiotherapy, while metastatic tumors are the leading cause of severe cancers and death. DDSs tested on solid tumor models may turn out to be inefficient in the treatment of metastatic tumors. The tumors in rodents in labs are relatively homogeneous, grow rapidly, and have a pronounced EPR effect. They are very different from the tumors naturally occurring on patients, which possess vast heterogeneity and not as strong EPR effect [50]. This difficulty in evaluating therapeutic efficiency is one cause of the contrast between the large number of proof-of-concept reports every year and the limited number of approved systems to date. One way to address this issue is to develop quantitative measurements such as the therapeutic index, which reflects both the effectiveness and safety of medications [266,306]. Another approach would be to increase the number of comparative investigations reported [251,467,484]. More efficient and advanced characterization techniques such as artificial 3D tumor models and organ-on-a-chip systems should be developed and adopted [798,799]. These systems would increase quantitative measurements enabling computational science and data science to be utilized to boost ability to design and optimize drug delivery systems [800].

Finally, two rules should be kept in mind for researchers. First, the final therapeutic efficiency depends on the efficiencies of every step during a five-step cascade: blood circulation, tumor accumulation, tumor penetrations, cancer cell internalization, and payload release [6]; limitations in any of these steps reduces the overall efficiency. Researchers should keep the entire process in mind during DDS development. Second, keep the DDS design simple. Technical progress has introduced more and more novel functional materials for use as, or to adapt to, DDSs. However, complex designs raise issues with uncertainty as the system travels along the delivery route. For many of the new novel materials, biological interaction at many points in this path has yet to be evaluated. Furthermore, complex design makes synthesis and manufacture difficult. In other words, the trade-off between complexity and multifunctionality should be carefully considered and realized.

The authors hope that this review helps to reduce interdisciplinary barriers that exist in DDS research. The hope is that the work will enable researchers to identify links between their research and that of other researchers exploring stimuli-responsive DDSs. We believe that progress in nanotechnology and oncology, as well as the appropriate combination of various drug delivery and stimuli-responsive techniques, will enable well-engineered nanoplatforms that can achieve effective cancer treatment. These systems will not only smartly deliver therapeutics, but also possess additional functionality and behave as multifunctional systems providing therapeutic, diagnostic, and imaging capabilities.

## Data Availability

No new data were created or analyzed in this review. Data sharing is not applicable to this article.
